# Manganese Ferrite Containing Glass-Crystalline Materials—Phase Composition, Microstructure and Magnetic Properties

**DOI:** 10.3390/ma19091771

**Published:** 2026-04-27

**Authors:** Petar Takov, Ruzha Harizanova, Irena Mihailova, Pavlina Bancheva-Koleva, Georgi Avdeev, Daniela Paneva, Zara Cherkezova-Zheleva, Milena Georgieva, Todor Karadimov, Christian Rüssel

**Affiliations:** 1 Physics Department, University of Chemical Technology and Metallurgy, 8 Kl. Ohridski Blvd., 1756 Sofia, Bulgaria; petar.v.takov@gmail.com (P.T.); bancheva@uctm.edu (P.B.-K.); 2 Department of Silicate Technology, University of Chemical Technology and Metallurgy, 8 Kl. Ohridski Blvd., 1756 Sofia, Bulgaria; irena@uctm.edu; 3 Institute of Physical Chemistry, Bulgarian Academy of Sciences, Block 11, Acad. G. Bonchev Str., 1113 Sofia, Bulgaria; g_avdeev@ipc.bas.bg; 4 Institute of Catalysis, Bulgarian Academy of Sciences, Block 11, Acad. G. Bonchev Str., 1113 Sofia, Bulgaria; daniela@ic.bas.bg (D.P.); zzhel@ic.bas.bg (Z.C.-Z.); 5 Condensed Matter Physics and Microelectronics Division, Faculty of Physics, Sofia University “St. Kliment Ohridski”, James Bauchier Blvd. 5, 1164 Sofia, Bulgaria; mgeorgieva@phys.uni-sofia.bg (M.G.); tkaradimov@uni-sofia.bg (T.K.); 6 Otto Schott Institute for Materials Research, University of Jena, Fraunhoferstr. 6, 07743 Jena, Germany; ccr@uni-jena.de

**Keywords:** manganese ferrite, magnetite, crystallization, X-ray diffraction, Raman spectroscopy, Mössbauer spectroscopy, valence state, scanning electron microscopy, magnetometry, ferrimagnetism

## Abstract

**Highlights:**

**What are the main findings?**

- New glass-crystalline magnetic materials are synthesized in the system Na_2_O-MnO-SiO_2_-Fe_2_O_3_.
- The spinel phase Mn_x_Fe_1−x_O_4_ precipitates.
- Iron occurs in both the glass and the crystals as Fe^2+^ and Fe^3+^.

**What are the implications of the main findings?**

- It is proved that the proposed set of compositions allows the preparation of glasses for up to 15 mol% Fe_2_O_3_ and spontaneous crystallization occurs for 20 and 25 mol% Fe_2_O_3_.
- The compositions chosen and the method of melt-quenching enable preparation of materials with high degree of crystallinity and uniform bulk crystallization.
- Soft ferrimagnetic materials with relatively high saturation magnetization and potential for application in electronics are obtained.

**Abstract:**

The preparation of new magnetic materials is important because of their potential application in various electronic components. In the present work, the synthesis of glass-crystalline materials in the system Na_2_O-MnO-SiO_2_-Fe_2_O_3_ prepared by applying melt-quenching is reported. The phase composition as studied by X-ray diffraction and Raman spectroscopy reveals the precipitation of monophase Mn_x_Fe_3−x_O_4_ based solid solutions. The microstructure is studied by scanning electron and optical microscopy and shows bulk crystallization and the presence of polygon-shaped as well as of dendritic crystals, depending on the iron oxide concentration and used raw materials. Mössbauer spectra show that in the amorphous matrix the Fe ions are mainly present as Fe^3+^ in tetrahedral coordination and as Fe^3+^ in a solid solution with the composition Mn_x_Fe_3−x_O_4_. The simultaneous presence of MnFe_2_O_4_ (jacobsite) and a Mn-containing solid solution based on Fe_3_O_4_ (magnetite) is suggested. The room temperature magnetic properties were studied by vibrating sample magnetometer and reveal ferrimagnetic properties for all investigated glass-crystalline materials.

## 1. Introduction

Glasses containing oxides of 3D-transition metals have been the subject of various studies aiming the preparation of glass-ceramics [1,2,3]. These materials combine the properties of the amorphous matrix and those of the crystalline phase and are interesting for application as catalysts, in biomedicine and electronics, i.e., as sensors, biosensors, supercapacitors, catalysts, nano adsorbents and in magnetic resonance imaging, etc. [3,4,5,6,7,8,9,10]. In the present study, a well-investigated base glass was used and the constituents required for crystallizing the desired magnetic phase were added. This concept enables to study the influence of the added components on the glass structure and their effect on the crystallization behavior [11]. It should be noted that the phase composition and the structure of the resulting glass-ceramics in the system Na_2_O-CaO-SiO_2_-Fe_2_O_3_ notably affect the physical properties and hence is of both scientific and technological importance. About 80% of the industrial glass production are based on the Na_2_O-CaO-SiO_2_ system. Here, flat glass and packaging glass, as well as many bioactive glasses are to be mentioned. The addition of iron oxide to the Na_2_O-CaO-SiO_2_ system and the replacement of CaO by MnO affects the structure as well as the physical and chemical properties, such as the viscosity and the crystallization behavior. In the past, the preparation of various glass-ceramic materials from iron-rich glasses with advanced functional properties has been exploited [12,13,14].

Another important factor for the preparation of materials with advanced electrical and magnetic properties is the choice of the synthesis method. Many authors report the preparation of various ferrites via sol–gel, co-precipitation, micelle, reverse micelle method and by means of solid-state reactions [15]. These routes allow to obtain chemically inert materials with tunable sizes and volume fractions of the ferrite crystals as well as with pre-desired properties. However, the cited preparation techniques have the disadvantage that very often some of the precursors remain unreacted or that the obtained materials contain a significant fraction of pores. Thus, an alternative synthesis method allowing to prepare glass-ceramics with specific compositions and properties is the melt-quenching technique. Initially, a glass is synthesized from which after applying appropriate time–temperature schedules, glass-ceramic materials are obtained [12,13,14]. The advantage of such a synthesis technique is that the obtained materials are chemically inert as well as combine the advantageous properties of the amorphous nonmagnetic matrix and the magnetic particles immobilized in it. In addition, the melt quenching methods is orders of magnitude economically more advantageous than the other methods mentioned above.

Iron- and manganese-containing crystalline and amorphous phases occur in numerous materials found in nature but nowadays they are also desirable part of industrial glasses and glass-ceramics due to the advantageous electrical, magnetic and catalytic properties of the respective materials [1,2,3,4,5,6,7,8,15,16,17,18,19,20,21]. The simultaneous presence of Fe and Mn ions has a significant effect on important physical properties of these glasses, such as optical absorption, color, thermal insulation and energy transmission. Applying controlled crystallization of glasses with high Fe_2_O_3_ concentrations allows the preparation of glass-ceramics with iron-containing crystalline phases such as magnetite, maghemite and various other ferrites such as MnFe_2_O_4_ (jacobsite) [15,16,17,18,19,20]. These phases possess interesting magnetic, electrical, catalytic, and optical properties. In addition, they show other favorable properties such as high chemical durability and biocompatibility. In summary, they offer advantageous properties for numerous potential applications [2,21,22]. Furthermore, the presence of Mn in the ferrite composition often results in monophase materials with magnetic properties comparable to those of magnetite and avoid the problem that magnetite is usually present as a solid solution with maghemite (γ-Fe_2_O_3_). Glasses with high Fe_2_O_3_ concentration can also be prepared using different industrial wastes, such as metallurgical slags and slimes [23,24,25,26].

The physical properties as well as the crystallization behavior of 3d-transition metals containing systems mainly depend on the valence states of the transition metal ions occurring in the respective glasses. Since manganese ions in acidic silicate glasses are mainly present as Mn^2+^ [27,28,29], the attention in the present research is directed to the valence states and coordination numbers of Fe ions. Generally, in all glass, iron is present as ferrous, Fe^2+^ and ferric, Fe^3+^ ions. The redox state in which Fe-ions are present in glass is affected by various factors, such as: melting temperature [30], oxygen activity of the melt [31], iron concentration [32], base glass composition [30,31,32,33,34], cooling rate [35] and the addition of oxidizing or reducing components as well as by the crucible material. In presence of other polyvalent compounds, the redox ratio [Fe^2+^]/([Fe^2+^] + [Fe^3+^]) may also change with temperature due to redox reactions [27,36,37,38]. Fe- ions act as intermediates in the structure of silicate glasses. Fe^2+^ is mainly six-fold coordinated and serves as network modifier [39]. By contrast, Fe^3+^ is mainly four-fold coordinated and acts as network former [40]. However, in numerous studies, a minor quantity of Fe^2+^ in tetrahedral and also some Fe^3+^ in octahedral coordination were reported [16,41]. In addition, also pentahedrally coordinated Fe^2+^ and Fe^3+^ ions in silicate glasses might occur [41,42,43,44,45]. In soda–lime–silicate glasses with more than 1 mol% Fe_2_O_3_ [40,46], iron containing clusters are formed, i.e., bonds of the type Fe^2+^–O–Fe^3+^ and Fe^3+^–O–Fe^3+^ occur. The formation of clusters containing more than two iron atoms besides tetrahedrally coordinated isolated Fe^3+^ ions was proved in several alkali–lime–silica glasses containing iron oxide concentrations in the range from 0.1 to 2 mol% using EPR spectroscopy [40]. In the glasses from the system Na_2_O-CaO-SiO_2_ with 25 mol% Fe_2_O_3_, according to Weigel et al. [42], Fe^3+^ occurs in the coordination numbers of 4, 5 and 6. In comparison to tetrahedrally coordinated Fe^3+^ ions, which are part in the silicate network, those Fe^3+^ ions with higher coordination numbers are not uniformly distributed. The simultaneous presence of clusters and isolated iron-polyhedra is observed in float glass even for Fe_2_O_3_ concentration as low as 0.19 mol% [47].

Crystalline phases with outstanding magnetic properties are for example magnetite, Fe_3_O_4_ and jacobsite, MnFe_2_O_4_ [17,18,19,20]. Glasses in the system (100 − x)(0.16Na_2_O∙0.10 MnO∙0.74 SiO_2_)∙× Fe_2_O_3_, and glass-ceramics derived hereof were already analyzed [12,13,14,48] with respect to their elemental composition, phase composition [14,48] and microstructure. X-ray amorphous materials were obtained with up to 15 mol% Fe_2_O_3_. However, in samples with 15 mol% Fe_2_O_3_, using Fe_2_O_3_ as raw material, i.e., prepared under oxidizing conditions, electron microscopy evidenced the occurrence of nanosized crystals [12]. By contrast, amorphous samples of the same cationic composition were obtained, if the preparation was performed under reducing conditions using FeC_2_O_4_·2H_2_O as a raw material. As the latter samples possibly possess the highest iron oxide concentration before spontaneous crystallization occurs, they were studied with respect to their crystallization behavior. However, the concentrations of Fe_2_O_3_ and MnO in these glasses were not high enough to enable the preparation of glass-ceramics with high MnFe_2_O_4_ volume concentrations [14]. For these reason, further crystallization studies were carried out using partially crystallized [12] samples with higher iron and manganese concentrations. In the prepared as cast materials, the valence states and coordination numbers of Fe ions were studied.

The present study is dedicated to the preparation of glasses in the system (100 − x)(0.16Na_2_O∙0.10 MnO∙0.74 SiO_2_)∙× Fe_2_O_3_ with x = 15, 20 and 25 mol% by applying a traditional melt-quenching technique. The phase composition is studied by X-ray Diffraction (XRD) and reports on the precipitation of Mn_x_Fe_3−x_O_4_-based solid solutions. The phase composition is also probed by Raman spectroscopy. The microstructure is studied by Optical Microscopy (OM) and Scanning Electron Microscopy (SEM) combined with Energy Dispersive X-ray Spectroscopy (EDXS). Mössbauer spectroscopy is used to gather information on valence state and coordination of the Fe ions as well as on precipitated crystals. The magnetic properties are studied by vibrating sample magnetometer at room temperature.

## 2. Materials and Methods

### 2.1. Materials

A set of compositions (100 − x)(0.16Na_2_O∙0.10 MnO∙0.74 SiO_2_)∙× Fe_2_O_3_ with x = 15, 20 and 25 mol% was prepared by applying a conventional melt-quenching technique from the reagent grade raw materials Na_2_CO_3_, SiO_2_, MnCO_3_ (Thermo Fisher Scientific, Waltham, MA, USA). Additionally, for the samples in the following denoted as “oxidized” Fe_2_O_3_ (Thermo Fisher Scientific, Waltham, MA, USA).and for the samples designated as “reduced” FeC_2_O_4_∙2 H_2_O (Thermo Fisher Scientific, Waltham, MA, USA) were used as raw materials. Homogenized batches of 100–300 g were melted in air at a temperature of 1400 °C supplied for 1.5 h using Pt- or SiO_2_-crucibles and a MoSi_2_ furnace. The melts were either quenched on a Cu-block without pressing or poured onto a pre-heated graphite mold and subsequently transferred to a muffle furnace kept at 480 °C for 10 min. Switching off the furnace allowed the samples to cool to room temperature with a rate of around 2 K/min. The obtained samples are denoted according to their Fe_2_O_3_-contents of x = 15, 20 and 25 mol% as 15F, 20F and 25F for the oxidized and 15FR, 20FR and 25FR for the reduced samples, respectively.

The mol% compositions of the synthesized oxidized and reduced samples are summarized in Table 1.

### 2.2. Methods

X-ray diffraction (XRD) was carried out in the Bragg–Brentano (θ-2θ) setup, using the X-ray diffractometer Philips PW1830/PW1050 (Philips Analytical, Eindhoven, The Netherlands) with CuKα radiation, (λ = 1.541874 Å) and a Ni filter in the 2θ-range from 10 to 70°. The phase and crystal structure analyses were performed using the X’Pert HighScore Plus software (version 4.0, 1 December 2013, licensed to: Institute of Physical Chemistry, BAS; Database PAN-ICSD version 3.3, 29 March 2013, licensed to: Institute of Physical Chemistry, BAS (Sofia, Bulgaria)) and data from the American Mineralogist Crystal Structure Database (AMCSD) [49] and Crystallography Open Database (COD) [50]. Rietveld refinement was performed for the quantification of the crystallite sizes of the detected phases and the determination of the fractions of each present phase using HighScore Plus 4.0 software. As peak profile fitting Profile Function, Pseudo Voigt Profile with base width 4 was used.

Raman spectroscopy was performed on plane parallel polished sample surfaces by using a Raman Renishaw inVia microscope (Glocestershire, UK) with Leica DM2700 M (Leica Microsystems CMS GmbH, Wetzlar, Germany). For excitation, the 633 nm line of a helium–neon laser was used. A laser power of only 3.2 mW was supplied to avoid local overheating. The laser beam was focused by an ×50 lens which also collected the scattered light in a backscatter configuration. The illuminated spot on the surface of the specimen had a diameter of about 3 µm. The spectra were recorded in the wavenumber range from 90 to 1800 cm^−1^. The accuracy of the wavenumber determination was 1 cm^−1^.

SEM micrographs were recorded using SEM EVO 10 (Carl Zeiss GmbH, Jena and Oberkochen, Germany) equipped with an Energy Dispersive X-ray Spectrometer (EDXS) (Oxford Instruments, Xplore 30, Oxford, UK). Optical microscopy was performed in reflected light by utilizing an Axioscope 5 polarization microscope (Carl Zeiss Microscopy GmbH, Jena, Germany) with an Axiocam 208 camera (Zeiss, Carl Zeiss IQS Deutschland GmbH, Oberkochen, Germany).

Mössbauer spectroscopy was performed at room- and liquid nitrogen temperatures using a Mössbauer spectrometer, supplied by “Wissenschaftliche Elektronik GmbH, Germany”, operating in a constant acceleration mode (^57^Co/Rh source, α-Fe standard). The fitting program NORMOS (http://www.wissel-gmbh.de, accessed on 14 July 2008) was used to determine the parameters of hyperfine interactions of spectral components by fitting with Lorentzian line shapes. These parameters are isomer shift (IS), quadrupole splitting (QS), effective hyperfine magnetic field (H_eff_), line widths (FWHM), and component relative weights (G).

Magnetic measurements were performed at room temperature (RT) using a vibrating sample magnetometer (VSM) applying magnetic fields of up to 6 kOe. The glass-ceramics were powdered and pressed into cylindrical quartz containers (Ø = 3 mm, h = 20 mm) to ensure that the particles were fixed during the measurements.

## 3. Results

The sample compositions are listed in Table 1. The obtained samples were either black with glittering surface for the amorphous samples or black and opaque for the respective glass-crystalline materials and nontransparent to visible light.

### 3.1. XRD

**Figure 1:**
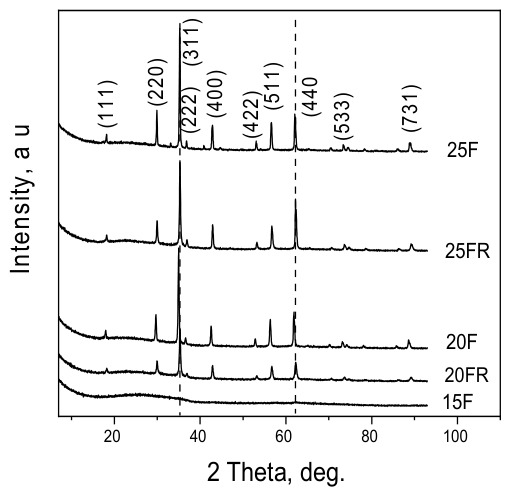
XRD patterns of samples 15F, 20F, 20FR, 25F and 25FR.

All XRD patterns were recorded from the as quenched and subsequently powdered samples. The patterns of all samples showed cubic jacobsite (MnFe_2_O_4_)–magnetite (Fe_3_O_4_) solid solution, cf. Supplementary Material S1, Figures S1–S4. In Figure 1, the XRD patterns of the glass-crystalline samples 20F, 20FR, 25F and 25FR are presented.

For the oxidized samples, larger average sizes of the crystallites and higher crystalline fractions of the precipitated spinel phase are observed than in the reduced samples. Furthermore, the average crystallite size increases with the increasing Fe-oxide concentration for one and the same raw material used for the introduction of the iron oxide to the composition. On the other hand, the unit cell parameters of the spinel phase for the oxidized and reduced samples differ. The reduced samples, 20FR and 25FR are characterized by a unit cell parameter that is closer to that of magnetite independent of the iron concentration. For the oxidized samples 20F and 25F larger unit cell parameters are determined and thus, it is assumed that the respective crystals are enriched in manganese.

Cell parameters determined by the Rietveld analysis and the ratio of amorphous to crystalline phase are given in Table 2.

### 3.2. Raman Spectroscopy

**Figure 2:**
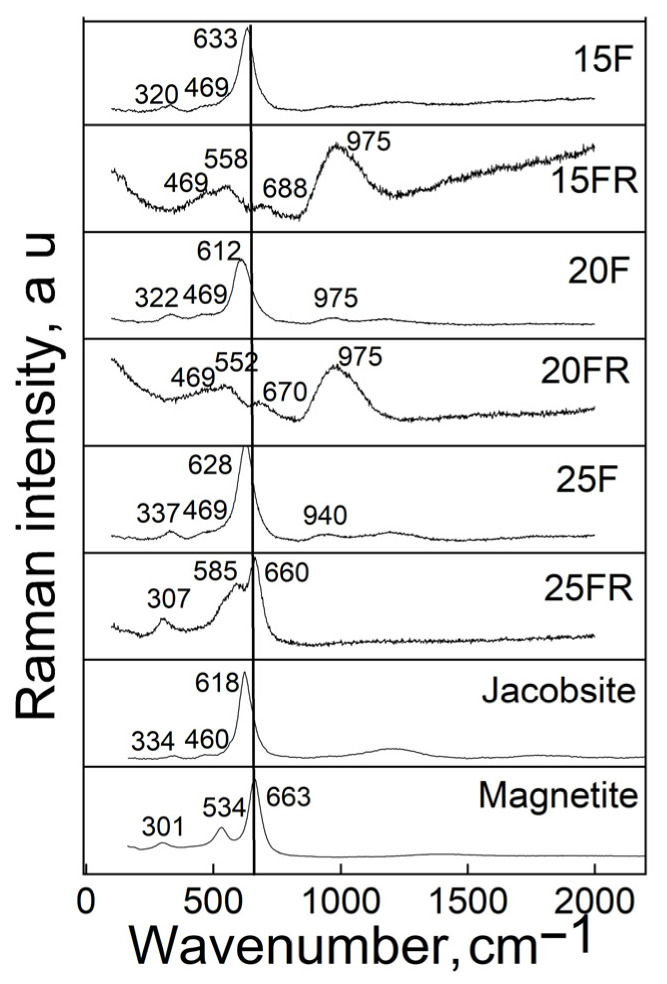
Raman spectra of the samples and of Fe_3_O_4_ and MnFe_2_O_4_ for comparison.

Raman spectroscopy was performed on plane parallel polished sample surfaces. The recorded spectra are presented in Figure 2 in comparison to the Raman spectra of magnetite and jacobsite.

The measured Raman spectra reveal the presence of peaks typical for the phases jacobsite (620–650, 340, 293, 221 cm^−1^) and magnetite (663, 540, 301, 190 cm^−1^) and no evidence of the presence of hematite, α-Fe_2_O_3_ (1320, 614, 602, 414, 294, 230 cm^−1^) is detected [51]. In the spectra of the samples with a lower degree of crystallinity also Raman peaks at approximately 460–470, 550–570 and 940–975 cm^−1^ are well visible which are attributable to structural units present in the amorphous network.

### 3.3. Optical Microscopy and SEM

**Figure 3:**
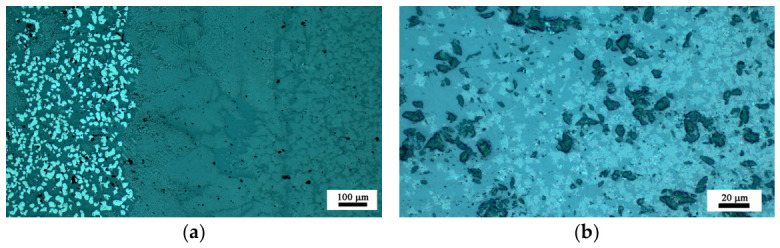
Optical micrographs of the polished surfaces for the glass-crystalline samples (**a**) 20F and (**b**) 20FR.

Information on the crystals’ morphology was obtained by Optical Microscopy and is presented in Figure 3. The sample 15F is slightly crystallized on the surface but as seen in Figure 1, it is X-ray amorphous. By contrast, sample 15FR is amorphous as witnessed by both XRD and microscopy. The samples 20F and 20FR are partially crystallized—both on the surface and in the bulk. In the reduced sample 20FR smaller and less numerous crystals occur. The crystal morphology is similar—snow-flake-like dendritic aggregates of bright appearance in both samples 20F and 20FR. In sample 20F, also polygon-shaped crystals of bright appearance occur, which are attributable to the spinel solid solution Mn_x_Fe_3−x_O_4_. In the micrographs in Figure 3a,b also the amorphous dark phase is well visible.

Additional data on the microstructure and local chemical compositions was obtained by SEM combined with EDXS, cf. Supplementary Material S2, Figures S1–S62 and Tables S1–S62. The type and morphology of the crystals present in each sample are shown in Figure 4, Figure 5, Figure 6 and Figure 7. This additional information complements results from XRD and Raman spectroscopy in order to better understand the phase composition. In the oxidized samples, two types of crystalline particles are present—star-shaped finely branched dendrites and large polygons (see Figure 4). By contrast, in the reduced compositions only the star-shaped finely branched dendrites are observed (see Figure 5). Increasing iron oxide concentration results in larger crystals and a higher volume concentration (see Figure 6 and Figure 7).

### 3.4. Mössbauer Spectroscopy

Mössbauer spectra were registered at different temperatures (in this case at RT and LNT) as a standard approach to follow the presence of relaxation processes, such as small size effects, as, e.g., superparamagnetic (SPM) behavior. While at room temperature, a doublet spectrum is observed, the spectrum observed at LNT shows a Zeeman sextet indicating the presence of nano-size particles. Spectra of the samples 15FR and 15F at room (RT) and liquid nitrogen (LNT) temperatures are given in Figure 8 as a representative example of the performed Mössbauer investigation of this series of samples. The spectra show doublets and an additional sextet component with low intensity (5%). According to the determined parameters, the existence of a superparamagnetic iron oxide-based phase with mean particle sizes less than 10 nm can be assumed. The doublet part of the spectrum is deconvoluted into two doublets corresponding to Fe^2+^ and Fe^3+^ ions. The change in the spectra for the reduced sample 15FR is due to the presence of a higher Fe^2+^ concentration. The isomer shift for both doublets is typical for tetrahedrally coordinated iron species.

Mössbauer spectra of the samples 20F, 20FR, 25F and 25FR recorded at room temperature are shown in Figure 9 and the data from the spectra deconvolution for all samples is summarized in Table 3.

### 3.5. VSM

The results from the vibrating sample magnetometer measurements are shown in Figure 10 for the glass-crystalline samples. The amorphous samples 15F and 15FR are most likely not magnetic or the possible magnetic moment is lower than the detection limit of the VSM device. The main magnetic characteristics of the investigated materials are given in Figure 10 and Table 4.

The sample 25F has the highest saturation magnetization, (the magnetization measured at 6 kOe), as it contains the highest amount of the Fe-phase. In summary, the oxidized samples have higher saturation magnetizations than the reduced samples with the same iron and manganese oxide concentrations, which is attributable to the smaller crystallite size of the reduced samples (see Table 2). The reduced volume to surface ratio in smaller crystallites results in a decrease in the overall magnetization. The coercivity, on the other hand, is strongly dependent on the individual magnetic particle sizes, the different magnetic phases present and interparticle interactions, which was not further investigated.

## 4. Discussion

The processing of the data from the XRD measurements (see Table 1), of the glass-crystalline samples 20F and 25F resulted in cell parameters of a = 8.4499 and 8.4521 Å, respectively. Yamzin et al. [52] reported almost the same parameter (a = 8.4500 Å) for a phase with the stoichiometry Mn_0.43_Fe_2.57_O_4_, corresponding to ICDD 01-089-2807. Thus, samples 20F and 25F are assumed to possess similar stoichiometry. On the other hand, the cell parameters for the reduced samples 20FR and 25FR are significantly smaller, a = 8.4268 and 8.4219 Å, respectively. These two cell parameters rather resemble the cell parameter of magnetite, Fe_3_O_4_ (a = 8.396 Å) as described in [53]. Furthermore, the determined average crystallite sizes by applying the Scherrer equation [54] witness that the oxidized samples have larger crystallites. An increase in the iron oxide concentration leads to a larger fraction of the crystalline phase and to larger crystallite sizes. The latter is attributable to the lower melt and glass viscosity caused by the higher Fe concentrations which facilitates the crystal growth. However, the average crystallite sizes and crystalline phase fractions of the reduced samples are always smaller for one and the same Fe concentration which is attributed to the presence of more Fe^2+^ ions in the reduced samples and thus, to a change in the glass structure in such a way that the network connectivity decreases. This should result in a higher nucleation rate in the reduced samples. The latter will lead to more nucleation centers and smaller growth possibilities due to crystallization fronts collision. Thus, the oxidized samples contain larger crystals with higher fraction of the crystalline phase which is witnessed by the intensity of the peaks in the XRD pattern and the data in Table 2 as well as by the Raman spectra.

The Raman spectra shown in Figure 2 support this idea, as it is seen that the reduced glass-crystalline samples show much weaker peaks of the formed crystalline phase. The samples 20FR as well as the glassy sample 15FR show relatively intense peaks at approximately 460–470, 550–570 and 940–975 cm^−1^ which are ascribed to the Si-O structural units of the amorphous matrix [55,56,57]. Actually, the vibrations at about 470 cm^−1^ should be due to rocking Si-O-Si vibrations, those in the range 550–570 cm^−1^ are assigned to bending vibrations of Si-O-Si but are also due to bonds of the type Mn-O and Fe-O. Bands in the range 940–980 cm^−1^ are attributable to asymmetric stretching Si-O-Si vibrations. The bands characteristic for the magnetite and jacobsite phases were assigned, according to [58,59]. It is worth mentioning that for the 25FR sample, bands attributed to the vibrations of the amorphous phase are almost missing. According to the SEM micrographs and the magnetic measurements, a large crystalline fraction of very fine dendrites is present in this sample and the Raman and Mössbauer spectra suggest that they can mainly be attributed to the Fe-rich Mn_x_Fe_3−x_O_4_ solid solutions. The latter observation could also be the reason for the smaller saturation magnetization of the sample 25FR in comparison to sample 20F where the average crystallite size is larger though the crystalline fractions are equal from XRD.

In Figure 2, it is further seen that the Raman spectrum of the sample 15F contains a well-defined peak at about 633 cm^−1^ and two less intense broad peaks at 320 and 469 cm^−1^ which could be attributed to the vibrations of the transition metal ions constituting a solid solution of the type Mn_x_Fe_3−x_O_4_. This result is supported by the data from the Mössbauer spectra recorded at room temperature, cf. Table 3 where only two doublets are observed. At LNT, however, a sextet is observed supposedly due to the presence of SPM iron oxide-based particles which also corresponds to earlier SEM observations [12]. These peaks occur in the Raman spectrum of sample 15F, but are almost missing in the reduced sample 15FR which according to XRD and Mössbauer spectroscopy (only two doublets from the spectra deconvolution registered) is amorphous. Actually, for the glass 15FR, well-visible bands centered at about 558 and 975 cm^−1^ are recorded which as explained above belong to the amorphous silicate network. The small very broad hump centered at approximately 688 cm^−1^ could be associated with bonds corresponding to the Fe –rich phase Mn_x_Fe_3−x_O_4_.

As known from the literature, in silicate glasses, the Mn ions are mostly present as Mn^2+^, i.e., more than 95% [26,27,28,29]. Nevertheless, a temperature dependent equilibrium is established:

Mn^2+^ + Fe^3+^ = Mn^3+^ + Fe^2+^

On the other hand, the presence of more Fe^2+^ in the reduced samples, as also confirmed by Mössbauer spectroscopy, could result in competition between the Mn^2+^ and Fe^2+^ ions to occupy the divalent positions in MnFe_2_O_4_, thus, shifting the composition of the formed solid solution Mn_x_Fe_3−X_O_4_ to the Fe-richer one, as revealed in Table 1. It should be noted that this tendency is better observed for the 20FR sample than for the 25FR sample since the higher Fe concentration leads to a larger number of octahedrally coordinated Fe^2+^ ions which remain in the amorphous matrix and thus, decrease the network connectivity. It could further be suggested that in the reduced samples, the nucleation rate is higher and thus, the number of growing crystals is larger which results in smaller particles due to colliding crystal growth fronts. The presence of Fe^2+^ ions can explain the decrease in the relative weight of the doublets attributed to the Fe^3+^ ions in tetrahedral coordination in the spectrum of the sample 25FR in comparison to that of the Fe^2+^ ions in octahedral coordination for the same glass-crystalline material. It is also smaller than the respective doublet relative weight of the sample 20FR. The doublets assigned to Fe^3+^ in tetrahedral coordination are common for all the samples investigated by Mössbauer spectroscopy, however the Fe^2+^ doublet does not appear in the oxidized samples.

**Figure 4:**
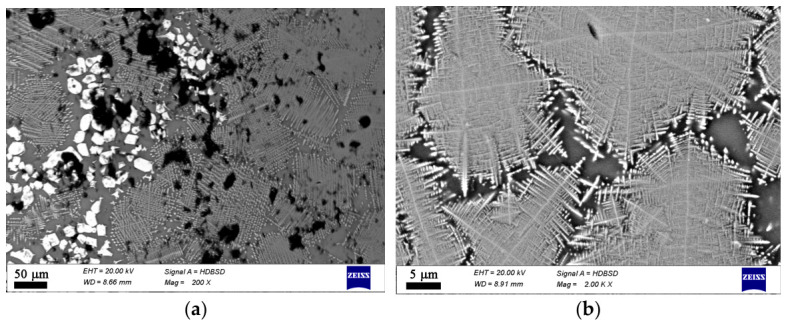
SEM micrographs of a polished surface of sample 20F. (**a**) lower magnification, (**b**) higher magnification.

**Figure 5:**
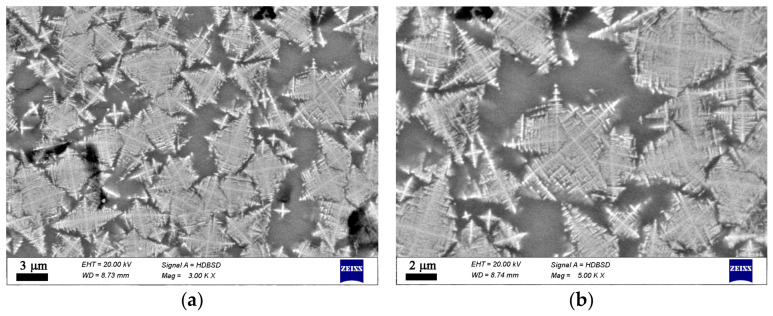
SEM micrographs of a polished surface of sample 20FR. (**a**) lower magnification, (**b**) higher magnification.

**Figure 6:**
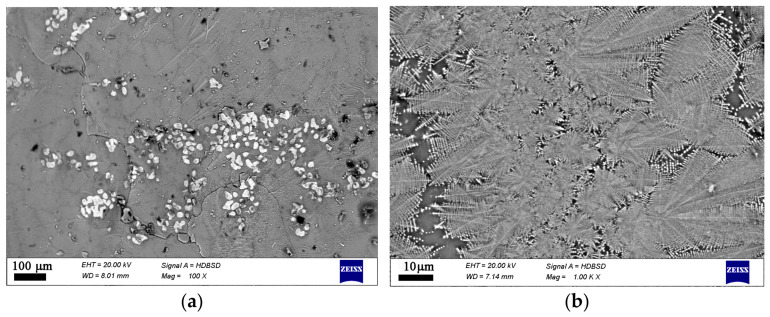
SEM micrographs on a fractured cross section of sample 25F. (**a**) lower magnification, (**b**) higher magnification.

**Figure 7:**
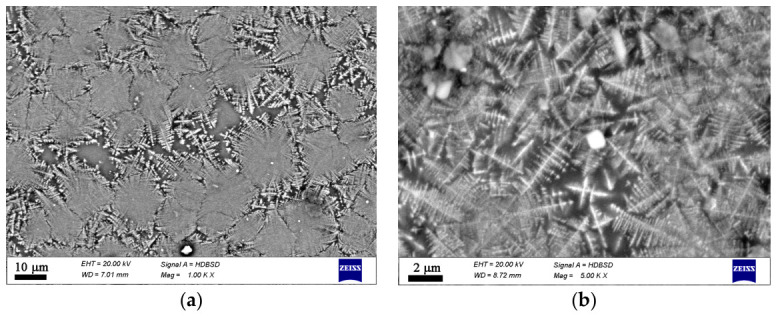
SEM micrographs on a fractured cross section of sample 25FR. (**a**) lower magnification, (**b**) higher magnification.

**Figure 8:**
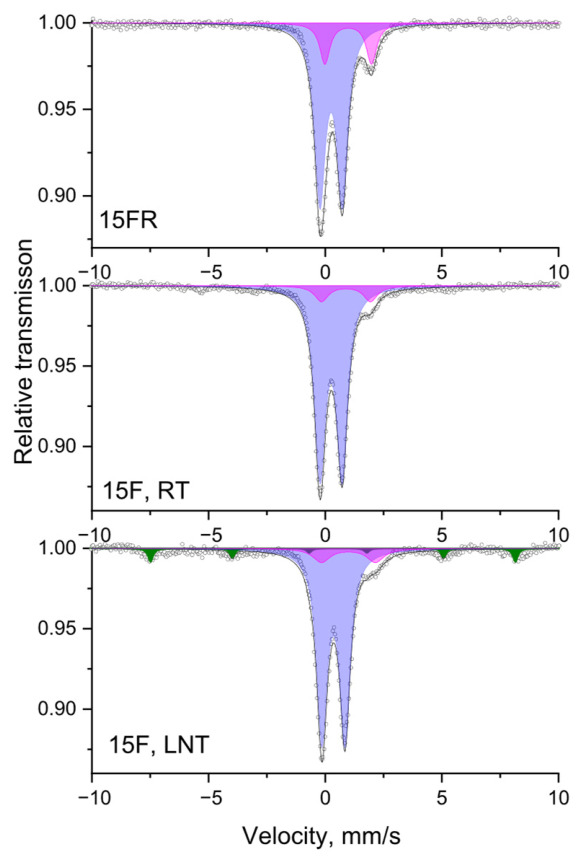
Mössbauer spectra of the samples 15FR and 15F at room (RT) and liquid nitrogen (LNT) temperatures. Sextet component: Sx—SPM iron oxides—colored in olive. Doublet components: Db1-Fe^3+^—colored in lilac; Db2-Fe^2+^—colored in magenta.

In the microstructure of the studied samples recorded by Optical Microscopy and SEM two morphological types of crystals are observed as shown in Figure 3, Figure 4, Figure 5, Figure 6 and Figure 7. While the sample 15F is crystallized at the surface and only few crystals are found in the bulk, the samples 20F, 20FR and 25F, 25FR contain a large number of crystals both at the surface and in the bulk. SEM micrographs correlate well with the crystalline phase fraction determined by XRD (see Table 2). In the 20F sample, the crystalline phase fraction is 16 wt% while for the 25F sample it is already 18 wt%. The polygons are, however, not observed in the reduced samples. The EDXS point analyses performed on the amorphous matrices of the glass-crystalline samples confirm the observations of Mössbauer spectroscopy that some of the Fe ions are still present in the amorphous phase. Actually, the EDXS analyses of the dendrites are only qualitative since the structure widths are relatively small. Hence, to detect a signal only from them which is not superimposed by signals attributed to the matrix is hardly possible. The ratio Fe/Mn detected in the dendrites of the reduced sample 20FR was approximately 8 while for the same dendritic structures in the oxidized sample 20F, this ratio was about 4.5. In the polygon-shaped crystals of the oxidized specimens F25 the ratio is similar compared to that of the dendrites in the reduced sample 20FR, i.e., Fe/Mn is approximately 8–9. According to the formed microstructure, it is assumed, that the polygon-shaped crystals were first precipitated and the dendrites start to grow afterwards. This hypothesis can be supported by the small angle X-ray scattering experiments performed on the heat-treated and crystallized samples from the 15FR glass as reported in [13]. In this Ref. [13], the occurrence of core–shell structures with almost pure Fe_3_O_4_ core and a thin shell with the composition Mn_x_Fe_3−x_O_4_ is reported. These results induce the suggestion that in the present glass-crystalline samples, the first precipitated crystals are from the magnetite type, i.e., Mn_x_Fe_3−x_O_4_ (with minor MnO concentrations) and subsequently the Mn-enriched Mn_x_Fe_3−x_O_4_ crystals start to grow, as shown in Supplementary Material S2. Here the EDXS point analyses show that the centers of the polygons for the sample 20F are enriched in Fe, while the polygon periphery shows Fe concentrations closer to that of the amorphous matrix, i.e., a ratio of about 5. In any case, the amorphous matrix contains all the elements from the samples. However, the composition of the amorphous phase in the sample 20F is characterized by an Fe/Mn ratio larger than that for the dendrite crystals, i.e., the crystals in the oxidized samples contain less Fe than those in the reduced samples. These results from the qualitative elemental SEM analysis correlate well with the Raman spectra and the findings of the other methods in this study. The sample 25F has more complex behavior, i.e., a ratio Fe/Mn for the polygons, dendrites and the amorphous phase of about 7.5, 6.5 and 8, respectively. For the 25FR sample the data obtained for the ratio Fe/Mn for dendrites and amorphous matrix are approximately 7 and 8, respectively.

VSM measurements show that increasing iron oxide concentration leads to a higher maximal magnetization for the oxidized samples while using iron oxalate as a raw material has a complex influence on the magnetic properties. As shown in Figure 10, the reduced samples as well as the sample 20F have lower saturation magnetizations but higher coercivities than sample 25F. This could be a result of various factors such as different interparticle interactions, which is strongly dependent on the average distance between magnetic particles in the nonmagnetic matrix or the presence of different magnetic phases. Furthermore, as also suggested from the EDXS data, the polygons contain more Fe than Mn, i.e., the composition of the spinel ferrite Mn_x_Fe_3−x_O_4_ is shifted to lower × values. Here also, the possibility for the existence of several generations of dendrites should be considered, i.e., larger ones which contain more Fe and finer ones which composition is closer to or even coincides with that of the pure MnFe_2_O_4_. The latter observation is also consistent with the results from the deconvolution of the Mössbauer spectra, see Table 3. All measured glass-crystalline samples have saturation magnetizations lower than the saturation magnetization of pure bulk magnetite (~90 emu/g) and that of pure bulk jacobsite (80 emu/g) [60,61]. The reason should be attributed to the glass-crystalline nature of the current samples, the average small size of magnetic particles and the different volume fraction of the precipitated magnetic phase, which is difficult to assess.

**Figure 9:**
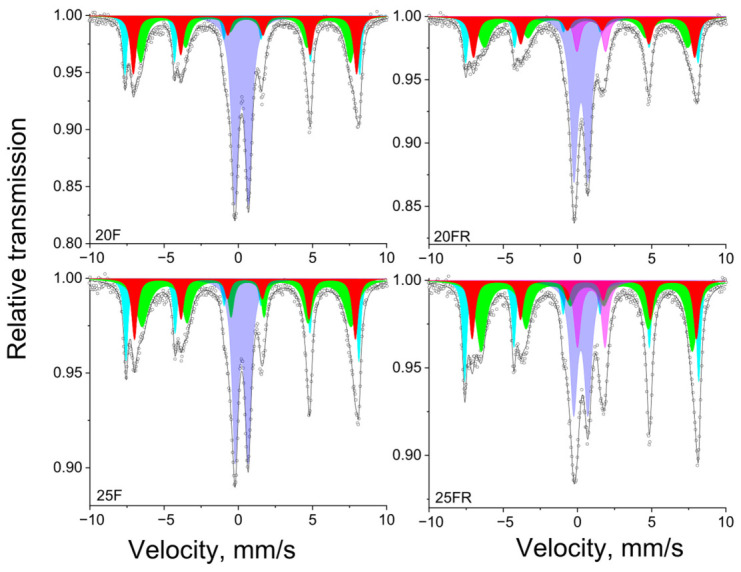
Mössbauer spectra of the glass-crystalline samples 20F, 20FR, 25F and 25FR recorded at room temperature. Sextet components: Sx1-Fe^3+^_tetra_-Mn_x_Fe_3−X_O_4_—colored in cyan; Sx2-Fe^2,5+^_octa_-Mn_x_Fe_3−X_O_4_—colored in green; Sx3-Fe^3+^-MnFe_2_O_4_—colored in red. Doublet components: Db1-Fe^3+^_tetra_—colored in lilac; Db2-Fe^2+^ _octa_—colored in magenta.

**Figure 10:**
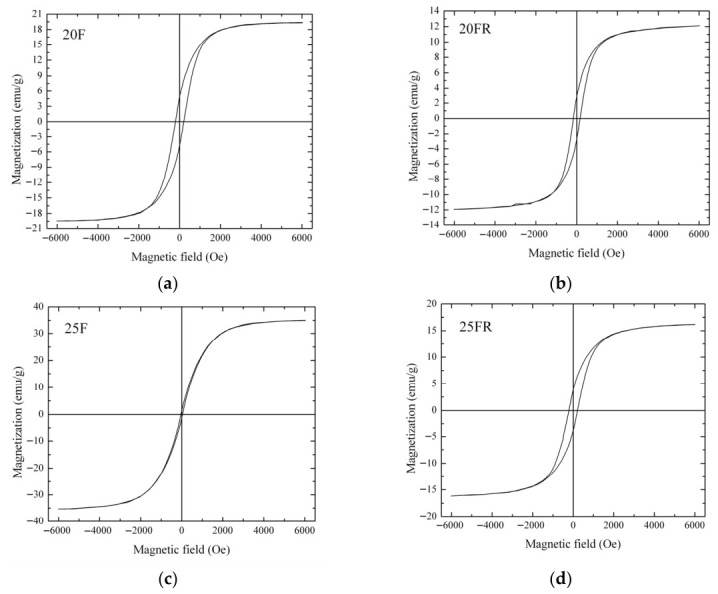
Room temperature magnetization curves of the samples (**a**) 20F, (**b**) 20FR, (**c**) 25F and (**d**) 25FR.

In the literature, numerous data on various compositions, including mixed ferrite spinels with manganese, are found [52,61,62,63,64]. As long as the ratio Mn/Fe differs from the stoichiometric one, a complex composition of the ferrites can be suggested. The appearance of sextet components in the Mössbauer spectra (Figure 9) is definitely related to the formation of the Mn_x_Fe_3−x_O_4_ spinel phase. The Fe ions occur in both tetrahedral and octahedral coordination, as well as in both oxidation states, i.e., as Fe^2+^ and Fe^3+^. Thus, the deconvolution model should, according to previous investigations [13,52,61,62,63,64,65,66], be based on the combination of components connected to the presence of members of the Mn_x_Fe_3−x_O_4_ spinel solid solution. Hence, the model for the mathematical processing of the samples 20F, 25F, 20FR and 25FR, which includes three sextets is the best fit model based on: (i). the literature data (physically meaningful models as explained above); (ii). goodness-of-fit metrics (the calculated minimal value of χ^2^ parameter); (iii). no constraints used for spectra evaluation (such as line widths, intensity ratios, etc.).

Then, the presented sextets reveal a complicated composition of solid solution members of spinel structure Mn_x_Fe_3−x_O_4_ with changing stoichiometry, where 0 ≤ x ≤ 1. The corresponding Sx1 and Sx2 parameters can definitely be associated with the presence of Fe ions situated in Fe-rich areas of spinel structure, i.e., to a phase of the magnetite-type Mn_x_Fe_3−x_O_4,_ where x is close to 0. In magnetite, the octahedral sub-lattice combines Fe^3+^ and Fe^2+^ ions and theoretically its area should be twice as large as the area of the tetrahedral sub-lattice as it was obtained for the samples 20F, 25F, 20FR and 25FR (see Table 3). In addition, Sx3 is a sum component of Fe^3+^ ions in octahedral and tetrahedral coordination incorporated in MnFe_2_O_4_ according to the obtained Mössbauer parameters (see Table 3) and previously reported studies [64]. In this case, x is close to 1, i.e., the spinel phase is close to MnFe_2_O_4_. The as obtained cation distributions and spinel structures with Fe-rich and Fe-deficient areas were previously reported in similar glass-ceramic materials [13].

It has previously been shown that the preparation conditions of MnFe_2_O_4_ may lead to a shift in the redox ratios of manganese and iron ions, which affects the distribution of Mn and Fe cations in the crystalline structure [67]. In silicate glasses, the dominant diffusion phenomenon is the cationic motion. When during thermal treatment, Mn_y_Fe_1−y_Fe_2_O_4_ particles are formed, which subsequently grow, the glass near the crystal is depleted in Fe and Mn and hence a shell enriched in the other glass components, especially of Si, is formed. This leads to an increase in viscosity in the shell which acts as a diffusion barrier, and decelerates further diffusion of Fe and Mn to the crystal. With increasing crystallization time, this layer gets thicker [13] and the growth process is slowed down. Similar mechanisms have already been observed for the crystallization of fluorides [68] and quartz solid solutions from silicate glasses [69]. The composition of the particles and matrix evaluated after fitting the relative contrast in [13] reveals that with increasing crystallization time, i.e., during growth of the core, Fe is partially replaced by Mn. Using longer annealing times, the composition should approach that of pure jacobsite (MnFe_2_O_4_). In summary, the particles nucleate in a composition close to magnetite and then with proceeding crystallization time, the outer parts of the growing crystals get more and more enriched in Mn. This behavior could be understood if larger diffusion coefficients of Mn^2+^ ions are suggested, which allows them to penetrate the diffusion barrier of the SiO_2_-enriched shell more easily. This also explains the smaller ratio of Fe/Mn in the dendrites if also polygons occur as in the oxidized samples 20F and 25F. In the samples 20FR and 25FR, the changing MnO concentration as well as the presence of the Fe^2+^ ions and their influence on the structure, depending on ferrous ions concentration, should also be taken into account.

## 5. Conclusions

Amorphous samples are obtained for up to 15 mol% Fe_2_O_3_ in the reduced (prepared using iron oxalate) compositions while for the oxidized ones (Fe_2_O_3_ used as raw material) crystallization occurs in the system Na_2_O-MnO-SiO_2_-Fe_2_O_3_ during quenching the melt. The phase composition investigated by X-ray diffraction witnesses the precipitation of monophase Mn_x_Fe_3−x_O_4_ based solid solutions whose crystalline fraction increases with increasing iron oxide concentration. The Raman spectra show that the reduced samples possess compositions in which the solid solutions are enriched in Fe while the spectra of the oxidized specimens approach that of pure jacobsite. The microstructure as studied by scanning electron and optical microscopy, shows the presence of bulk crystallization. Polygon-shaped and dendritic morphological types of crystals are observed for the oxidized samples and only dendrites for the reduced ones. For higher iron oxide concentrations, the average size of the crystals as well as the volume fraction of the crystalline phase increase. Mössbauer spectroscopy shows that the Fe ions are mainly present as Fe^3+^ in tetrahedral coordination in the amorphous matrix and as Fe^3+^ as a part of the solid solution based on Mn_x_Fe_3−x_O_4_. The simultaneous presence of a solid solution of the Mn_x_Fe_3−x_O_4_ type and some minor quantity of MnFe_2_O_4_ is suggested. The room temperature magnetic properties, studied by vibrating sample magnetometer reveal ferrimagnetic behavior for all investigated glass-crystalline materials and increasing saturation magnetization with increasing iron concentration which makes them suitable for electronic and sensor applications.

## Figures and Tables

**Table 1:** Chemical compositions of the prepared samples in mol%.

Sample Name	Na_2_O	MnO	SiO_2_	Fe_2_O_3_
15F/15FR	13.6	8.5	62.9	15
20F/20FR	12.8	8.0	59.2	20
25F/25FR	12.0	7.5	55.5	25

**Table 2:** Cell parameters and average crystallite sizes determined from the data in Figure 1.

Sample Name	Cell Parameter, Å	Crystallite Size, Nm	Amorphous Fraction, wt% (±1)	Crystalline Fraction, wt% (±1)
20F	8.4499 ± 0.0002	35	84	16
20FR	8.4268 ± 0.0006	27	89	11
25F	8.4521 ± 0.0003	38	82	18
25FR	8.4219 ± 0.0007	32	84	16

**Table 3:** Mössbauer parameters of the investigated samples (IS—isomer shift relative to α-Fe at room temperature, QS—quadrupole splitting, FWHM—Lorentzian line width, H_eff_—effective hyperfine magnetic field, G—relative weight) of the different deconvolution components.

Sample Name	Components	IS, mm/s	QS, mm/s	H_eff_, T	FWHM, mm/s	G, %
15F, RT	Db1-Fe^3+^	0.25	0.95	-	0.57	90
	Db2-Fe^2+^	0.89	2.10	-	0.71	10
15F, LNT	Sx-SPM iron oxides	0.44	−0.07	48.5	0.30	5
	Db1-Fe^3+^	0.35	0.99	-	0.55	85
	Db2-Fe^2+^	0.99	2.31	-	0.89	10
15FR	Db1-Fe^3+^	0.25	0.96	-	0.57	82
	Db2-Fe^2+^	0.98	2.00	-	0.53	12
20F	Sx1-Fe^3+^_tetra_-Mn_x_Fe_3−X_O_4_	0.28	0.00	49.0	0.32	16
	Sx2-Fe^2,5+^_octa_-Mn_x_Fe_3−X_O_4_	0.54	−0.04	43.8	0.75	27
	Sx3-Fe^3+^-MnFe_2_O_4_	0.46	−0.03	46.7	0.47	21
	Db1-Fe^3+^_tetra_	0.23	0.92	-	0.53	36
20FR	Sx1-Fe^3+^_tetra_-Mn_x_Fe_3−X_O_4_	0.29	0.00	48.6	0.37	14
	Sx2-Fe^2,5+^_octa_-Mn_x_Fe_3−X_O_4_	0.61	−0.06	42.5	0.98	24
	Sx3-Fe^3+^-MnFe_2_O_4_	0.47	−0.04	46.2	0.57	19
	Db1-Fe^3+^_tetra_	0.24	0.95	-	0.59	36
	Db2-Fe^2+^_octa_	0.93	1.93	-	0.54	7
25F	Sx1-Fe^3+^_tetra_-Mn_x_Fe_3−X_O_4_	0.28	0.01	48.8	0.34	19
	Sx2-Fe^2,5+^_octa_-Mn_x_Fe_3−X_O_4_	0.59	−0.04	43.7	0.45	34
	Sx3-Fe^3+^-MnFe_2_O_4_	0.45	−0.01	46.2	0.44	18
	Db1-Fe^3+^_tetra_	0.23	0.88	-	0.50	29
25FR	Sx1-Fe^3+^_tetra_-Mn_x_Fe_3−X_O_4_	0.28	0.00	48.9	0.34	20
	Sx2-Fe^2,5+^_octa_-Mn_x_Fe_3−X_O_4_	0.64	−0.03	44.1	0.82	33
	Sx3-Fe^3+^-MnFe_2_O_4_	0.48	−0.07	46.9	0.46	16
	Db1-Fe^3+^_tetra_	0.23	0.96	-	0.60	21
	Db2-Fe^2+^_octa_	0.93	1.86	-	0.54	10

**Table 4:** Magnetic characteristics—saturation magnetization, *σ_s_* and coercivity, *H_c_*, obtained for the glass-crystalline samples.

Sample Name	$\sigma_{s}$ (emu/g)	$H_{C}$ (Oe)
20F	19 ± 2	205 ± 5
20FR	13 ± 2	160 ± 5
25F	34 ± 2	39 ± 5
25FR	17 ± 2	210 ± 5

## Data Availability

The original contributions presented in the study are included in the article/Supplementary Material. Further inquiries can be directed to the corresponding author.

## Supplementary Materials

The following supporting information can be downloaded at: https://www.mdpi.com/article/10.3390/ma19091771/s1, Supplementary Material S1. Results from the XRD measurements and Rietveld data refinement for the glass-crystalline samples investigated; Supplementary Material S2. Results from the SEM-EDXS investigations on the glass-crystalline samples.

## Abbreviations

The following abbreviations are used in this manuscript:

XRD	X-ray Diffraction in the Bragg–Brentano Configuration
SEM	Scanning Electron Microscopy
EDXS	Energy Dispersive X-ray Spectroscopy
VSM	Vibrating Sample Magnetometer
QS	Quadrupole splitting
IS	Isomer shift
SAXS	Small angle X-ray scattering
ASAXS	Anomalous small angle X-ray scattering
