# Supplementary material for: Manganese Ferrite Containing Glass-Crystalline Materials—Phase Composition, Microstructure and Magnetic Properties
Source: Materials (Basel). 2026 Apr 27;19(9):1771. doi: 10.3390/ma19091771 (PMC13164864; doi:10.3390/ma19091771)
Supplement: Supplementary file 1 [file materials-19-01771-s001.zip › materials4195677_Supplementary_material1_proffread.pdf]

Materials-4195677 – Supplementary material

Supplementary material S1.

Results from the XRD measurements and Rietveld data refinement for the glass-crystalline samples investigated.

Figure S1. Sample 20F

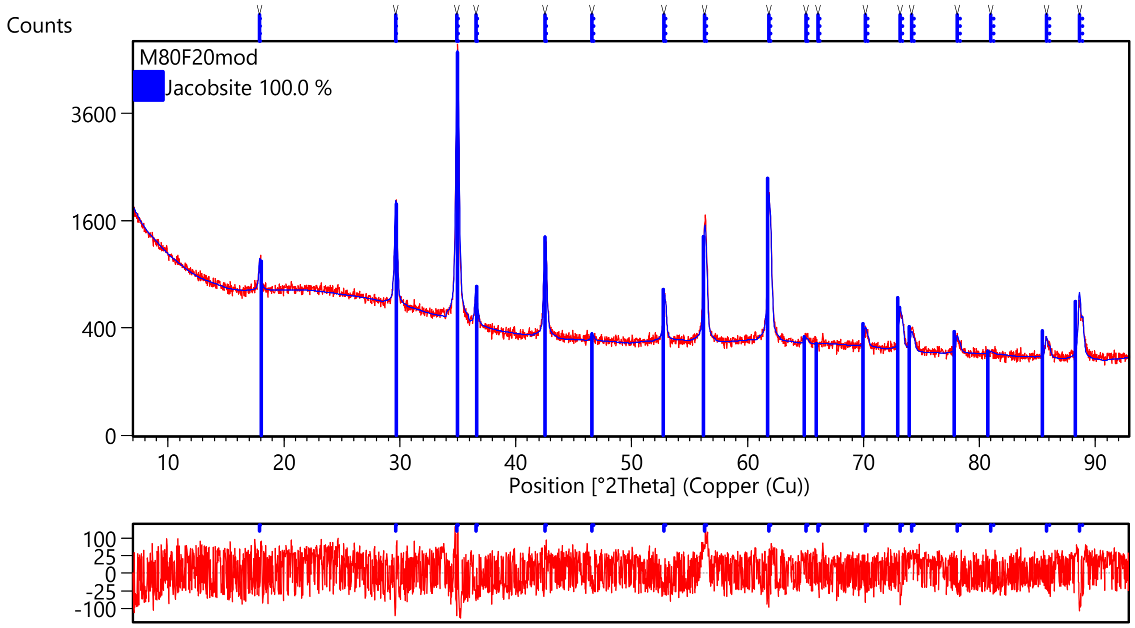

Structures Report: (Bookmark 1)

Global Parameters

Number of used phases	1
Number of variables	4
Number of constraints	1
Zero shift/ °2Theta	-0.248(2)
Specimen displacement/ mm	0.000000
Profile function	Pseudo Voigt
Background	Use available background
R (expected)/ %	4.60708
R (profile)/ %	4.56712
R (weighted profile)/ %	5.89713
GOF	1.63844
d-statistic	1.20229
U standard	0.000000
V standard	0.000000
W standard	0.010000
U Left	0.000000
V Left	0.000000
W Left	0.010000
U Right	0.000000
V Right	0.000000

W Right	0.010000
Asymmetry Type	No Asymmetry Function
Asymmetry 1	0.000000
Asymmetry 2	0.000000
Shape Type	Shape Individual
Shape 1 Left	0.600000
Shape 2 Left	0.000000
Shape 3 Left	0.000000
Shape 1 Right	0.600000
Shape 2 Right	0.000000
Shape 3 Right	0.000000
K a1/a2 intensity ratio	0.500000
K alpha/beta intensity ratio	0.000000
Crystal Shape Factor K	1.0000
Instrumental FWHM Curve Type	Caglioti function
Instr. Gauss Curve Coefficient A	0.0045(5)
Instr. Gauss Curve Coefficient B	-0.0032(9)
Instr. Gauss Curve Coefficient C	0.0046(3)
Instr. Lorentz Curve Coefficient A	0.0062(7)
Instr. Lorentz Curve Coefficient B	-0.004(1)
Instr. Lorentz Curve Coefficient C	0.0064(5)

Relevant parameters of Jacobsite

Structure and profile data	
Formula sum	$O_{32.00}Mn_{8.00}Fe_{16.00}$
Formula mass/ g/mol	1845.0370
Density (calculated)/ g/cm^3	5.0769
F(000)	872.0000
Weight fraction/ %	100.000000
Space group (No.)	F d -3 m (227)
Lattice parameters	
a/ A	8.4499(2)
b/ A	8.4499(2)
c/ A	8.4499(2)
alpha/ °	90
beta/ °	90
gamma/ °	90
V/ 10^6 pm^3	603.38360
Overall displacement parameter	0.000000
Extinction	0.000000
Flat Plate Absorption Correction	0.000000
Porosity	0.000000
Roughness	0.000000
Fitting mode	Structure Fit
U Left	0.000000
V Left	0.000000
W Left	0.0578(8)
Pref. orientation direction/ hkl	0.00 0.00 1.00

Pref. orientation parameter	1.000000
Asymmetry parameter 1	0.000000
Asymmetry parameter 2	0.000000
Peak shape	
parameter 1 Left	0.600000
parameter 2 Left	0.000000
parameter 3 Left	0.000000
R (Bragg)/ %	5.94589

Occupancy, atomic fract. coordinates and Biso for Jacobsite

Atom	Wyck.	s.o.f.	x	y	z	Biso/ 10^4 pm^2
O1	32e	1.000000	0.248000	0.248000	0.248000	0.500000
MN1	16c	0.200000	0.000000	0.000000	0.000000	0.500000
FE1	16c	0.800000	0.000000	0.000000	0.000000	0.500000
FE2	8b	0.400000	0.375000	0.375000	0.375000	0.500000
MN2	8b	0.600000	0.375000	0.375000	0.375000	0.500000

Figure S2. Sample 20FR

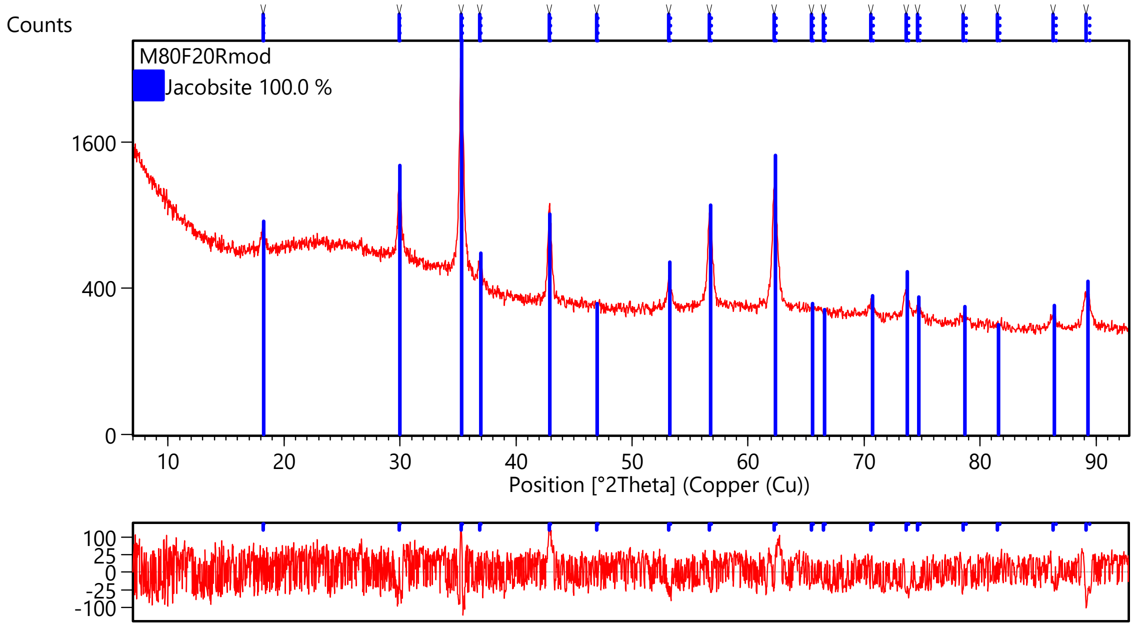

Structures Report: (Bookmark 1)

Global Parameters

Number of used phases	1
Number of variables	4
Number of constraints	1
Zero shift/ °2Theta	-0.026(3)
Specimen displacement/ mm	0.000000
Profile function	Pseudo Voigt
Background	Use available background
R (expected)/ %	5.01416
R (profile)/ %	3.96414
R (weighted profile)/ %	5.28471
GOF	1.11082
d-statistic	0.62421
U standard	0.000000
V standard	0.000000
W standard	0.010000
U Left	0.000000
V Left	0.000000
W Left	0.010000
U Right	0.000000
V Right	0.000000
W Right	0.010000
Asymmetry Type	No Asymmetry Function
Asymmetry 1	0.000000
Asymmetry 2	0.000000
Shape Type	Shape Individual
Shape 1 Left	0.600000
Shape 2 Left	0.000000
Shape 3 Left	0.000000
Shape 1 Right	0.600000
Shape 2 Right	0.000000
Shape 3 Right	0.000000
K a1/a2 intensity ratio	0.500000
K alpha/beta intensity ratio	0.000000
Crystal Shape Factor K	1.0000
Instrumental FWHM Curve Type	Caglioti function
Instr. Gauss Curve Coefficient A	0.0045(5)
Instr. Gauss Curve Coefficient B	-0.0032(9)
Instr. Gauss Curve Coefficient C	0.0046(3)
Instr. Lorentz Curve Coefficient A	0.0062(7)
Instr. Lorentz Curve Coefficient B	-0.004(1)
Instr. Lorentz Curve Coefficient C	0.0064(5)

Relevant parameters of Jacobsite

Structure and profile data	
Formula sum	$O_{32.00}Fe_{16.00}Mn_{8.00}$
Formula mass/ g/mol	1845.0370
Density (calculated)/ g/cm^3	5.1174
F(000)	872.0000

Weight fraction/ %	100.000000
Space group (No.)	F d -3 m (227)
Lattice parameters	
a/ A	8.4268(6)
b/ A	8.4268(6)
c/ A	8.4268(6)
alpha/ °	90
beta/ °	90
gamma/ °	90
V/ 10^6 pm^3	598.61410
Overall displacement parameter	0.000000
Extinction	0.000000
Flat Plate Absorption Correction	0.000000
Porosity	0.000000
Roughness	0.000000
Fitting mode	Structure Fit
U Left	0.000000
V Left	0.000000
W Left	0.119(2)
Pref. orientation direction/ hkl	0.00 0.00 1.00
Pref. orientation parameter	1.000000
Asymmetry parameter 1	0.000000
Asymmetry parameter 2	0.000000
Peak shape	
parameter 1 Left	0.600000
parameter 2 Left	0.000000
parameter 3 Left	0.000000
R (Bragg)/ %	7.60446

Occupancy, atomic fract. coordinates and Biso for Jacobsite

Atom	Wyck.	s.o.f.	x	y	z	Biso/ 10^4 pm^2
O1	32e	1.000000	0.245000	0.245000	0.245000	0.500000
FE1	8b	1.000000	0.375000	0.375000	0.375000	0.500000
MN1	16c	0.500000	0.000000	0.000000	0.000000	0.500000
FE2	16c	0.500000	0.000000	0.000000	0.000000	0.500000

Figure S3. Sample 25F

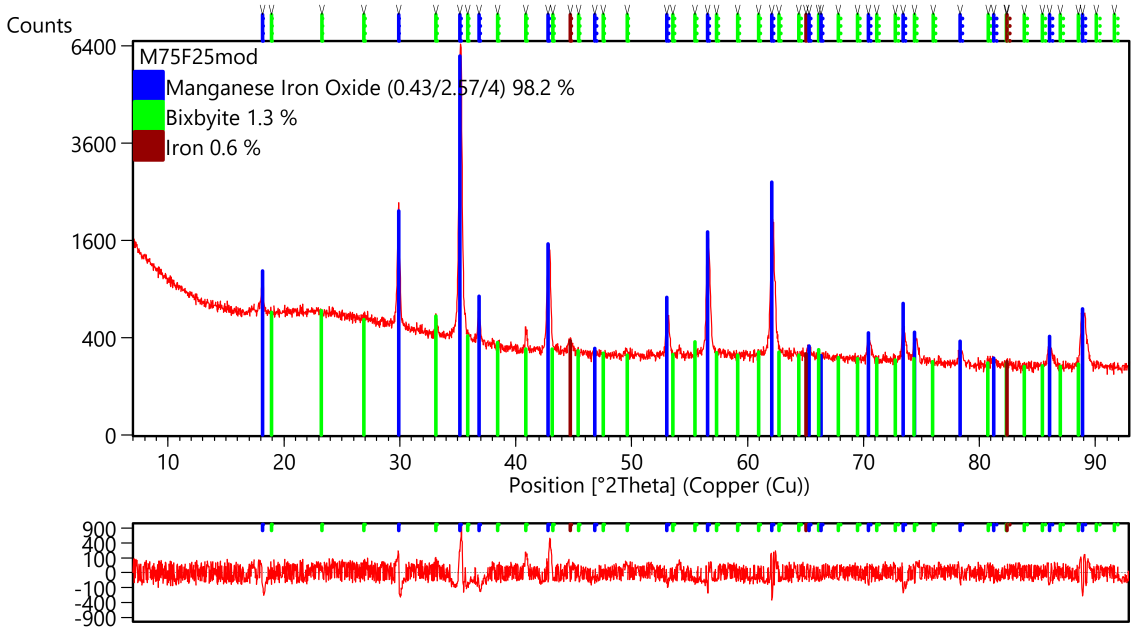

Structures Report: (Bookmark 1)

Global Parameters

Number of used phases	3
Number of variables	6
Number of constraints	1
Zero shift/ °2Theta	0.015(2)
Specimen displacement/ mm	0.000000
Profile function	Pseudo Voigt
Background	Use available background
R (expected)/ %	4.95229
R (profile)/ %	7.47406
R (weighted profile)/ %	9.99773
GOF	4.07559
d-statistic	0.38068
U standard	0.000000
V standard	0.000000
W standard	0.010000
U Left	0.000000
V Left	0.000000
W Left	0.010000
U Right	0.000000
V Right	0.000000
W Right	0.010000
Asymmetry Type	No Asymmetry Function
Asymmetry 1	0.000000
Asymmetry 2	0.000000
Shape Type	Shape Individual

Shape 1 Left	0.600000
Shape 2 Left	0.000000
Shape 3 Left	0.000000
Shape 1 Right	0.600000
Shape 2 Right	0.000000
Shape 3 Right	0.000000
K a1/a2 intensity ratio	0.500000
K alpha/beta intensity ratio	0.000000
Crystal Shape Factor K	1.0000
Instrumental FWHM Curve Type	Caglioti function
Instr. Gauss Curve Coefficient A	0.0045(5)
Instr. Gauss Curve Coefficient B	-0.0032(9)
Instr. Gauss Curve Coefficient C	0.0046(3)
Instr. Lorentz Curve Coefficient A	0.0062(7)
Instr. Lorentz Curve Coefficient B	-0.004(1)
Instr. Lorentz Curve Coefficient C	0.0064(5)

Relevant parameters of Manganese Iron Oxide (0.43/2.57/4)

Structure and profile data	
Formula sum	$O_{32.00}Fe_{20.56}Mn_{3.44}$
Formula mass/ g/mol	1849.1820
Density (calculated)/ g/cm^3	5.0865
F(000)	876.5600
Weight fraction/ %	98(1)
Space group (No.)	F d -3 m (227)
Lattice parameters	
a/ A	8.4521(3)
b/ A	8.4521(3)
c/ A	8.4521(3)
alpha/ °	90
beta/ °	90
gamma/ °	90
V/ 10^6 pm^3	603.59400
Overall displacement parameter	0.000000
Extinction	0.000000
Flat Plate Absorption Correction	0.000000
Porosity	0.000000
Roughness	0.000000
Fitting mode	Structure Fit
U Left	0.000000
V Left	0.000000
W Left	0.0445(9)
Pref. orientation direction/ hkl	0.00 0.00 1.00
Pref. orientation parameter	1.000000
Asymmetry parameter 1	0.000000
Asymmetry parameter 2	0.000000
Peak shape	
parameter 1 Left	0.600000

parameter 2 Left	0.000000
parameter 3 Left	0.000000
R (Bragg)/ %	9.70867

Occupancy, atomic fract. coordinates and Biso for Manganese Iron Oxide (0.43/2.57/4)

Atom	Wyck.	s.o.f.	x	y	z	Biso/ 10^4 pm^2
O1	32e	1.000000	0.244000	0.244000	0.244000	0.500000
FE1	16c	0.785000	0.000000	0.000000	0.000000	0.500000
MN1	16c	0.215000	0.000000	0.000000	0.000000	0.500000
FE2	8b	1.000000	0.375000	0.375000	0.375000	0.500000

Relevant parameters of Bixbyite

Structure and profile data	
Formula sum	$O_{48.00}Mn_{16.00}Fe_{16.00}$
Formula mass/ g/mol	2540.5310
Density (calculated)/ g/cm^3	5.1356
F(000)	1200.0000
Weight fraction/ %	1.3(2)
Space group (No.)	I a -3 (206)
Lattice parameters	
a/ A	9.365000
b/ A	9.365000
c/ A	9.365000
alpha/ °	90
beta/ °	90
gamma/ °	90
V/ 10^6 pm^3	821.34060
Overall displacement parameter	0.000000
Extinction	0.000000
Flat Plate Absorption Correction	0.000000
Porosity	0.000000
Roughness	0.000000
Fitting mode	Structure Fit
U Left	0.000000
V Left	0.000000
W Left	0.010000
Pref. orientation direction/ hkl	0.00 0.00 1.00
Pref. orientation parameter	1.000000
Asymmetry parameter 1	0.000000
Asymmetry parameter 2	0.000000
Peak shape	
parameter 1 Left	0.600000
parameter 2 Left	0.000000

parameter 3 Left 0.000000
R (Bragg)/ % 125.38620

Occupancy, atomic fract. coordinates and Biso for Bixbyite

Atom	Wyck.	s.o.f.	x	y	z	Biso/ 10^4 pm^2
O1	48e	1.000000	0.105000	0.365000	0.130000	0.500000
MN1	24d	0.500000	0.280000	0.000000	0.250000	0.500000
FE1	24d	0.500000	0.280000	0.000000	0.250000	0.500000
MN2	8a	0.500000	0.000000	0.000000	0.000000	0.500000
FE2	8a	0.500000	0.000000	0.000000	0.000000	0.500000

Relevant parameters of Iron

Structure and profile data
Formula sum $Fe_{2.00}$
Formula mass/ g/mol 111.6940
Density (calculated)/ g/cm^3 7.8775
F(000) 52.0000
Weight fraction/ % 0.55(9)
Space group (No.) I m -3 m (229)
Lattice parameters
a/ A 2.866000
b/ A 2.866000
c/ A 2.866000
alpha/ ° 90
beta/ ° 90
gamma/ ° 90
V/ 10^6 pm^3 23.54120
Overall displacement parameter 0.000000
Extinction 0.000000
Flat Plate Absorption Correction 0.000000
Porosity 0.000000
Roughness 0.000000
Fitting mode Structure Fit
U Left 0.000000
V Left 0.000000
W Left 0.010000
Pref. orientation direction/ hkl 0.00 0.00 1.00
Pref. orientation parameter 1.000000
Asymmetry parameter 1 0.000000
Asymmetry parameter 2 0.000000
Peak shape
parameter 1 Left 0.600000

parameter 2 Left	0.000000
parameter 3 Left	0.000000
R (Bragg)/ %	36.83474

Occupancy, atomic fract. coordinates and Biso for Iron

Atom	Wyck.	s.o.f.	x	y	z	Biso/ 10^4 pm^2
FE1	2a	1.000000	0.000000	0.000000	0.000000	0.500000

Figure S4. Sample 25FR

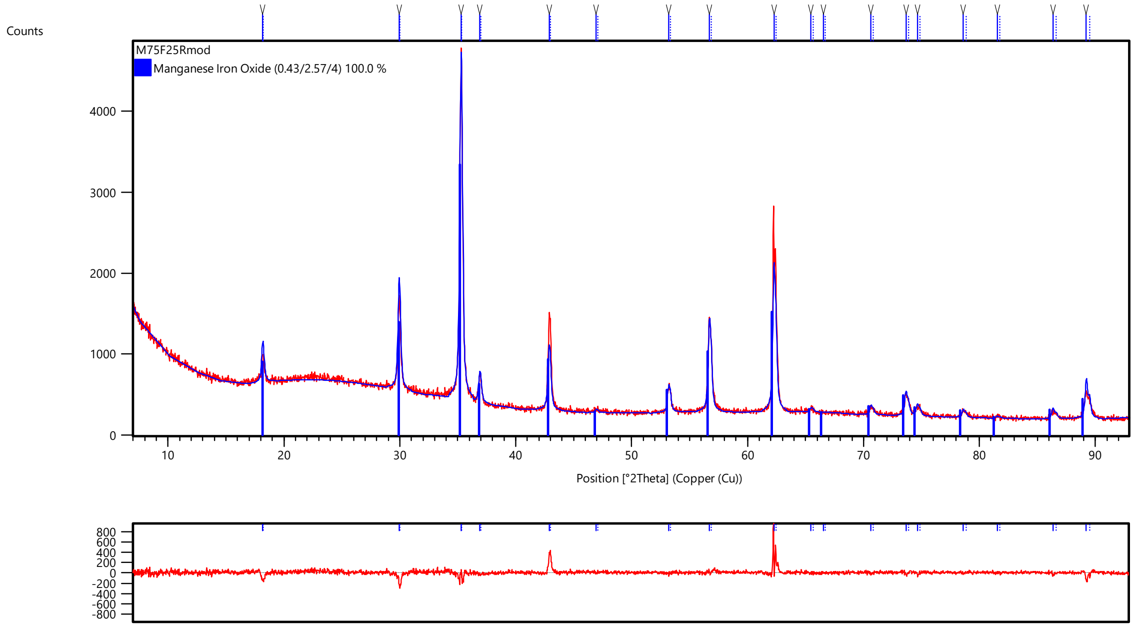

Structures Report: (Bookmark 1)

Global Parameters

Number of used phases	1
Number of variables	4
Number of constraints	1
Zero shift/ °2Theta	-0.054(3)
Specimen displacement/ mm	0.000000
Profile function	Pseudo Voigt
Background	Use available background
R (expected)/ %	4.68618
R (profile)/ %	6.72108
R (weighted profile)/ %	9.29855
GOF	3.93725
d-statistic	0.37869
U standard	0.000000
V standard	0.000000

W standard	0.010000
U Left	0.000000
V Left	0.000000
W Left	0.010000
U Right	0.000000
V Right	0.000000
W Right	0.010000
Asymmetry Type	No Asymmetry Function
Asymmetry 1	0.000000
Asymmetry 2	0.000000
Shape Type	Shape Individual
Shape 1 Left	0.600000
Shape 2 Left	0.000000
Shape 3 Left	0.000000
Shape 1 Right	0.600000
Shape 2 Right	0.000000
Shape 3 Right	0.000000
K a1/a2 intensity ratio	0.500000
K alpha/beta intensity ratio	0.000000
Crystal Shape Factor K	1.0000
Instrumental FWHM Curve Type	Caglioti function
Instr. Gauss Curve Coefficient A	0.0045(5)
Instr. Gauss Curve Coefficient B	-0.0032(9)
Instr. Gauss Curve Coefficient C	0.0046(3)
Instr. Lorentz Curve Coefficient A	0.0062(7)
Instr. Lorentz Curve Coefficient B	-0.004(1)
Instr. Lorentz Curve Coefficient C	0.0064(5)

Relevant parameters of Manganese Iron Oxide (0.43/2.57/4)

Structure and profile data	
Formula sum	$O_{32.00}Fe_{21.13}Mn_{3.93}$
Formula mass/ g/mol	1908.2650
Density (calculated)/ g/cm^3	5.3063
F(000)	903.7816
Weight fraction/ %	100.000000
Space group (No.)	F d -3 m (227)
Lattice parameters	
a/ A	8.4219(7)
b/ A	8.4219(7)
c/ A	8.4219(7)
alpha/ °	90
beta/ °	90
gamma/ °	90
V/ 10^6 pm^3	597.07770
Overall displacement parameter	0.000000
Extinction	0.000000
Flat Plate Absorption Correction	0.000000
Porosity	0.000000

Roughness	0.000000
Fitting mode	Structure Fit
U Left	0.000000
V Left	0.000000
W Left	0.065(1)
Pref. orientation direction/ hkl	0.00 4.00 4.00
Pref. orientation parameter	0.884942
Asymmetry parameter 1	0.000000
Asymmetry parameter 2	0.000000
Peak shape	
parameter 1 Left	0.752674
parameter 2 Left	0.000000
parameter 3 Left	0.000000
R (Bragg)/ %	11.83732

Occupancy, atomic fract. coordinates and Biso for Manganese Iron Oxide (0.43/2.57/4)

Atom	Wyck.	s.o.f.	x	y	z	Biso/ 10^4 pm^2
O1	32e	1.000000	0.244000	0.244000	0.244000	0.500000
FE1	16c	0.820735	0.000000	0.000000	0.000000	0.500000
MN1	16c	0.245890	0.000000	0.000000	0.000000	0.500000
FE2	8b	1.000000	0.375000	0.375000	0.375000	0.500000
