# Supplementary material for: Manganese Ferrite Containing Glass-Crystalline Materials—Phase Composition, Microstructure and Magnetic Properties
Source: Materials (Basel). 2026 Apr 27;19(9):1771. doi: 10.3390/ma19091771 (PMC13164864; doi:10.3390/ma19091771)
Supplement: Supplementary file 1 [file materials-19-01771-s001.zip › materials4195677_Supplementary_material2_proofread.pdf]

Materials-4195677 – Supplementary material

Supplementary material S2.

Results from the SEM-EDXS investigations on the glass-crystalline samples.

Sample 20F

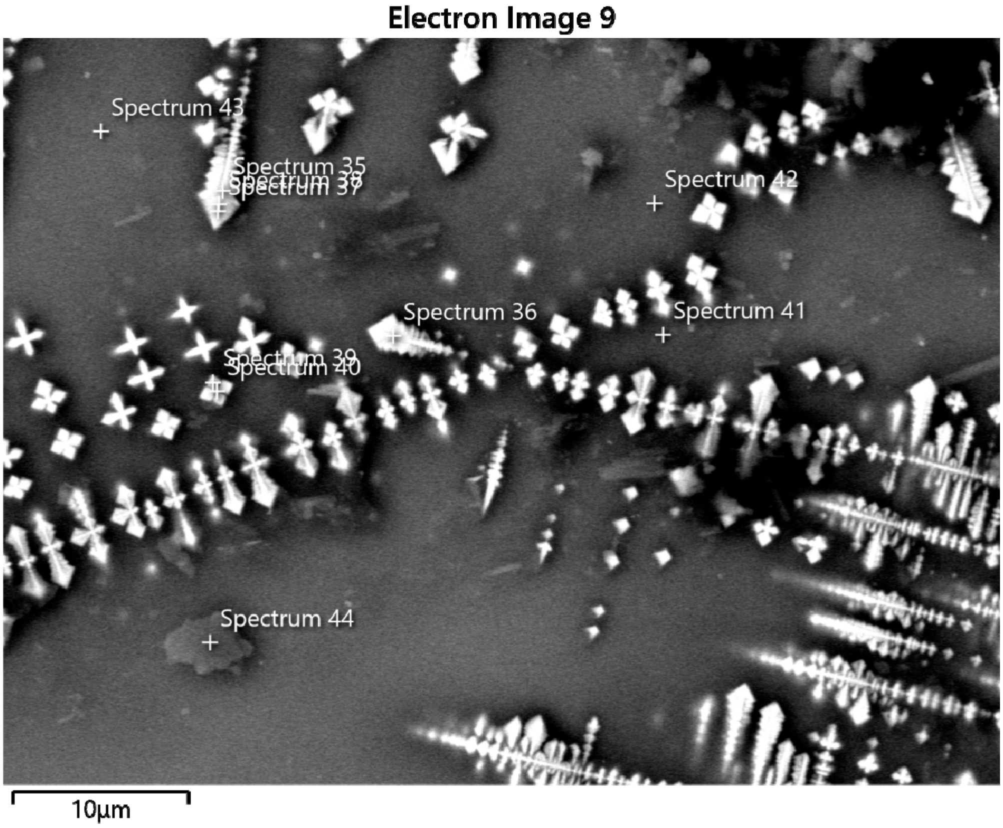

Figure S1

Table S1

Element	35	36	37	38	39	40	41	42	43
Mn	7,1	6,87	7,07	7,32	7,07	7,18	2,79	2,96	3,05
Fe	31,24	30,87	31,79	31,23	30,91	32,39	14,68	16,69	18,07
Ratio Fe/Mn	**4,4**	**4,5**	**4,5**	**4,3**	**4,4**	**4,5**	*5,3*	*5,6*	*5,9*
	**Dendrites**	dendrites	dendrites	dendrites	dendrites	dendrites	*Glass*	*Glass*	*Glass*

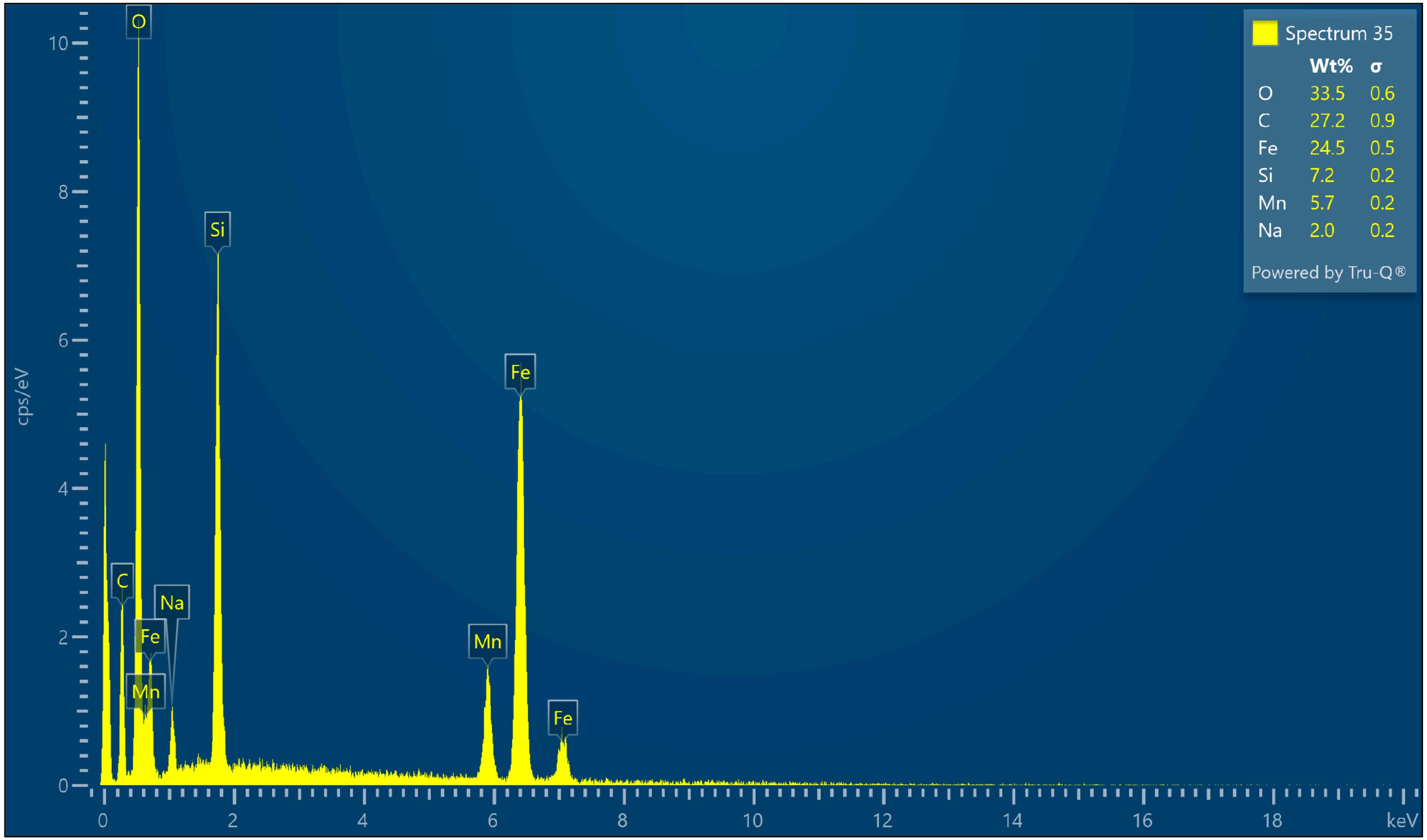
10
8
6
4
2
0
cps/eV
O
Si
Fe
C
Na
Fe
Mn
Mn
Fe
0
2
4
6
8
10
12
14
16
18
keV
Spectrum 35
Wt% σ
O 33.5 0.6
C 27.2 0.9
Fe 24.5 0.5
Si 7.2 0.2
Mn 5.7 0.2
Na 2.0 0.2
Powered by Tru-Q®

Figure S2

Table S2. Spectrum 35									
Element	Signal Type	Line	Apparent Concentration	k Ratio	Wt%	Wt% Sigma	Standard Name	Factory Standard	Standardization Date
C	EDS	K series	9.72	0.09722	27.18	0.88	C Vit	Yes	
O	EDS	K series	44.93	0.15120	33.45	0.61	SiO2	Yes	
Na	EDS	K series	1.81	0.00762	1.98	0.15	Albite	Yes	
Si	EDS	K series	8.72	0.06907	7.20	0.17	SiO2	Yes	
Mn	EDS	K series	7.10	0.07098	5.67	0.22	Mn	Yes	
Fe	EDS	K series	31.24	0.31236	24.52	0.46	Fe	Yes	
Total					100.00				

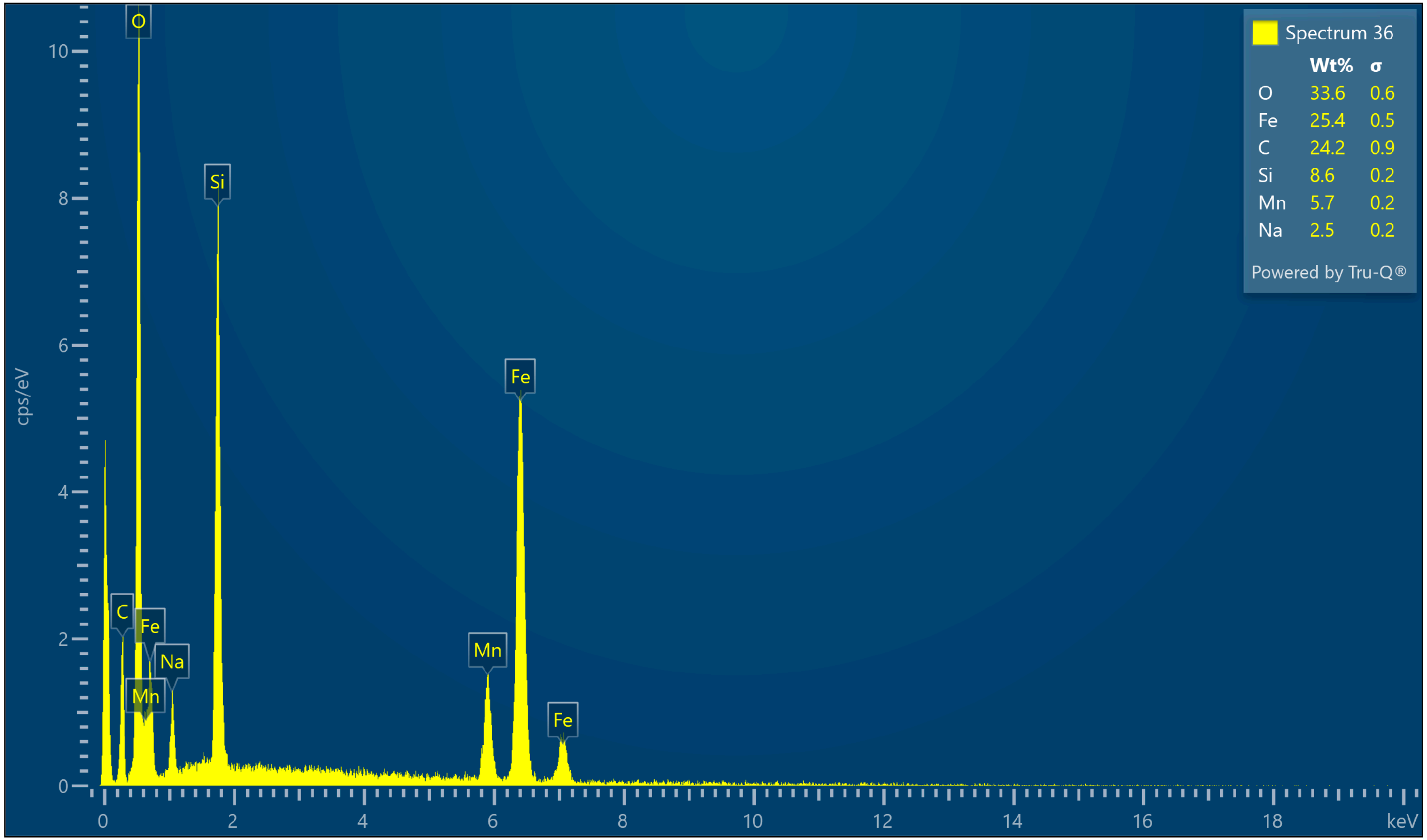
Spectrum 36
Wt% σ
O 33.6 0.6
Fe 25.4 0.5
C 24.2 0.9
Si 8.6 0.2
Mn 5.7 0.2
Na 2.5 0.2
Powered by Tru-Q®
cps/eV
keV
O
Si
Fe
C
Fe
Na
Mn
Mn
Fe
0
2
4
6
8
10
12
14
16
18

Figure S3

Table S3. Spectrum 36									
Element	Signal Type	Line	Apparent Concentration	k Ratio	Wt%	Wt% Sigma	Standard Name	Factory Standard	Standardization Date
C	EDS	K series	7.65	0.07654	24.19	0.91	C Vit	Yes	
O	EDS	K series	44.82	0.15082	33.60	0.62	SiO2	Yes	
Na	EDS	K series	2.18	0.00922	2.53	0.17	Albite	Yes	
Si	EDS	K series	9.79	0.07759	8.56	0.20	SiO2	Yes	
Mn	EDS	K series	6.87	0.06866	5.74	0.22	Mn	Yes	
Fe	EDS	K series	30.87	0.30866	25.37	0.48	Fe	Yes	
Total					100.00				

OXFORD INSTRUMENTS

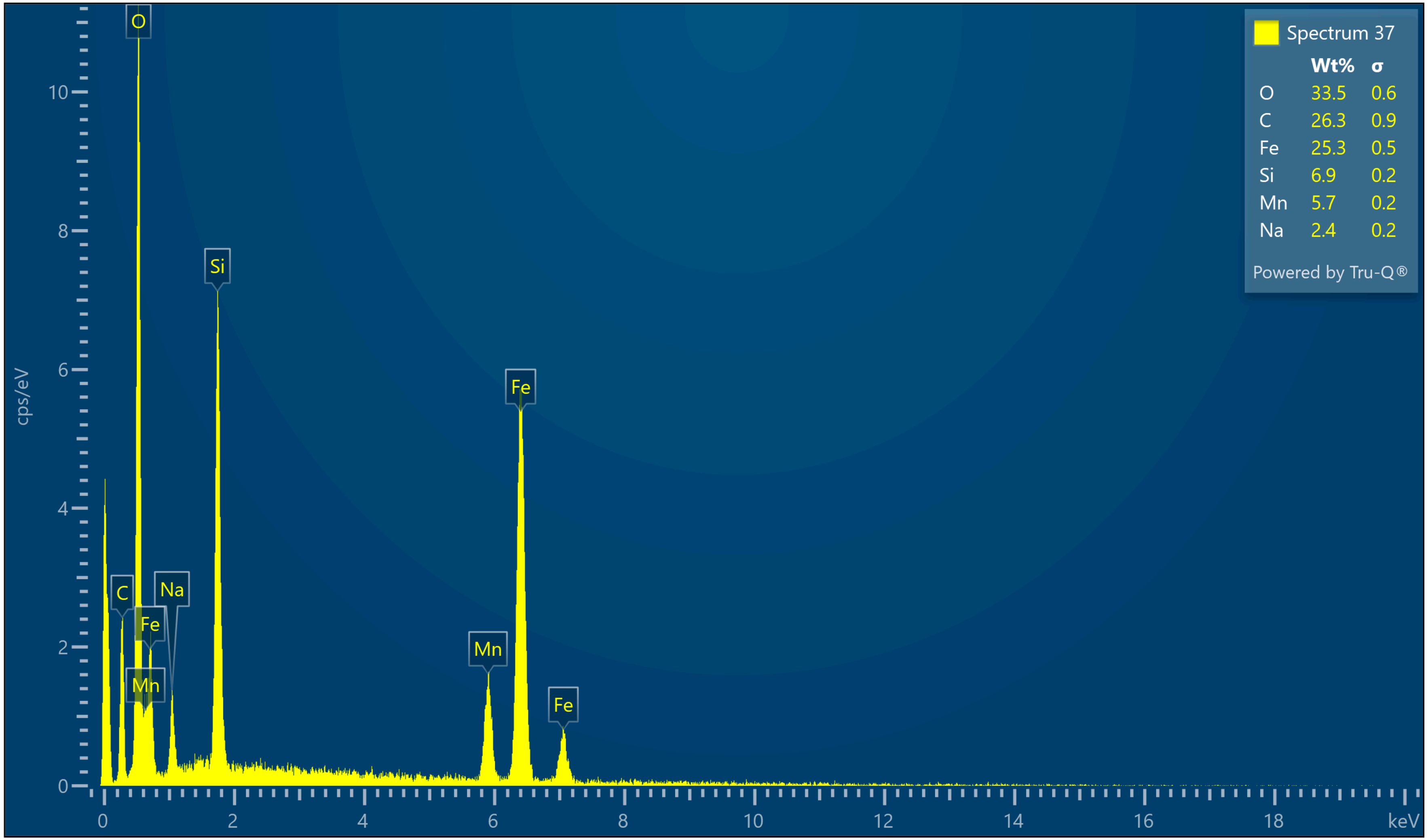

Figure S4

OXFORD INSTRUMENTS

Table S4. Spectrum 37									
Element	Signal Type	Line	Apparent Concentration	k Ratio	Wt%	Wt% Sigma	Standard Name	Factory Standard	Standardization Date
C	EDS	K series	9.23	0.09228	26.26	0.89	C Vit	Yes	
O	EDS	K series	45.25	0.15225	33.47	0.61	SiO2	Yes	
Na	EDS	K series	2.12	0.00895	2.39	0.17	Albite	Yes	
Si	EDS	K series	8.10	0.06422	6.86	0.17	SiO2	Yes	
Mn	EDS	K series	7.07	0.07066	5.72	0.22	Mn	Yes	
Fe	EDS	K series	31.79	0.31793	25.30	0.47	Fe	Yes	
Total					100.00				

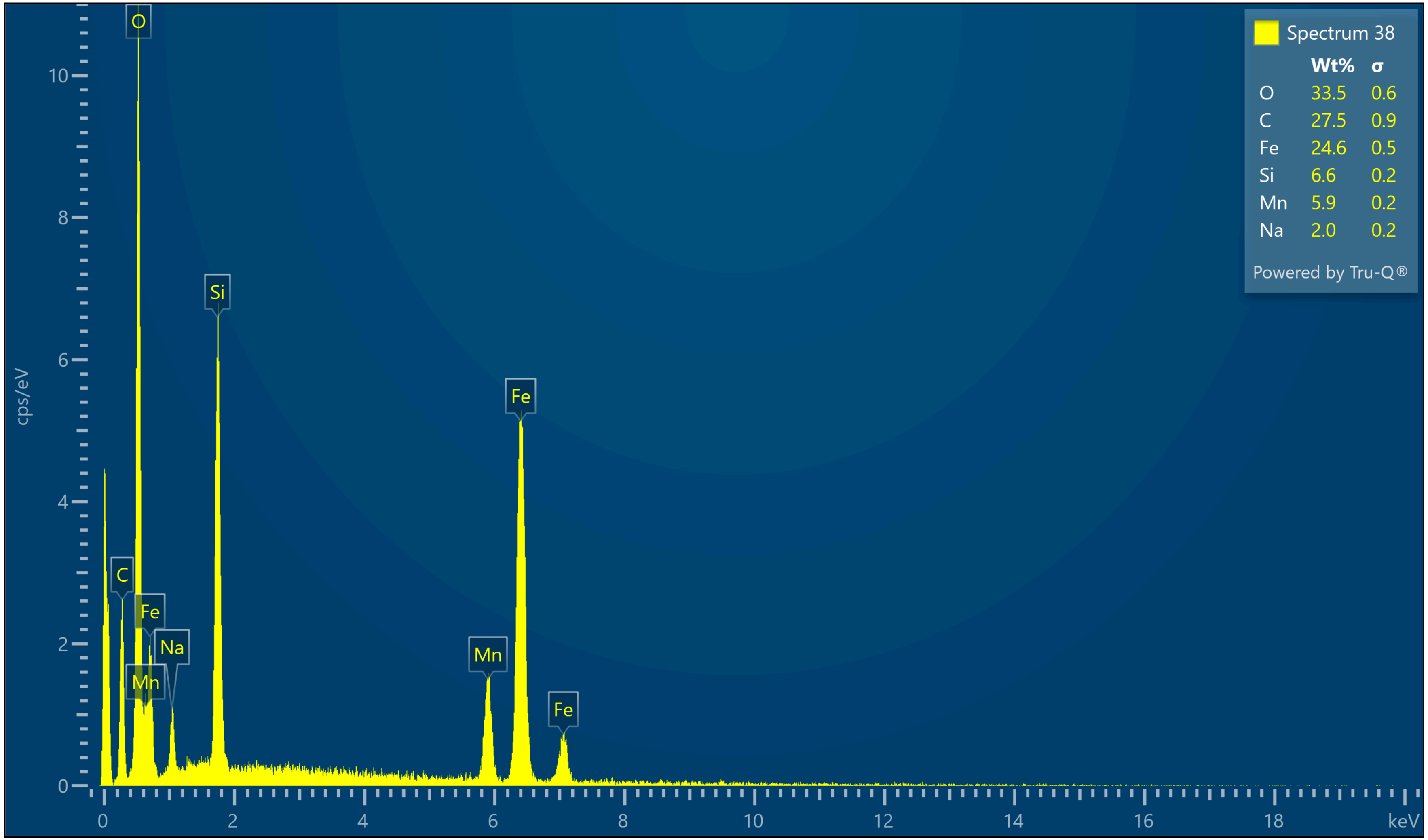
Spectrum 38
Wt% σ
O 33.5 0.6
C 27.5 0.9
Fe 24.6 0.5
Si 6.6 0.2
Mn 5.9 0.2
Na 2.0 0.2
Powered by Tru-Q®
cps/eV
keV
O
Si
Fe
C
Fe
Na
Mn
Mn
Fe

Figure S5

Table S5. Spectrum 38									
Element	Signal Type	Line	Apparent Concentration	k Ratio	Wt%	Wt% Sigma	Standard Name	Factory Standard	Standardization Date
C	EDS	K series	10.00	0.09997	27.47	0.88	C Vit	Yes	
O	EDS	K series	44.88	0.15101	33.48	0.62	SiO2	Yes	
Na	EDS	K series	1.77	0.00748	1.96	0.16	Albite	Yes	
Si	EDS	K series	7.99	0.06331	6.64	0.17	SiO2	Yes	
Mn	EDS	K series	7.32	0.07319	5.86	0.22	Mn	Yes	
Fe	EDS	K series	31.23	0.31230	24.59	0.46	Fe	Yes	
Total					100.00				

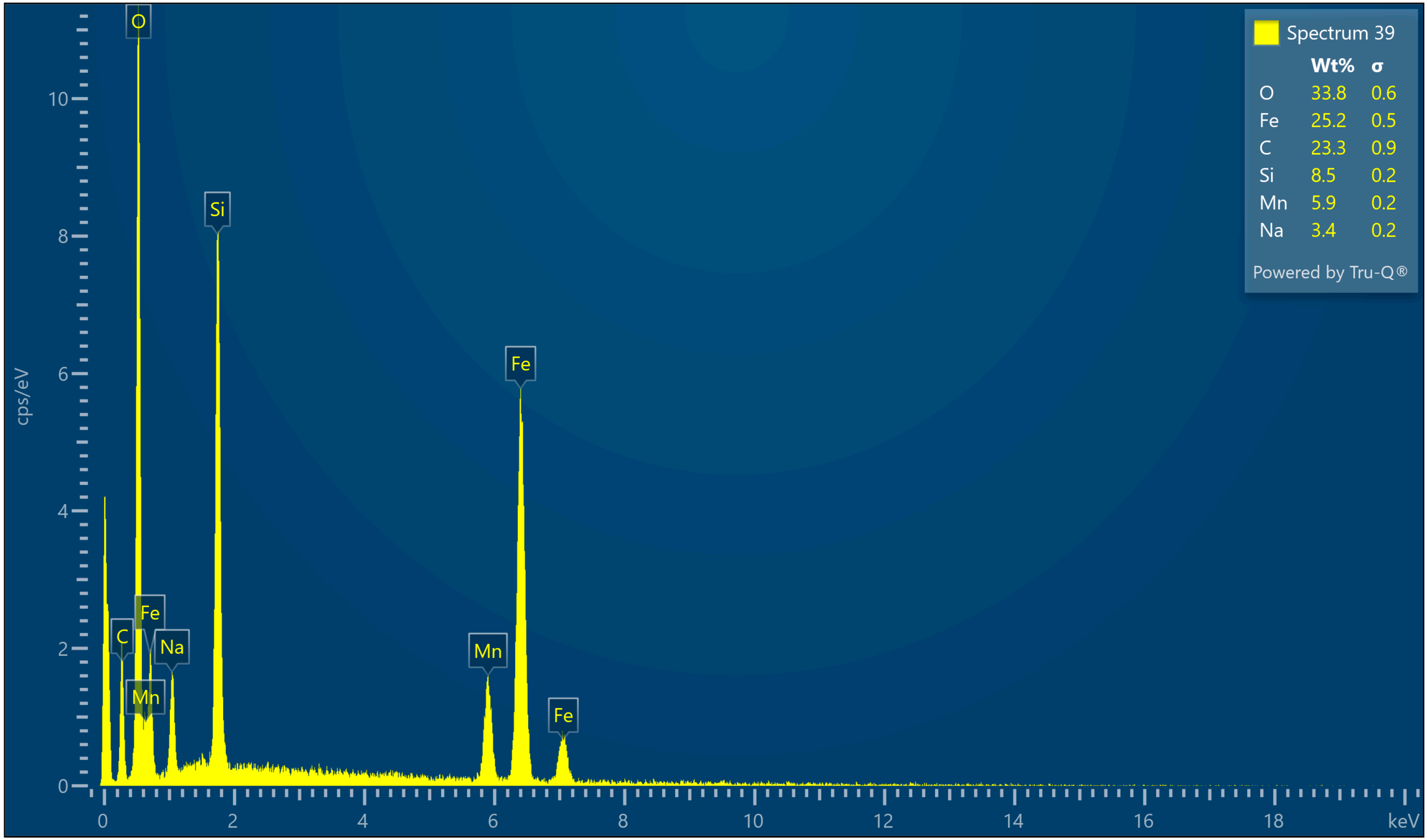
Spectrum 39
Wt% σ
O 33.8 0.6
Fe 25.2 0.5
C 23.3 0.9
Si 8.5 0.2
Mn 5.9 0.2
Na 3.4 0.2
Powered by Tru-Q®
cps/eV
keV
O
Si
Fe
C
Na
Mn
0
2
4
6
8
10
12
14
16
18

Figure S6

Table S6. Spectrum 39									
Element	Signal Type	Line	Apparent Concentration	k Ratio	Wt%	Wt% Sigma	Standard Name	Factory Standard	Standardization Date
C	EDS	K series	7.38	0.07382	23.32	0.92	C Vit	Yes	
O	EDS	K series	46.40	0.15614	33.84	0.61	SiO2	Yes	
Na	EDS	K series	2.95	0.01243	3.37	0.19	Albite	Yes	
Si	EDS	K series	9.72	0.07701	8.46	0.20	SiO2	Yes	
Mn	EDS	K series	7.07	0.07074	5.86	0.22	Mn	Yes	
Fe	EDS	K series	30.91	0.30912	25.15	0.47	Fe	Yes	
Total					100.00				

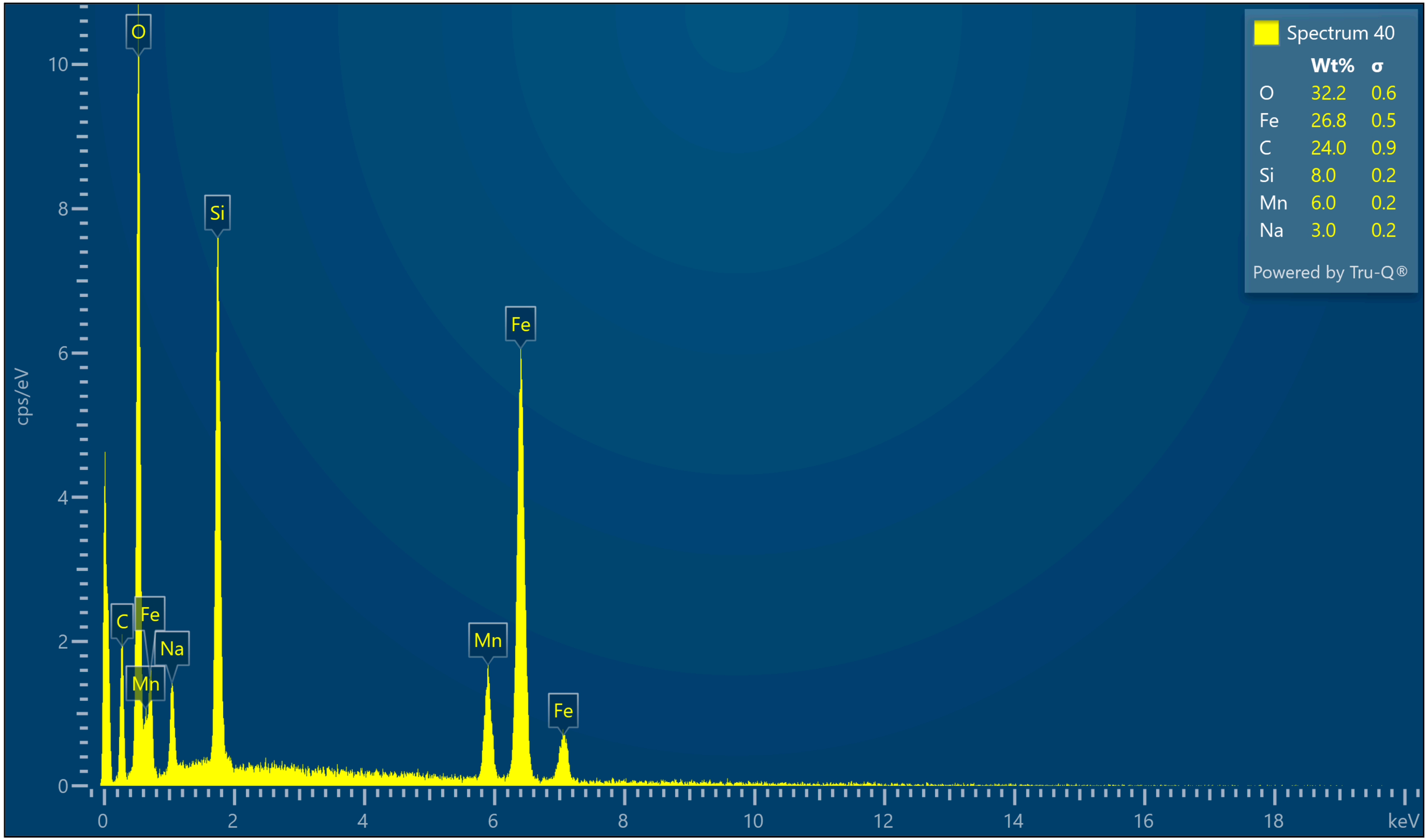
Spectrum 40
Wt% σ
O 32.2 0.6
Fe 26.8 0.5
C 24.0 0.9
Si 8.0 0.2
Mn 6.0 0.2
Na 3.0 0.2
Powered by Tru-Q®
cps/eV
keV
O
Si
Fe
C
Fe
Na
Mn
Mn
Fe
0
2
4
6
8
10
12
14
16
18

Figure S7

Table S7. Spectrum 40

Element	Signal Type	Line	Apparent Concentration	k Ratio	Wt%	Wt% Sigma	Standard Name	Factory Standard	Standardization Date
C	EDS	K series	7.54	0.07544	24.00	0.94	C Vit	Yes	
O	EDS	K series	42.77	0.14394	32.17	0.61	SiO2	Yes	
Na	EDS	K series	2.50	0.01055	2.96	0.19	Albite	Yes	
Si	EDS	K series	9.02	0.07146	8.03	0.19	SiO2	Yes	
Mn	EDS	K series	7.18	0.07184	6.05	0.23	Mn	Yes	
Fe	EDS	K series	32.39	0.32388	26.78	0.50	Fe	Yes	
Total					100.00				

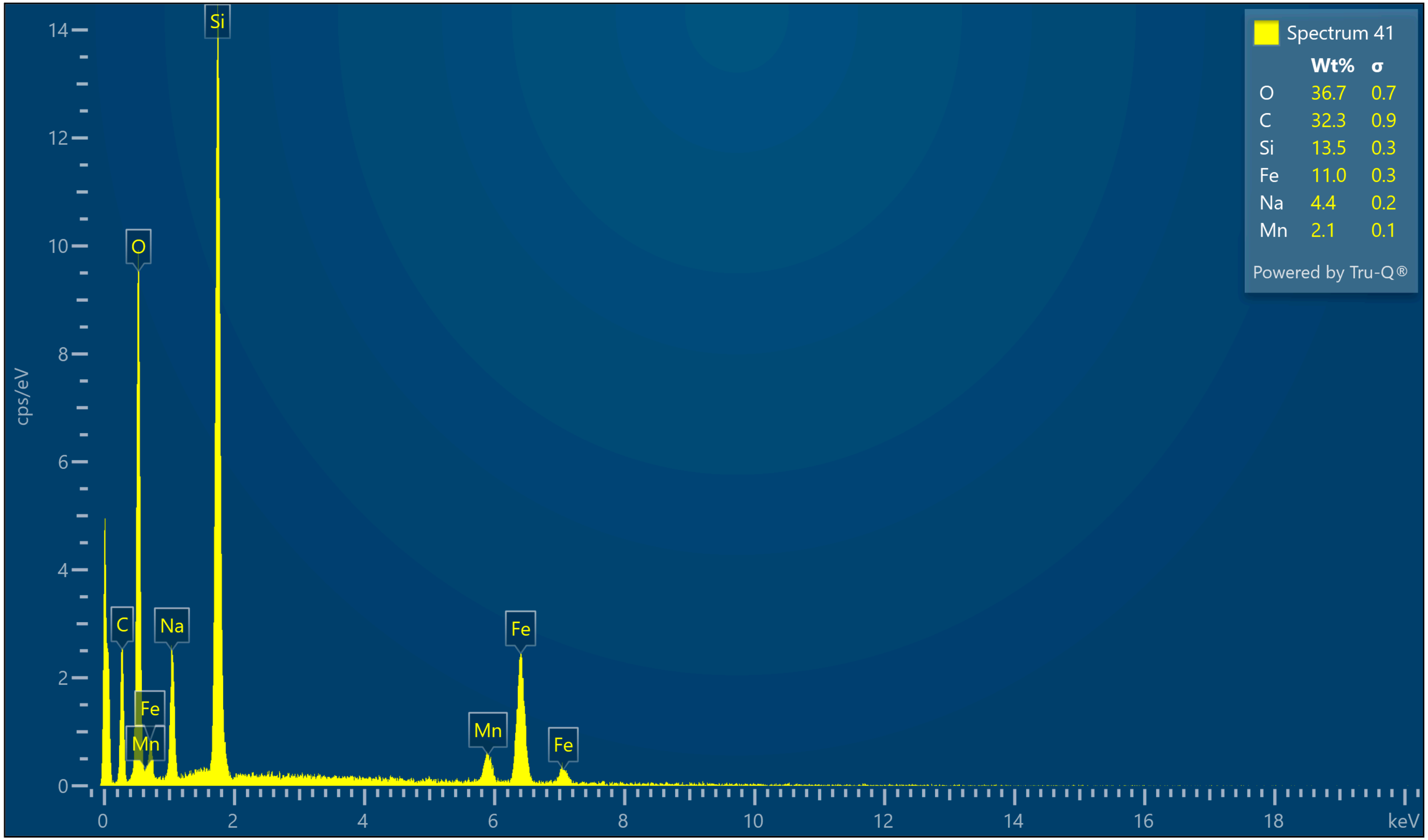
Spectrum 41
Wt% σ
O 36.7 0.7
C 32.3 0.9
Si 13.5 0.3
Fe 11.0 0.3
Na 4.4 0.2
Mn 2.1 0.1
Powered by Tru-Q®
cps/eV
keV
Si
O
C
Na
Fe
Mn
Mn
Fe
Fe

Figure S8

**Table S8. Spectrum 41**									
Element	Signal Type	Line	Apparent Concentration	k Ratio	Wt%	Wt% Sigma	Standard Name	Factory Standard	Standardization Date
C	EDS	K series	10.96	0.10956	32.33	0.89	C Vit	Yes	
O	EDS	K series	44.54	0.14990	36.69	0.65	SiO2	Yes	
Na	EDS	K series	5.43	0.02293	4.37	0.17	Albite	Yes	
Si	EDS	K series	19.21	0.15219	13.51	0.26	SiO2	Yes	
Mn	EDS	K series	2.79	0.02795	2.13	0.15	Mn	Yes	
Fe	EDS	K series	14.68	0.14676	10.97	0.30	Fe	Yes	
Total					100.00				

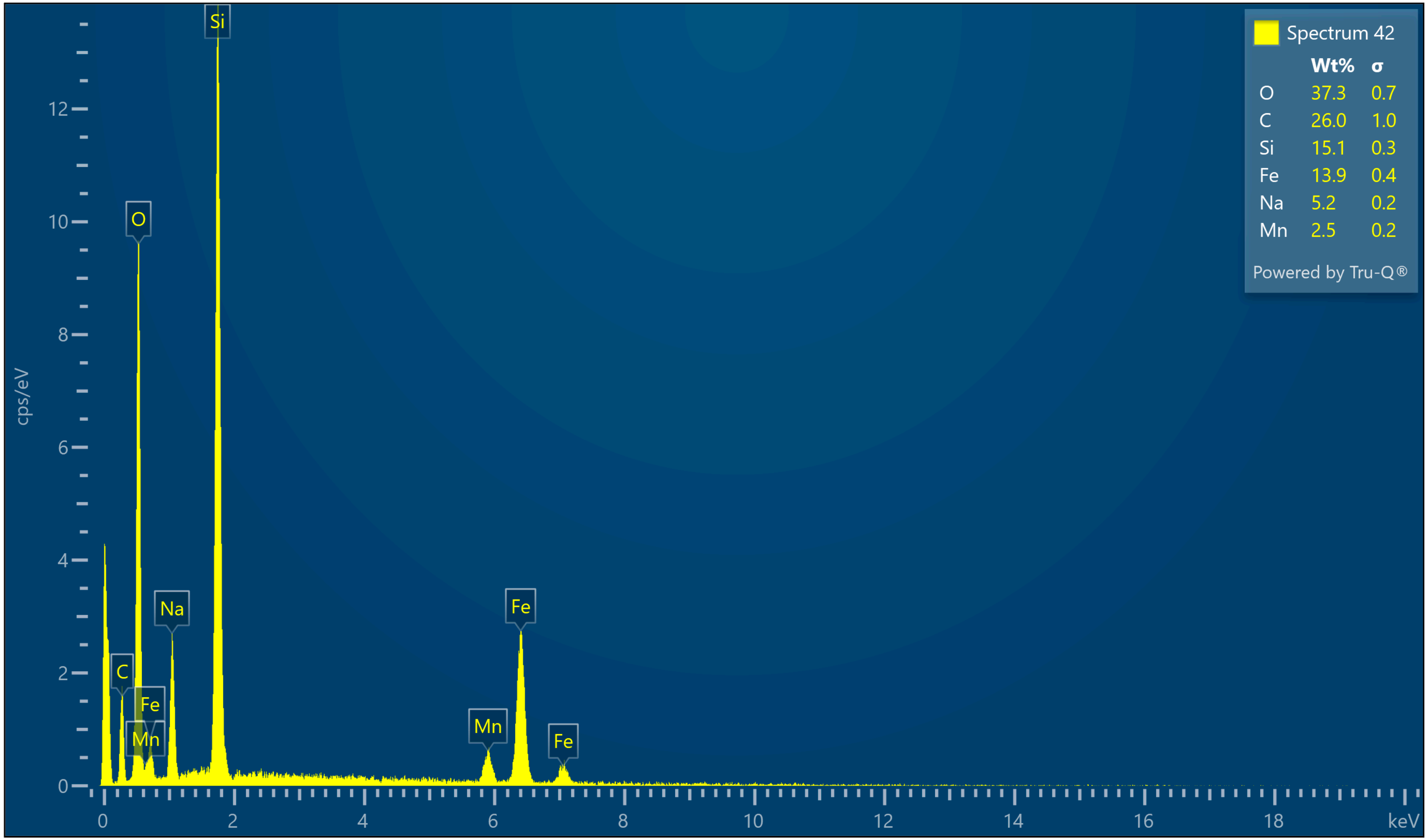

Figure S9

OXFORD INSTRUMENTS

Table S9. Spectrum 42									
Element	Signal Type	Line	Apparent Concentration	k Ratio	Wt%	Wt% Sigma	Standard Name	Factory Standard	Standardization Date
C	EDS	K series	7.07	0.07067	25.99	0.99	C Vit	Yes	
O	EDS	K series	44.73	0.15053	37.34	0.68	SiO2	Yes	
Na	EDS	K series	5.46	0.02305	5.16	0.20	Albite	Yes	
Si	EDS	K series	18.57	0.14718	15.07	0.29	SiO2	Yes	
Mn	EDS	K series	2.96	0.02957	2.51	0.16	Mn	Yes	
Fe	EDS	K series	16.69	0.16687	13.93	0.36	Fe	Yes	
Total					100.00				

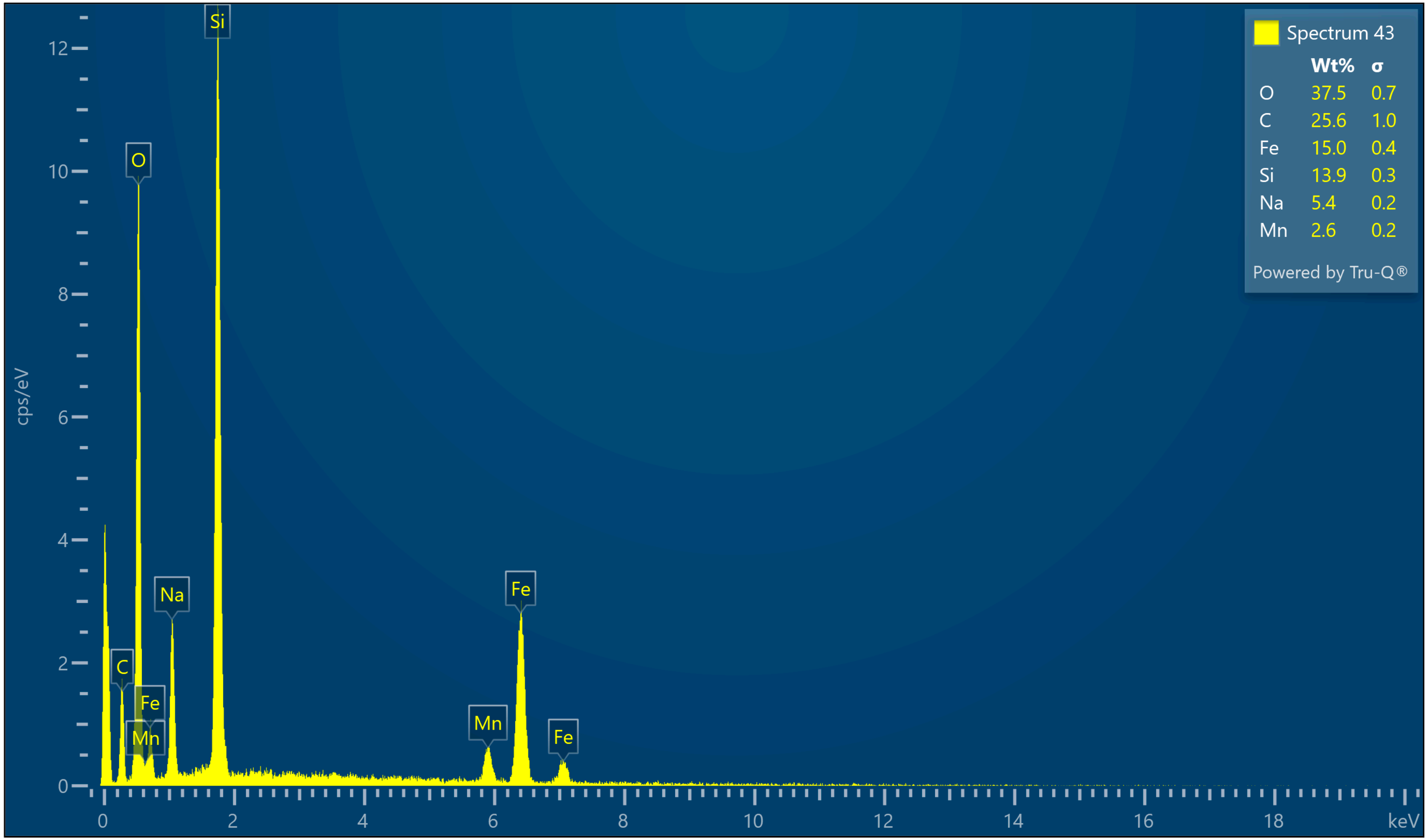
Spectrum 43
Wt% σ
O 37.5 0.7
C 25.6 1.0
Fe 15.0 0.4
Si 13.9 0.3
Na 5.4 0.2
Mn 2.6 0.2
Powered by Tru-Q®
cps/eV
keV
Si
O
Na
C
Fe
Mn
Mn
Fe
Fe
0
2
4
6
8
10
12
14
16
18

Figure S10

Table S10. Spectrum 43									
Element	Signal Type	Line	Apparent Concentration	k Ratio	Wt%	Wt% Sigma	Standard Name	Factory Standard	Standardization Date
C	EDS	K series	7.16	0.07163	25.55	0.99	C Vit	Yes	
O	EDS	K series	46.23	0.15557	37.53	0.68	SiO2	Yes	
Na	EDS	K series	5.69	0.02400	5.45	0.21	Albite	Yes	
Si	EDS	K series	17.05	0.13509	13.92	0.27	SiO2	Yes	
Mn	EDS	K series	3.05	0.03047	2.57	0.16	Mn	Yes	
Fe	EDS	K series	18.07	0.18068	14.98	0.37	Fe	Yes	
Total					100.00				

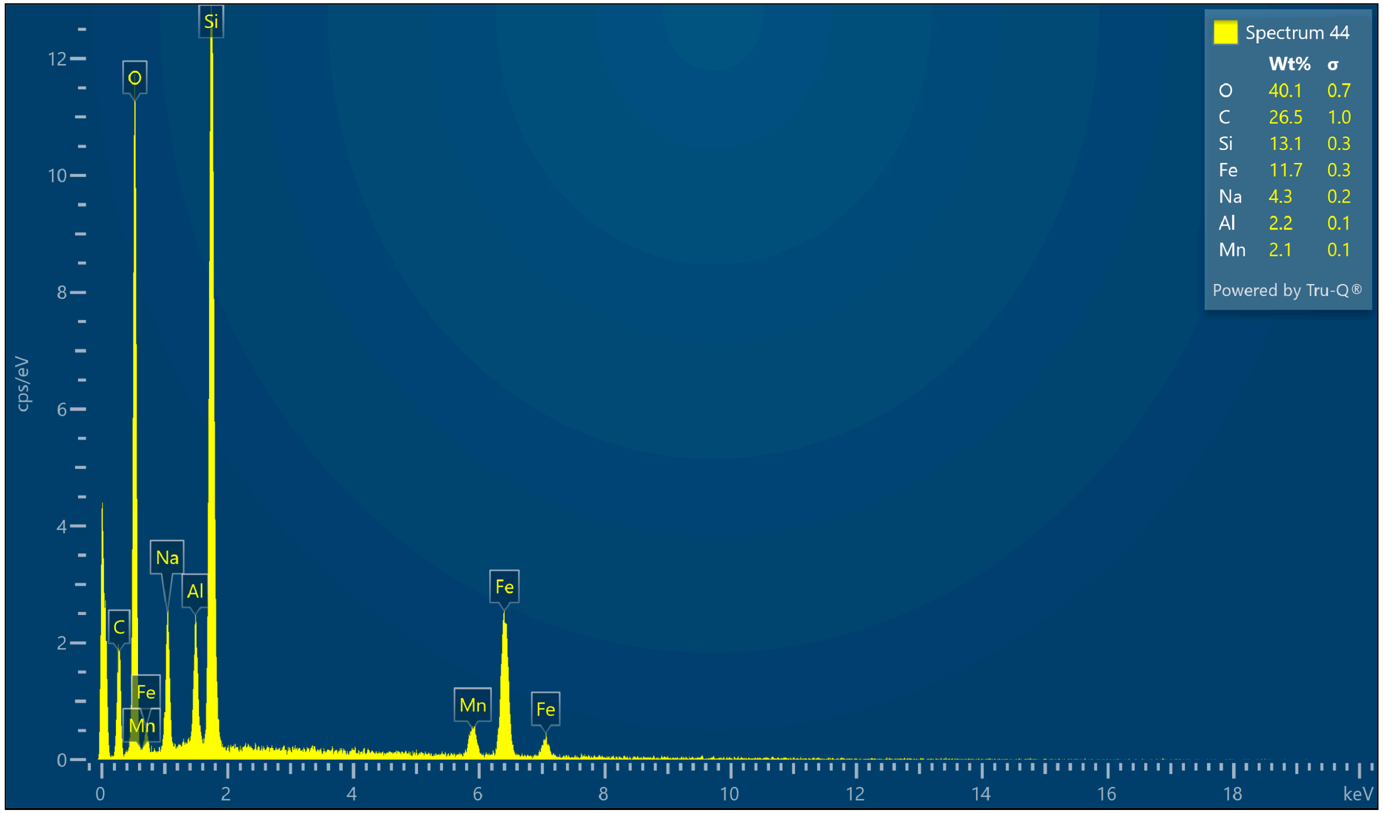
Spectrum 44
Wt% σ
O 40.1 0.7
C 26.5 1.0
Si 13.1 0.3
Fe 11.7 0.3
Na 4.3 0.2
Al 2.2 0.1
Mn 2.1 0.1
Powered by Tru-Q®
cps/eV
keV
Si
O
Na
Al
C
Fe
Mn
Fe
Mn
Fe

Figure S11

Table S11. Spectrum 44									
Element	Signal Type	Line	Apparent Concentration	k Ratio	Wt%	Wt% Sigma	Standard Name	Factory Standard	Standardization Date
C	EDS	K series	8.04	0.08035	26.51	0.97	C Vit	Yes	
O	EDS	K series	51.77	0.17422	40.08	0.68	SiO2	Yes	
Na	EDS	K series	5.02	0.02121	4.32	0.18	Albite	Yes	
Al	EDS	K series	2.63	0.01891	2.19	0.10	Al2O3	Yes	
Si	EDS	K series	17.11	0.13560	13.08	0.26	SiO2	Yes	
Mn	EDS	K series	2.68	0.02683	2.13	0.15	Mn	Yes	
Fe	EDS	K series	14.98	0.14984	11.68	0.31	Fe	Yes	
Total					100.00				

Electron Image 10

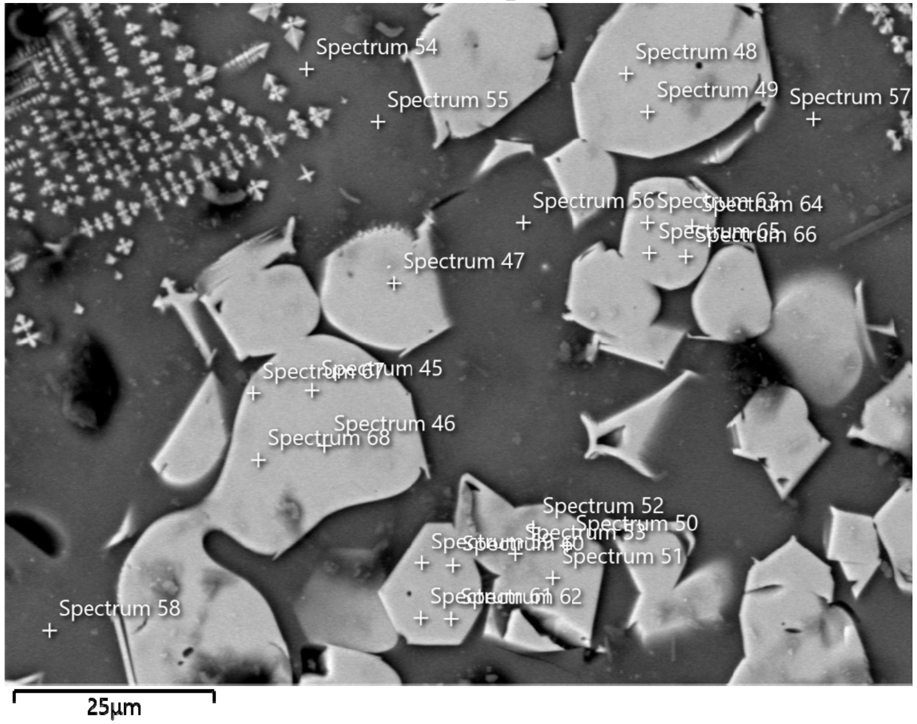

Figure S12

Table S12

Element	45	46	47	48	49	50	51	52	53	54	55	56	57	58
Mn	8,16	7,75	8,05	8,03	8,13	7,53	8,37	8,19	7,72	3,43	3,43	2,94	3,18	3,05
Fe	50,85	51,15	48,53	51,94	51,61	42,54	50,72	48,44	50,07	15,9	15,9	15,12	16,28	15,83
Ratio Fe/Mn	6,2	6,6	6,0	6,5	6,3	5,6	6,1	5,9	6,5	*4,6*	*4,6*	*5,1*	*5,1*	*5,2*
			centre							*Glass*	*Glass*	*Glass*	*Glass*	*Glass*

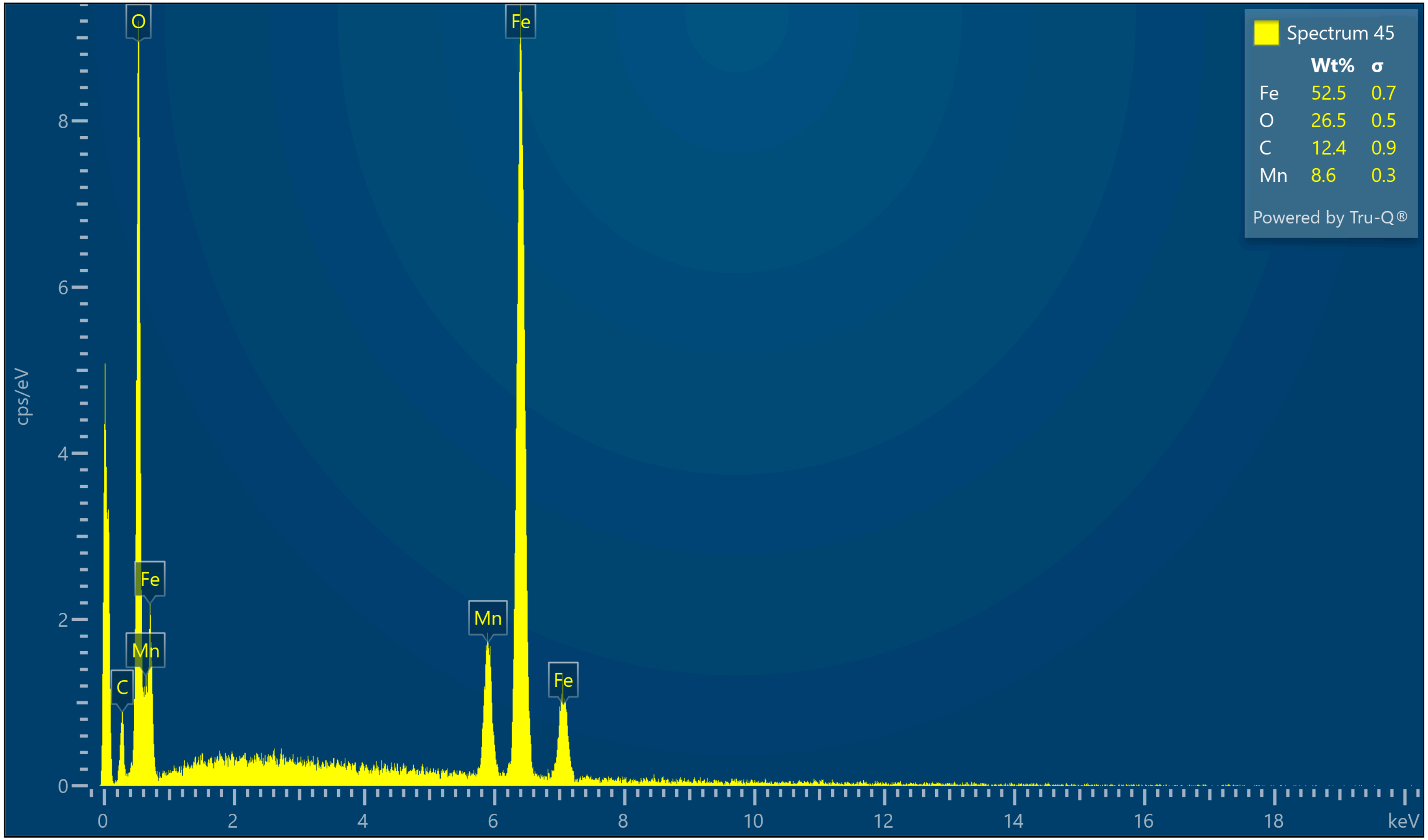
O
Fe
Fe
Mn
C
Mn
Fe
Spectrum 45
Wt% σ
Fe 52.5 0.7
O 26.5 0.5
C 12.4 0.9
Mn 8.6 0.3
Powered by Tru-Q®
cps/eV
0
2
4
6
8
0 2 4 6 8 10 12 14 16 18 keV

Figure S13

Table S13. Spectrum 45

Element	Signal Type	Line	Apparent Concentration	k Ratio	Wt%	Wt% Sigma	Standard Name	Factory Standard	Standardization Date
C	EDS	K series	3.22	0.03222	12.38	0.89	C Vit	Yes	
O	EDS	K series	37.49	0.12615	26.54	0.54	SiO2	Yes	
Mn	EDS	K series	8.16	0.08158	8.58	0.31	Mn	Yes	
Fe	EDS	K series	50.85	0.50851	52.50	0.71	Fe	Yes	
Total					100.00				

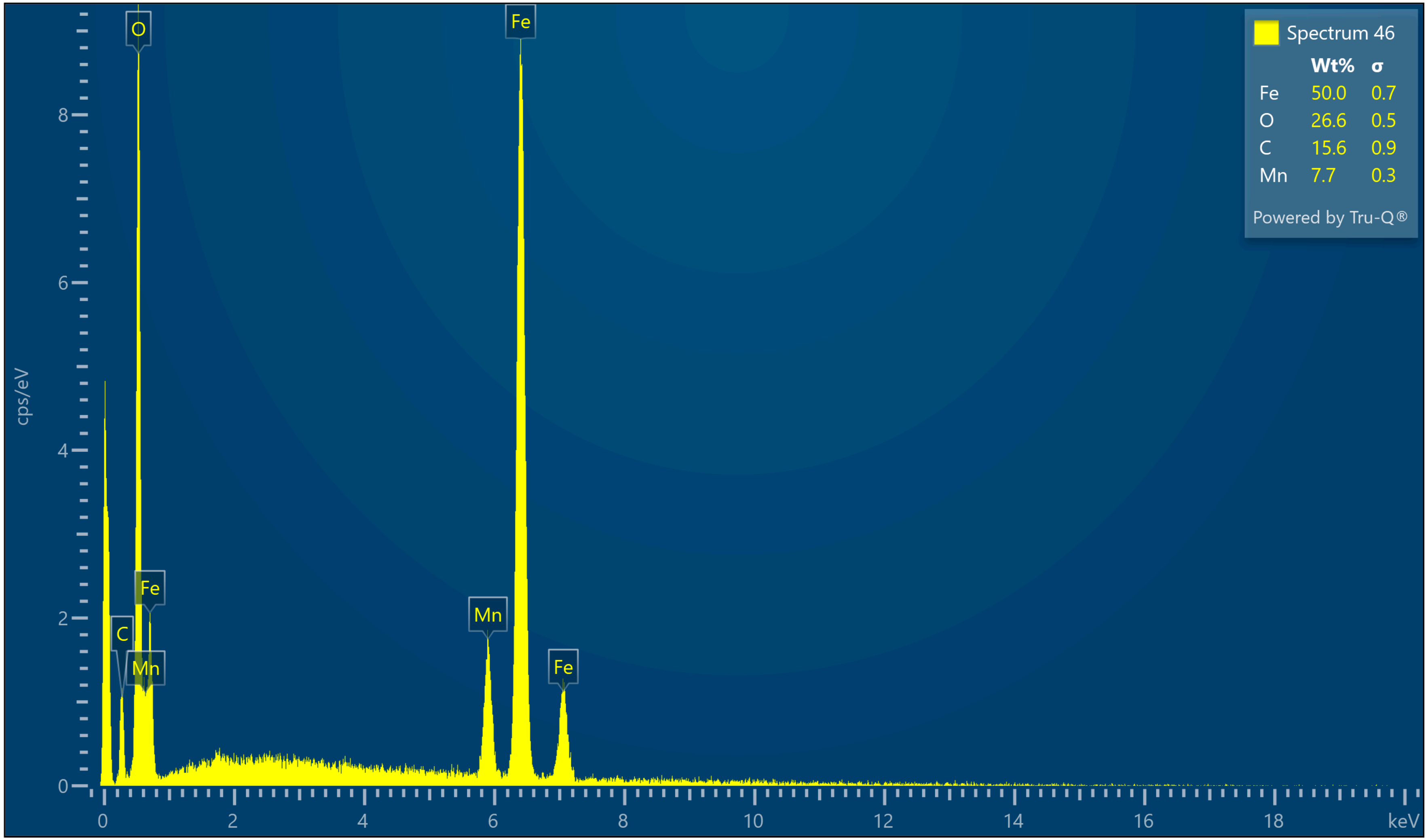

Figure S14

OXFORD INSTRUMENTS

Table S14. Spectrum 46

Element	Signal Type	Line	Apparent Concentration	k Ratio	Wt%	Wt% Sigma	Standard Name	Factory Standard	Standardization Date
C	EDS	K series	4.48	0.04478	15.63	0.88	C Vit	Yes	
O	EDS	K series	37.22	0.12526	26.61	0.54	SiO2	Yes	
Mn	EDS	K series	7.75	0.07754	7.73	0.28	Mn	Yes	
Fe	EDS	K series	51.15	0.51150	50.03	0.69	Fe	Yes	
Total					100.00				

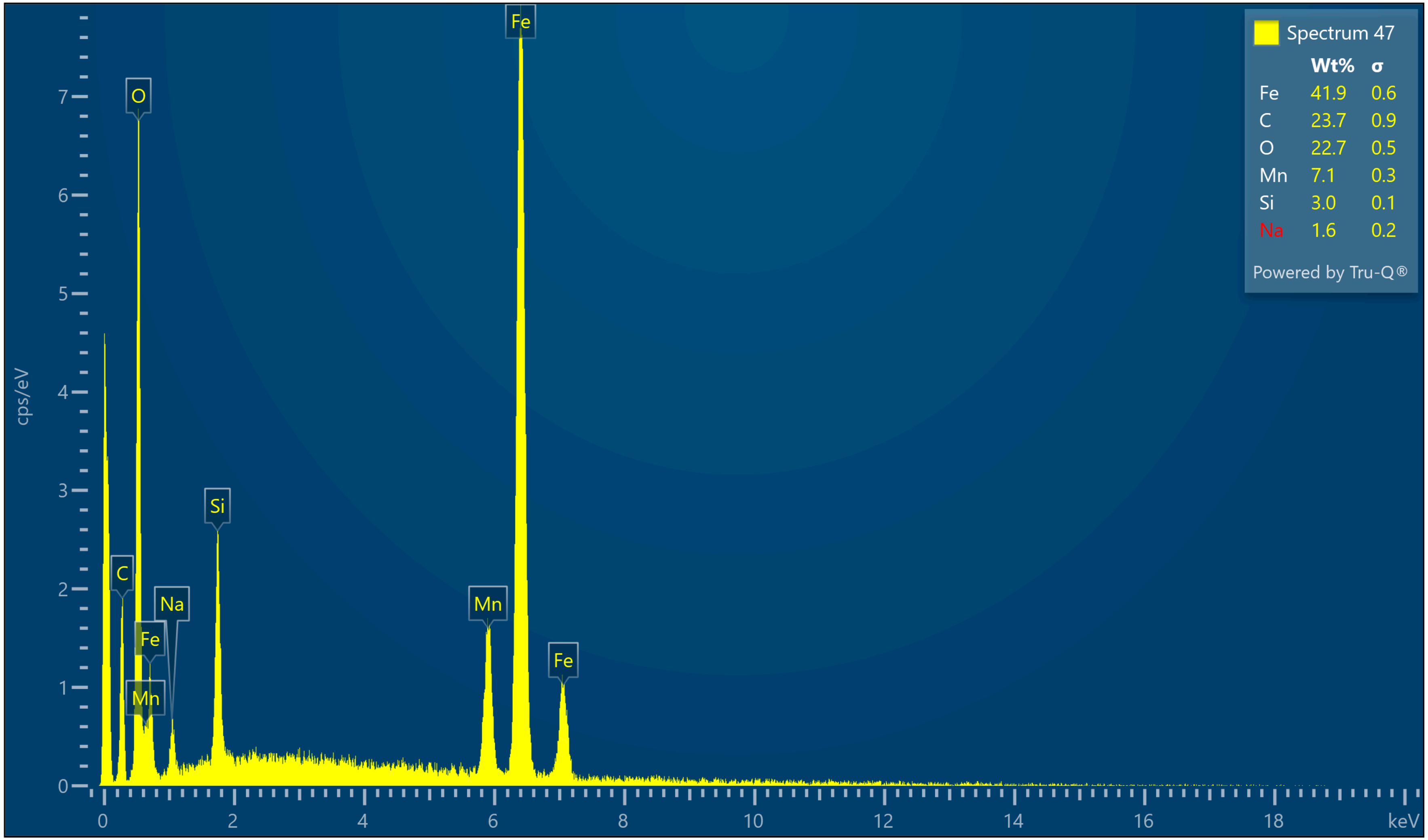
Spectrum 47
Wt% σ
Fe 41.9 0.6
C 23.7 0.9
O 22.7 0.5
Mn 7.1 0.3
Si 3.0 0.1
Na 1.6 0.2
Powered by Tru-Q®
cps/eV
keV
Fe
O
Si
C
Na
Fe
Mn
Mn
Fe
0 1 2 3 4 5 6 7
0 2 4 6 8 10 12 14 16 18

Figure S15

Table S15. Spectrum 47

Element	Signal Type	Line	Apparent Concentration	k Ratio	Wt%	Wt% Sigma	Standard Name	Factory Standard	Standardization Date
C	EDS	K series	7.57	0.07566	23.69	0.88	C Vit	Yes	
O	EDS	K series	29.41	0.09897	22.72	0.51	SiO2	Yes	
Na	EDS	K series	1.05	0.00443	1.56	0.17	Albite	Yes	
Si	EDS	K series	2.95	0.02336	3.01	0.13	SiO2	Yes	
Mn	EDS	K series	8.05	0.08046	7.08	0.26	Mn	Yes	
Fe	EDS	K series	48.53	0.48534	41.95	0.63	Fe	Yes	
Total					100.00				

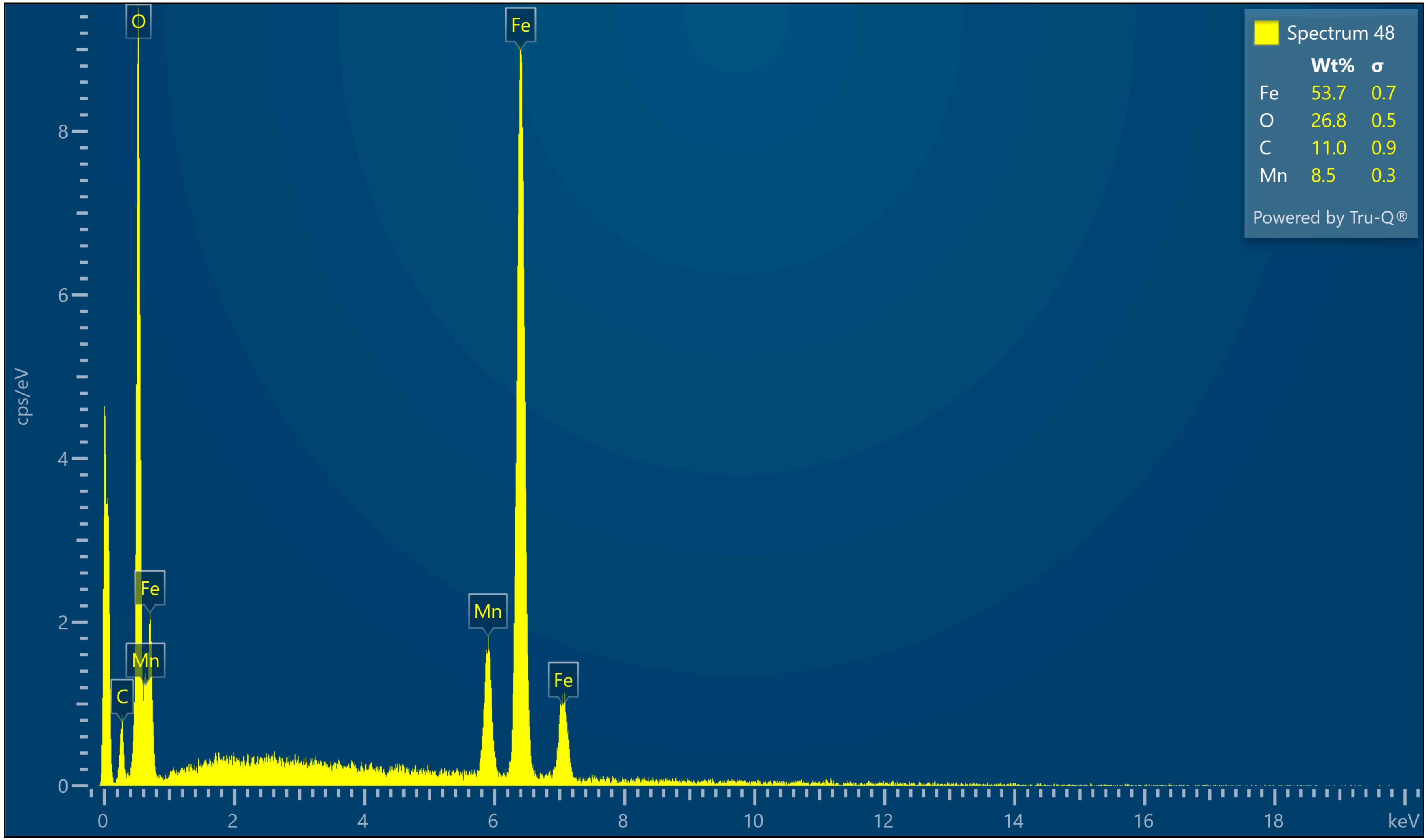

Figure S16

OXFORD INSTRUMENTS

Table S16. Spectrum 48									
Element	Signal Type	Line	Apparent Concentration	k Ratio	Wt%	Wt% Sigma	Standard Name	Factory Standard	Standardization Date
C	EDS	K series	2.82	0.02819	11.01	0.89	C Vit	Yes	
O	EDS	K series	38.87	0.13080	26.79	0.54	SiO2	Yes	
Mn	EDS	K series	8.03	0.08031	8.47	0.30	Mn	Yes	
Fe	EDS	K series	51.94	0.51943	53.73	0.71	Fe	Yes	
Total					100.00				

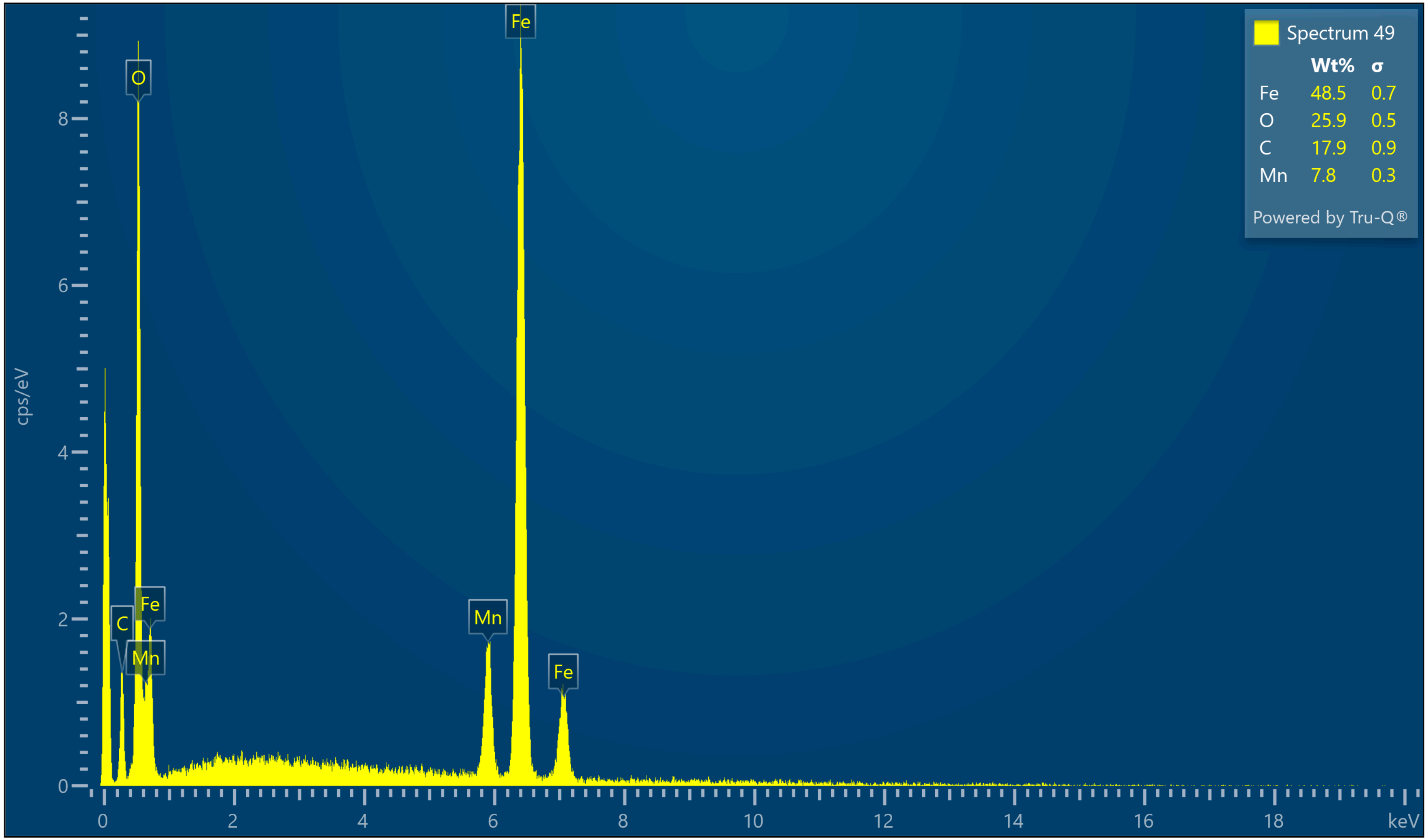

Spectrum 49
Wt% σ
Fe 48.5 0.7
O 25.9 0.5
C 17.9 0.9
Mn 7.8 0.3
Powered by Tru-Q®
cps/eV
keV
Fe
O
C
Mn

Figure S17

Table S17. Spectrum 49

Element	Signal Type	Line	Apparent Concentration	k Ratio	Wt%	Wt% Sigma	Standard Name	Factory Standard	Standardization Date
C	EDS	K series	5.47	0.05474	17.91	0.86	C Vit	Yes	
O	EDS	K series	35.97	0.12105	25.87	0.53	SiO2	Yes	
Mn	EDS	K series	8.13	0.08128	7.77	0.28	Mn	Yes	
Fe	EDS	K series	51.61	0.51609	48.45	0.67	Fe	Yes	
Total					100.00				

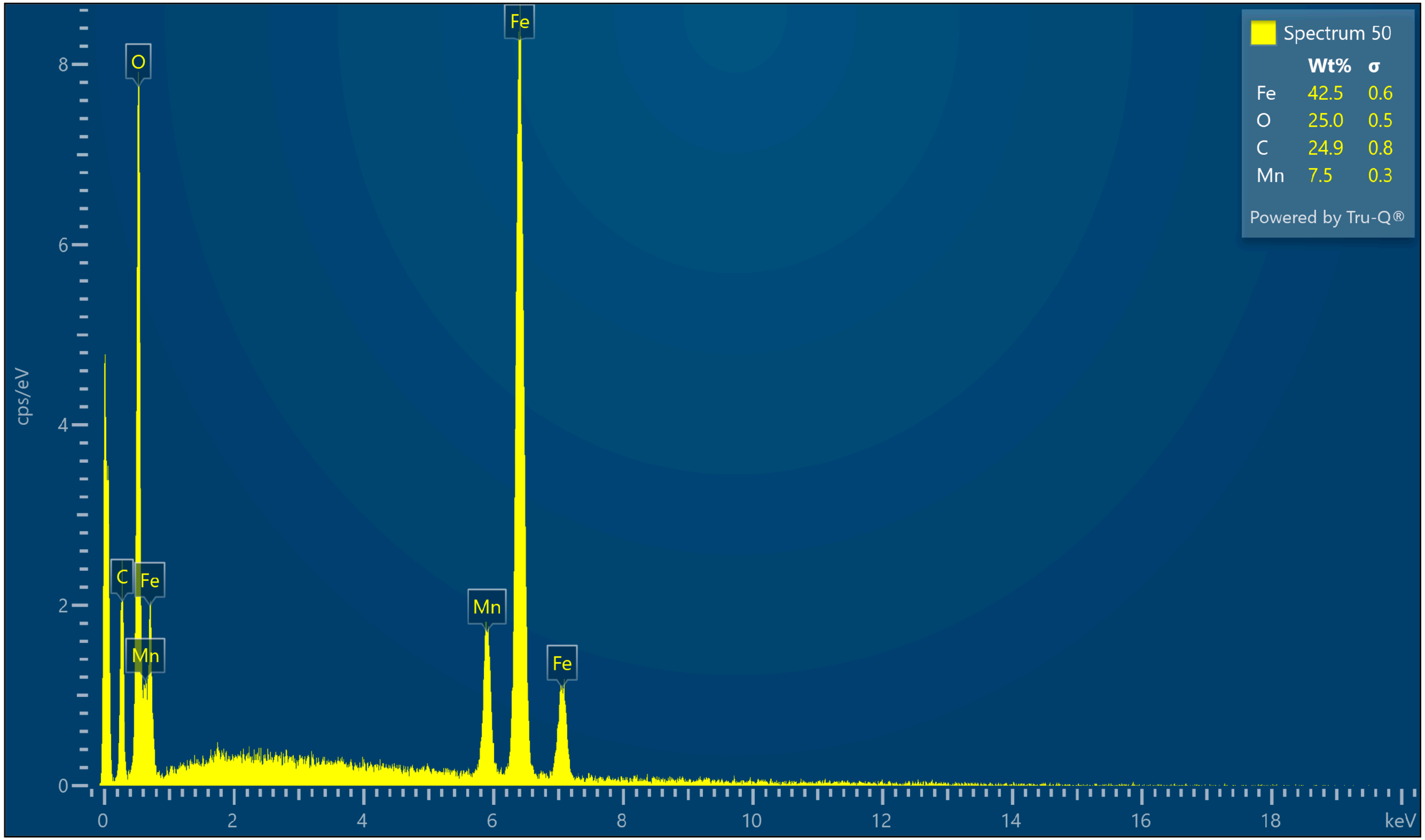
Spectrum 50
Wt% σ
Fe 42.5 0.6
O 25.0 0.5
C 24.9 0.8
Mn 7.5 0.3
Powered by Tru-Q®
cps/eV
keV
O
Fe
C
Fe
Mn
Mn
Fe
0
2
4
6
8
0
2
4
6
8
10
12
14
16
18

Figure S18

Table S18. Spectrum 50									
Element	Signal Type	Line	Apparent Concentration	k Ratio	Wt%	Wt% Sigma	Standard Name	Factory Standard	Standardization Date
C	EDS	K series	9.14	0.09138	24.92	0.84	C Vit	Yes	
O	EDS	K series	33.41	0.11244	25.02	0.54	SiO2	Yes	
Mn	EDS	K series	8.63	0.08630	7.53	0.26	Mn	Yes	
Fe	EDS	K series	49.65	0.49649	42.54	0.62	Fe	Yes	
Total					100.00				

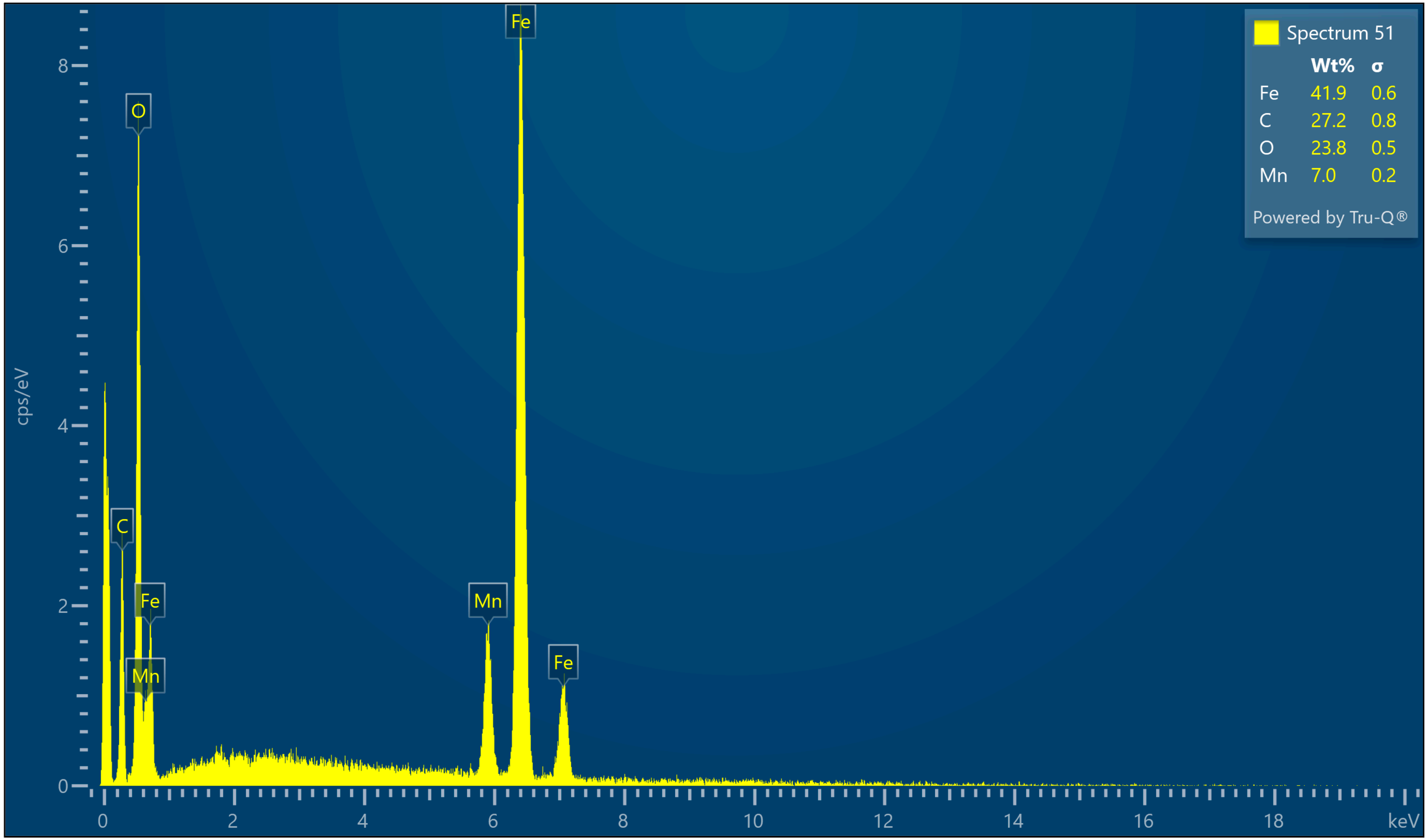
Spectrum 51
Wt% σ
Fe 41.9 0.6
C 27.2 0.8
O 23.8 0.5
Mn 7.0 0.2
Powered by Tru-Q®
cps/eV
keV
Fe
O
C
Fe
Mn
Mn
Fe
0 2 4 6 8
0 2 4 6 8 10 12 14 16 18

Figure S19

Table S19. Spectrum 51

Element	Signal Type	Line	Apparent Concentration	k Ratio	Wt%	Wt% Sigma	Standard Name	Factory Standard	Standardization Date
C	EDS	K series	10.55	0.10554	27.20	0.81	C Vit	Yes	
O	EDS	K series	31.47	0.10591	23.82	0.52	SiO2	Yes	
Mn	EDS	K series	8.37	0.08368	7.04	0.25	Mn	Yes	
Fe	EDS	K series	50.72	0.50718	41.94	0.61	Fe	Yes	
Total					100.00				

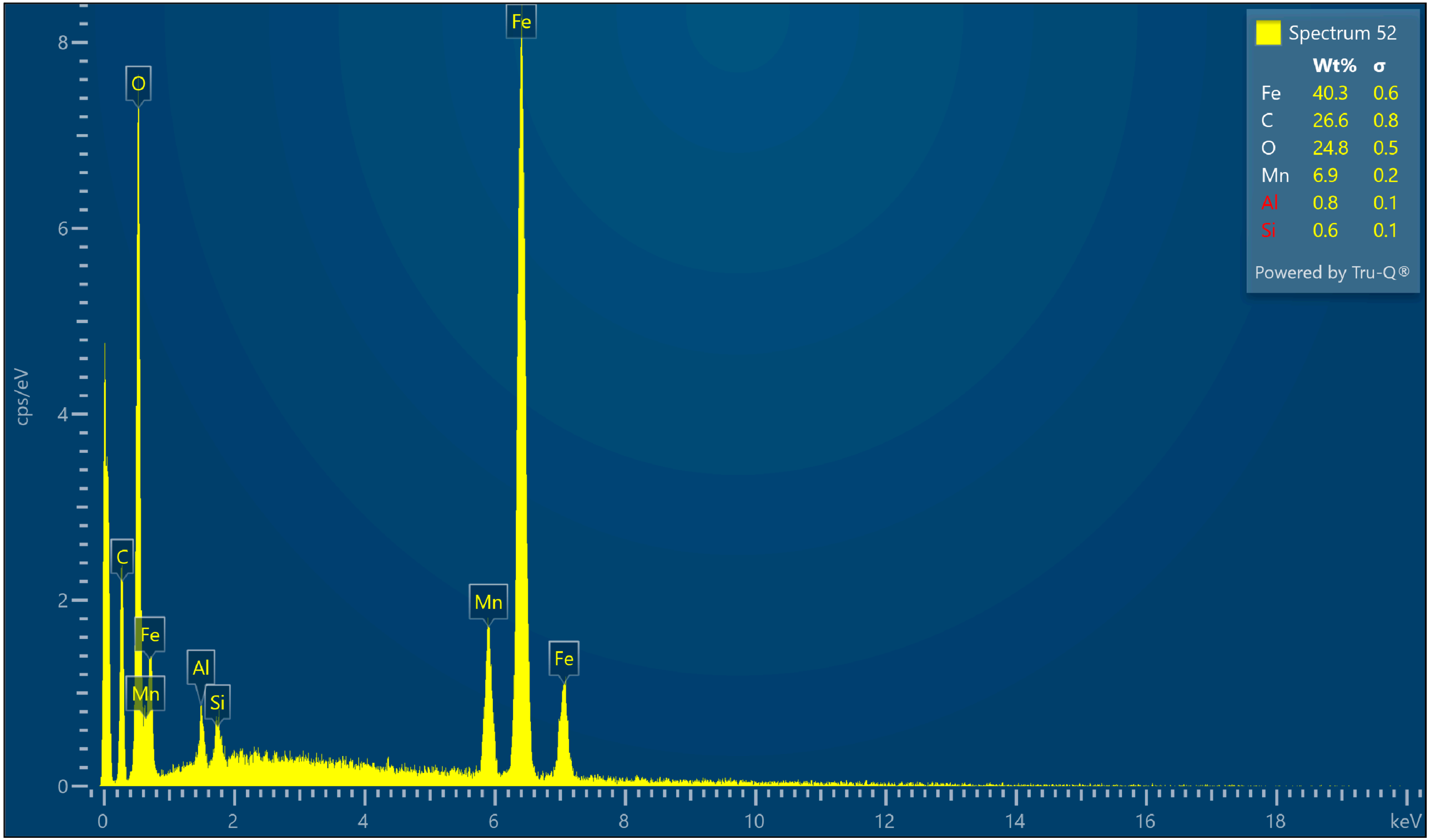
Spectrum 52
Wt% σ
Fe 40.3 0.6
C 26.6 0.8
O 24.8 0.5
Mn 6.9 0.2
Al 0.8 0.1
Si 0.6 0.1
Powered by Tru-Q®
cps/eV
keV
Fe
O
C
Fe
Mn
Al
Si
Mn
Fe

Figure S20

Table S20. Spectrum 52

Element	Signal Type	Line	Apparent Concentration	k Ratio	Wt%	Wt% Sigma	Standard Name	Factory Standard	Standardization Date
C	EDS	K series	9.91	0.09912	26.57	0.84	C Vit	Yes	
O	EDS	K series	32.67	0.10994	24.79	0.53	SiO2	Yes	
Al	EDS	K series	0.72	0.00514	0.82	0.09	Al2O3	Yes	
Si	EDS	K series	0.59	0.00470	0.58	0.08	SiO2	Yes	
Mn	EDS	K series	8.19	0.08185	6.93	0.25	Mn	Yes	
Fe	EDS	K series	48.44	0.48439	40.31	0.61	Fe	Yes	
Total					100.00				

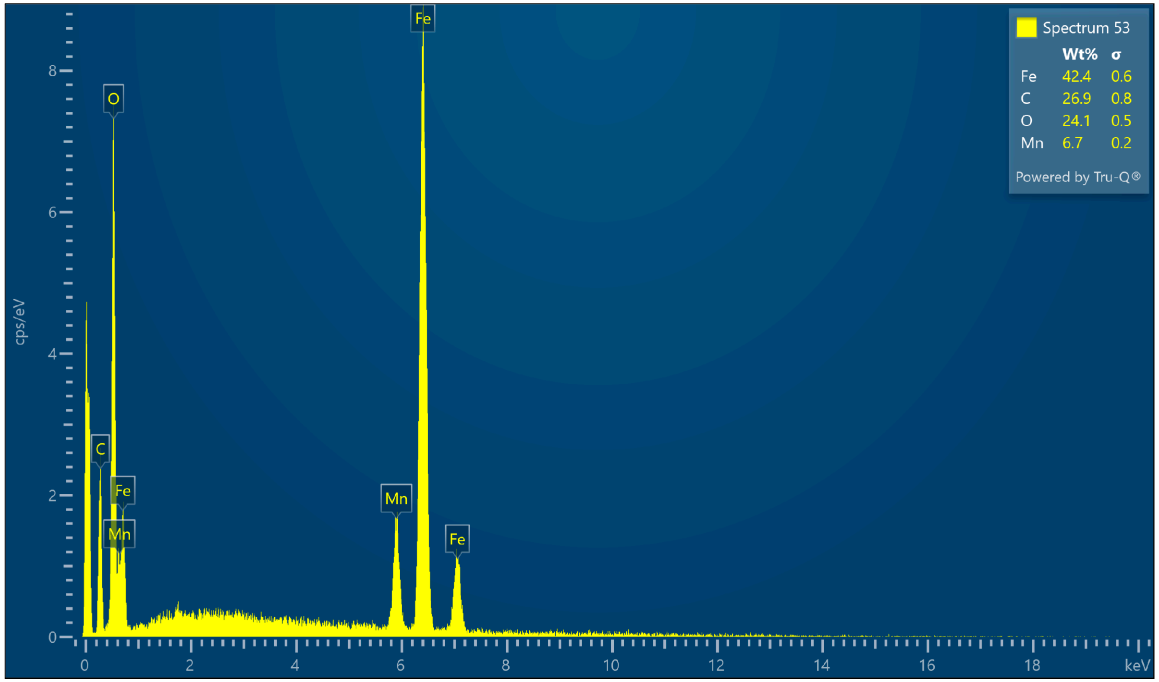

Figure S21

Table S21. Spectrum 53

Element	Signal Type	Line	Apparent Concentration	k Ratio	Wt%	Wt% Sigma	Standard Name	Factory Standard	Standardization Date
C	EDS	K series	10.14	0.10140	26.86	0.83	C Vit	Yes	
O	EDS	K series	31.23	0.10509	24.06	0.53	SiO2	Yes	
Mn	EDS	K series	7.72	0.07715	6.65	0.25	Mn	Yes	
Fe	EDS	K series	50.07	0.50073	42.43	0.62	Fe	Yes	

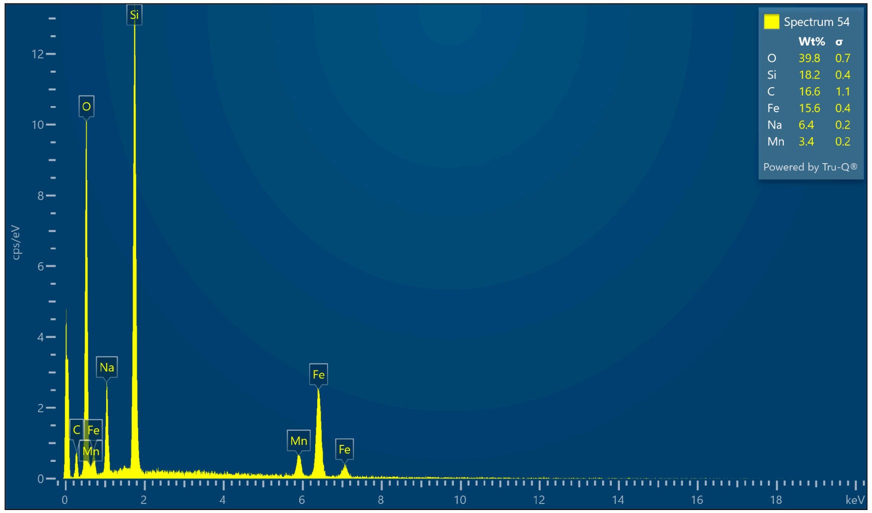

Figure S22

**Table S22. Spectrum 54**									
Element	Signal Type	Line	Apparent Concentration	k Ratio	Wt%	Wt% Sigma	Standard Name	Factory Standard	Standardization Date
C	EDS	K series	3.28	0.03280	16.56	1.09	C Vit	Yes	
O	EDS	K series	47.05	0.15833	39.79	0.71	SiO2	Yes	
Na	EDS	K series	5.51	0.02324	6.38	0.24	Albite	Yes	
Si	EDS	K series	18.47	0.14639	18.22	0.35	SiO2	Yes	
Mn	EDS	K series	3.43	0.03426	3.43	0.20	Mn	Yes	
Fe	EDS	K series	15.90	0.15895	15.62	0.40	Fe	Yes	
Total					100.00				

OXFORD INSTRUMENTS

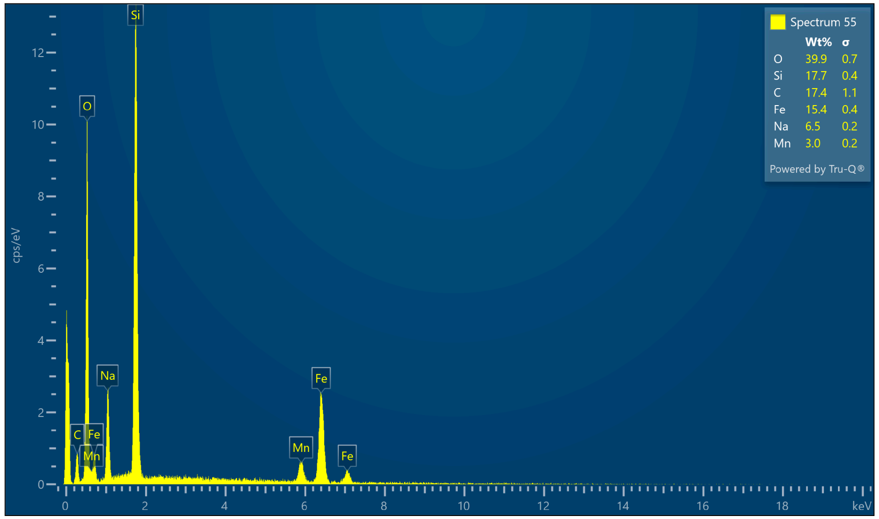

Figure S23

Table S23. Spectrum 55									
Element	Signal Type	Line	Apparent Concentration	k Ratio	Wt%	Wt% Sigma	Standard Name	Factory Standard	Standardization Date
C	EDS	K series	3.56	0.03557	17.41	1.14	C Vit	Yes	
O	EDS	K series	47.40	0.15949	39.94	0.73	SiO2	Yes	
Na	EDS	K series	5.72	0.02414	6.49	0.24	Albite	Yes	
Si	EDS	K series	18.29	0.14492	17.74	0.35	SiO2	Yes	
Mn	EDS	K series	3.05	0.03054	3.01	0.20	Mn	Yes	
Fe	EDS	K series	15.91	0.15906	15.41	0.40	Fe	Yes	
Total					100.00				

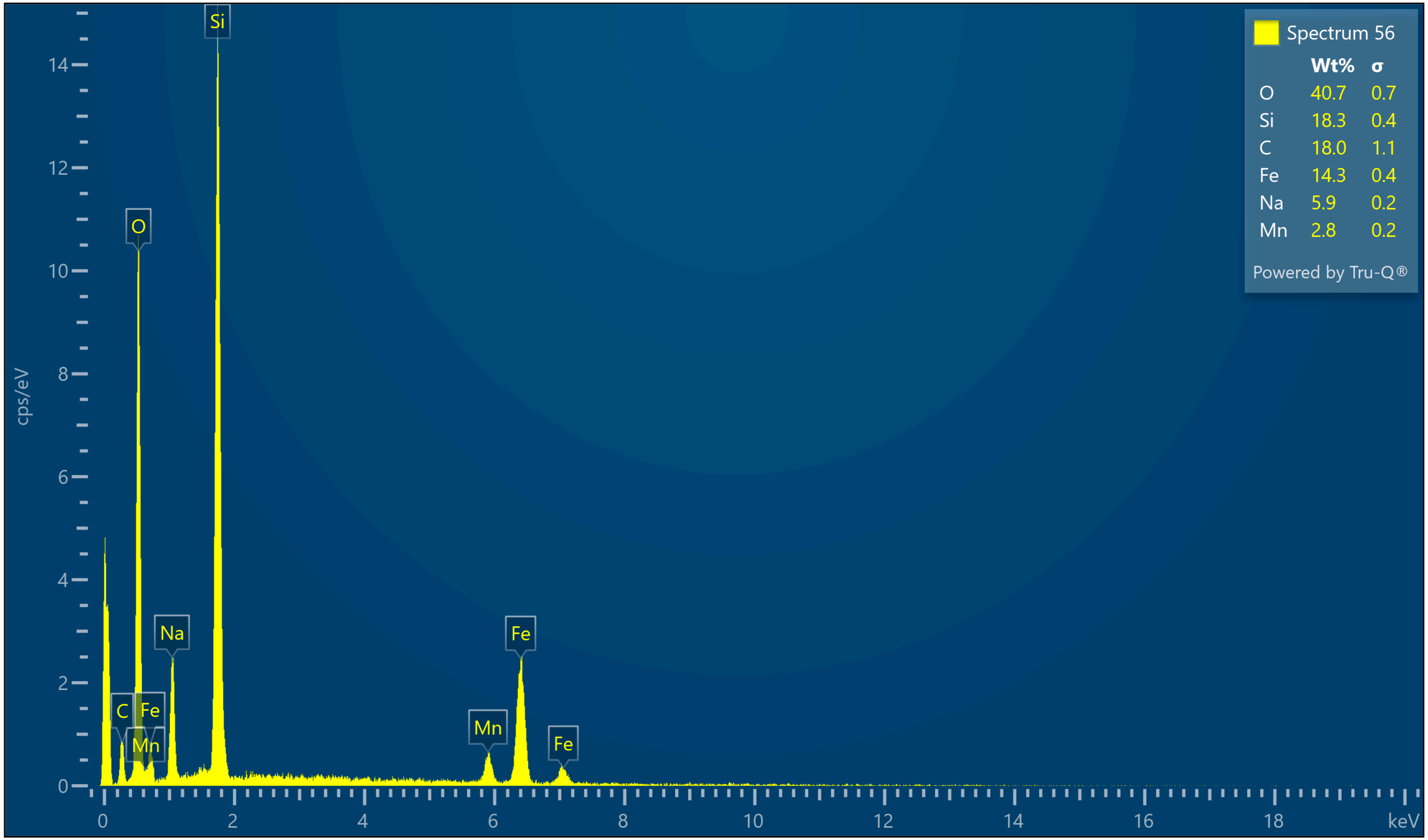
Spectrum 56
Wt% σ
O 40.7 0.7
Si 18.3 0.4
C 18.0 1.1
Fe 14.3 0.4
Na 5.9 0.2
Mn 2.8 0.2
Powered by Tru-Q®
cps/eV
keV
Si
O
Na
C
Fe
Mn
Mn
Fe
Fe

Figure S24

Table S24. Spectrum 56

Element	Signal Type	Line	Apparent Concentration	k Ratio	Wt%	Wt% Sigma	Standard Name	Factory Standard	Standardization Date
C	EDS	K series	3.77	0.03770	18.01	1.11	C Vit	Yes	
O	EDS	K series	48.70	0.16388	40.67	0.73	SiO2	Yes	
Na	EDS	K series	5.40	0.02279	5.88	0.23	Albite	Yes	
Si	EDS	K series	19.61	0.15540	18.31	0.35	SiO2	Yes	
Mn	EDS	K series	2.94	0.02939	2.83	0.18	Mn	Yes	
Fe	EDS	K series	15.12	0.15116	14.30	0.38	Fe	Yes	
Total					100.00				

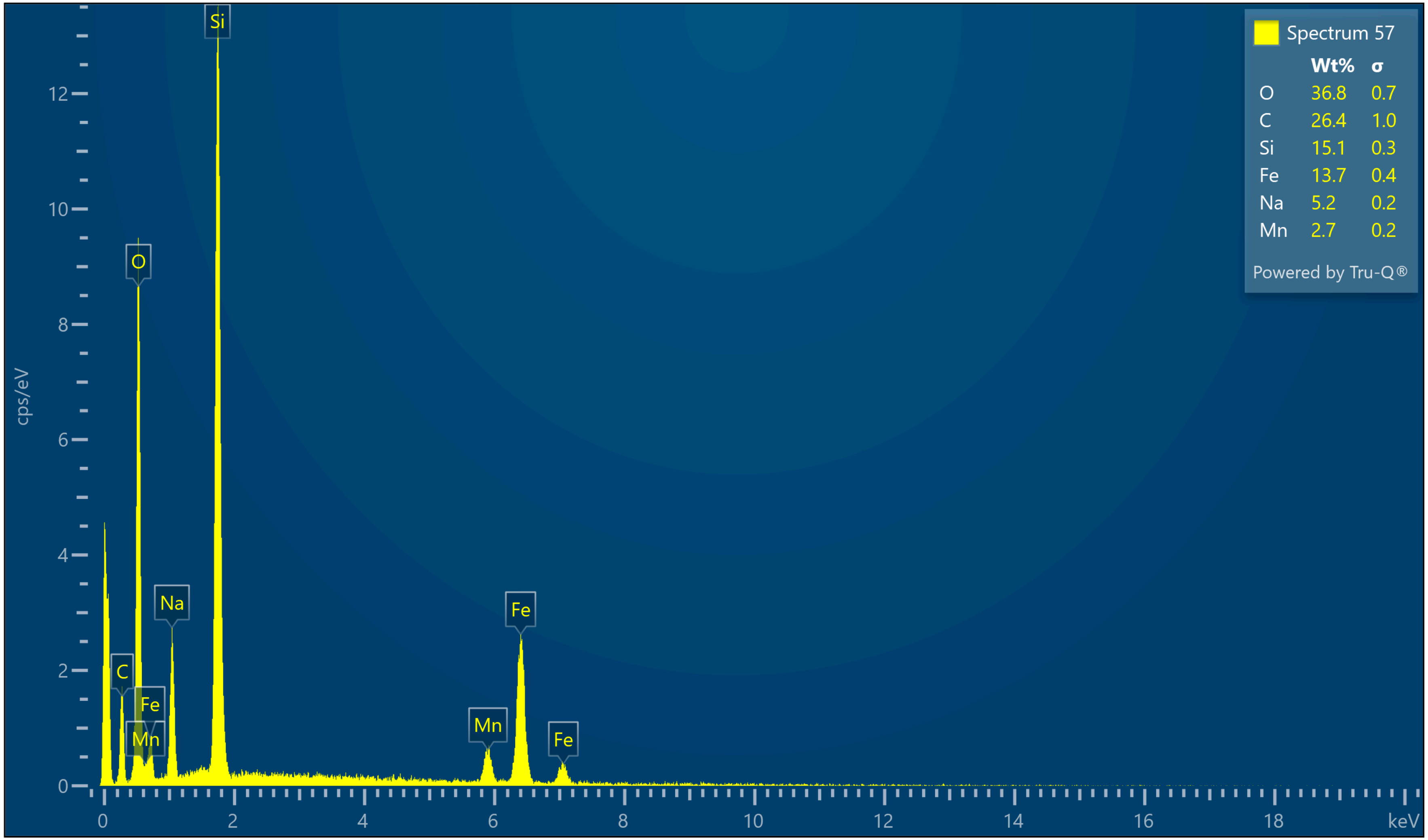
Spectrum 57
Wt% σ
O 36.8 0.7
C 26.4 1.0
Si 15.1 0.3
Fe 13.7 0.4
Na 5.2 0.2
Mn 2.7 0.2
Powered by Tru-Q®
cps/eV
keV
Si
O
Na
C
Fe
Mn
Fe
Mn
Fe
0
2
4
6
8
10
12
14
16
18

Figure S25

Table S25. Spectrum 57

Element	Signal Type	Line	Apparent Concentration	k Ratio	Wt%	Wt% Sigma	Standard Name	Factory Standard	Standardization Date
C	EDS	K series	7.12	0.07125	26.44	1.01	C Vit	Yes	
O	EDS	K series	43.22	0.14543	36.77	0.68	SiO2	Yes	
Na	EDS	K series	5.51	0.02327	5.23	0.20	Albite	Yes	
Si	EDS	K series	18.52	0.14676	15.14	0.30	SiO2	Yes	
Mn	EDS	K series	3.18	0.03183	2.73	0.17	Mn	Yes	
Fe	EDS	K series	16.28	0.16277	13.70	0.35	Fe	Yes	
Total					100.00				

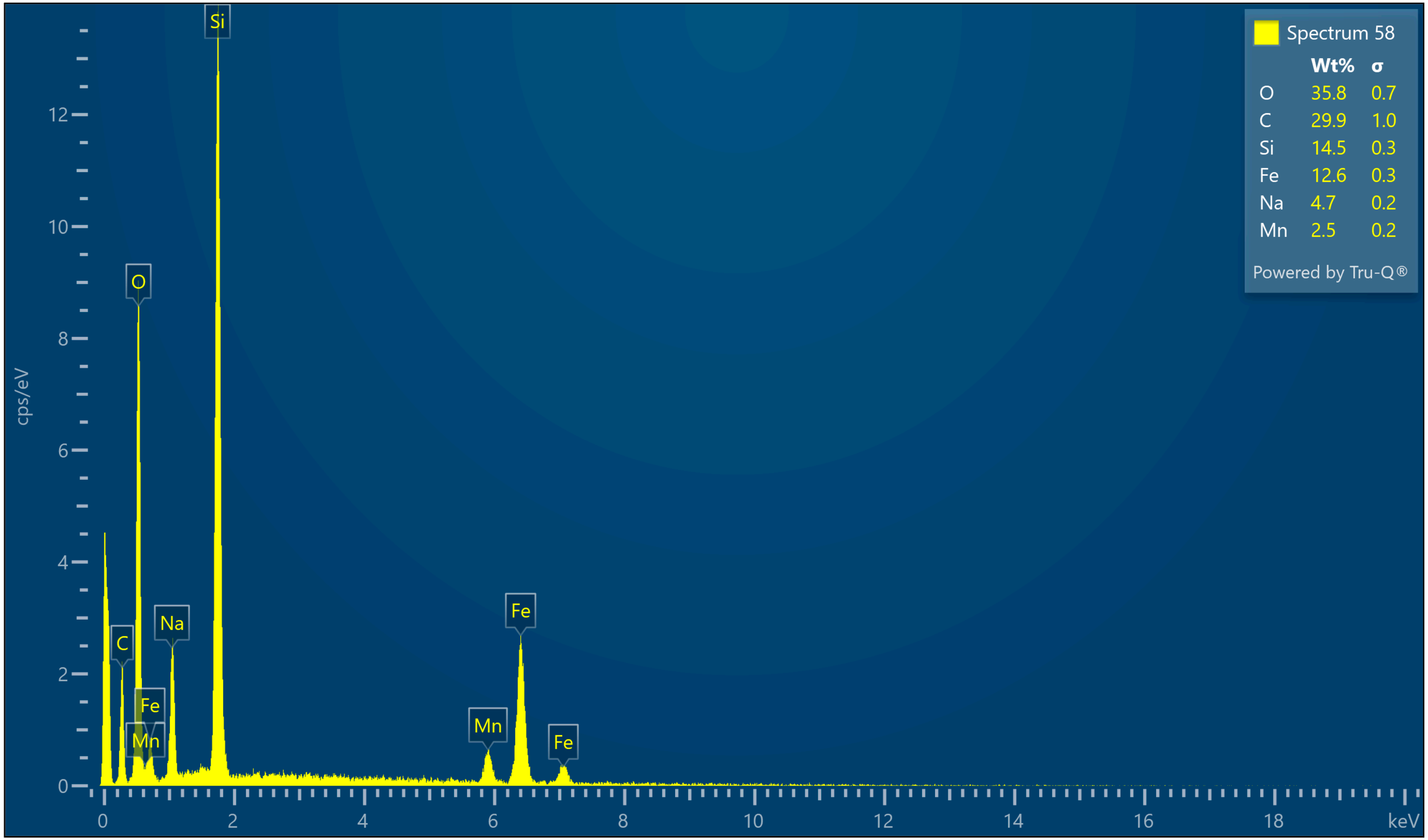

Figure S26

OXFORD INSTRUMENTS

Table S26. Spectrum 58

Element	Signal Type	Line	Apparent Concentration	k Ratio	Wt%	Wt% Sigma	Standard Name	Factory Standard	Standardization Date
C	EDS	K series	8.94	0.08938	29.87	0.95	C Vit	Yes	
O	EDS	K series	42.06	0.14154	35.81	0.67	SiO2	Yes	
Na	EDS	K series	5.41	0.02284	4.74	0.18	Albite	Yes	
Si	EDS	K series	19.09	0.15124	14.51	0.28	SiO2	Yes	
Mn	EDS	K series	3.05	0.03053	2.47	0.16	Mn	Yes	
Fe	EDS	K series	15.83	0.15835	12.60	0.33	Fe	Yes	
Total					100.00				

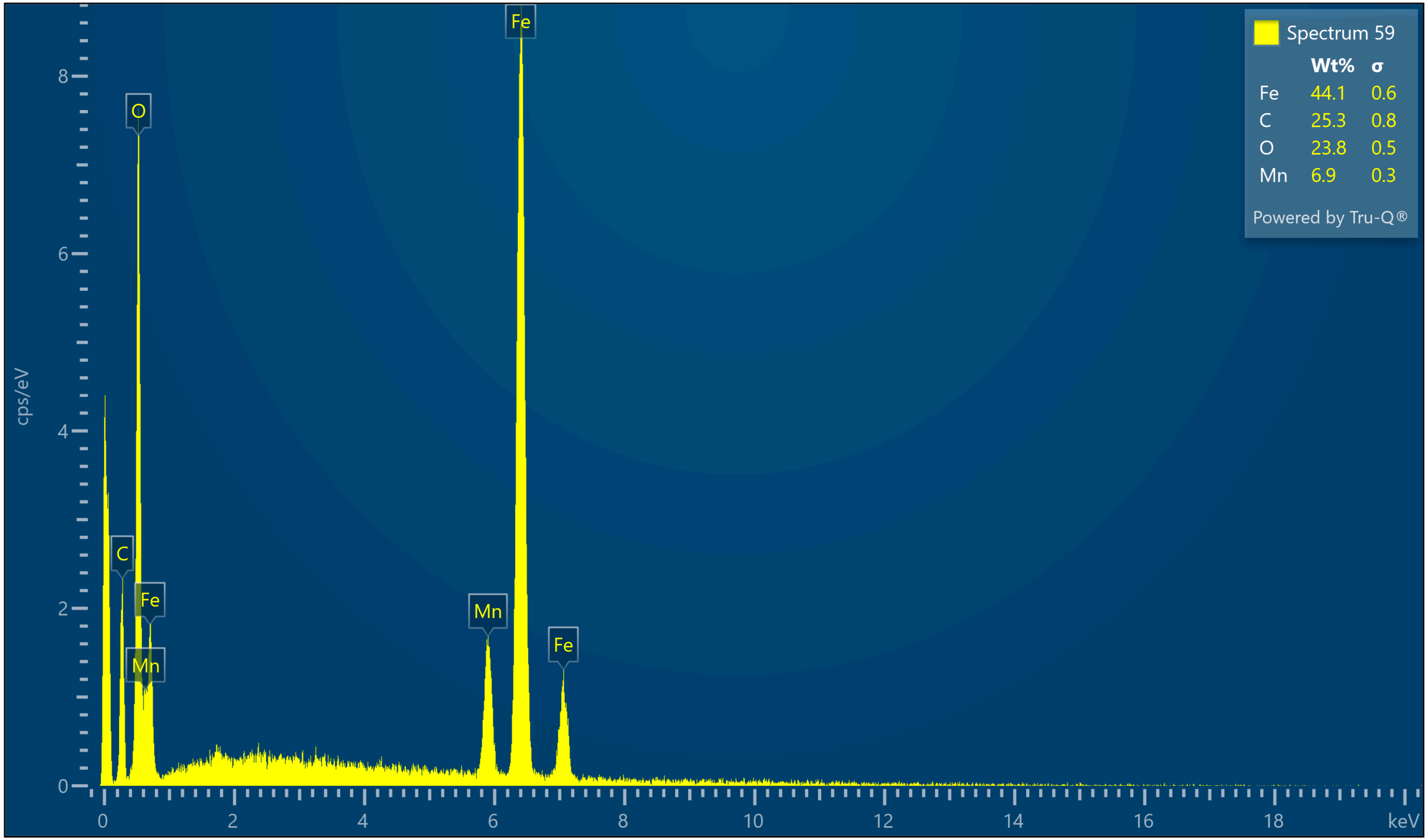
Spectrum 59
Wt% σ
Fe 44.1 0.6
C 25.3 0.8
O 23.8 0.5
Mn 6.9 0.3
Powered by Tru-Q®
cps/eV
keV
Fe
O
C
Fe
Mn
Mn
Fe

Figure S27

Table S27. Spectrum 59									
Element	Signal Type	Line	Apparent Concentration	k Ratio	Wt%	Wt% Sigma	Standard Name	Factory Standard	Standardization Date
C	EDS	K series	9.21	0.09213	25.26	0.84	C Vit	Yes	
O	EDS	K series	31.44	0.10581	23.79	0.53	SiO2	Yes	
Mn	EDS	K series	7.88	0.07883	6.86	0.25	Mn	Yes	
Fe	EDS	K series	51.60	0.51596	44.09	0.64	Fe	Yes	
Total					100.00				

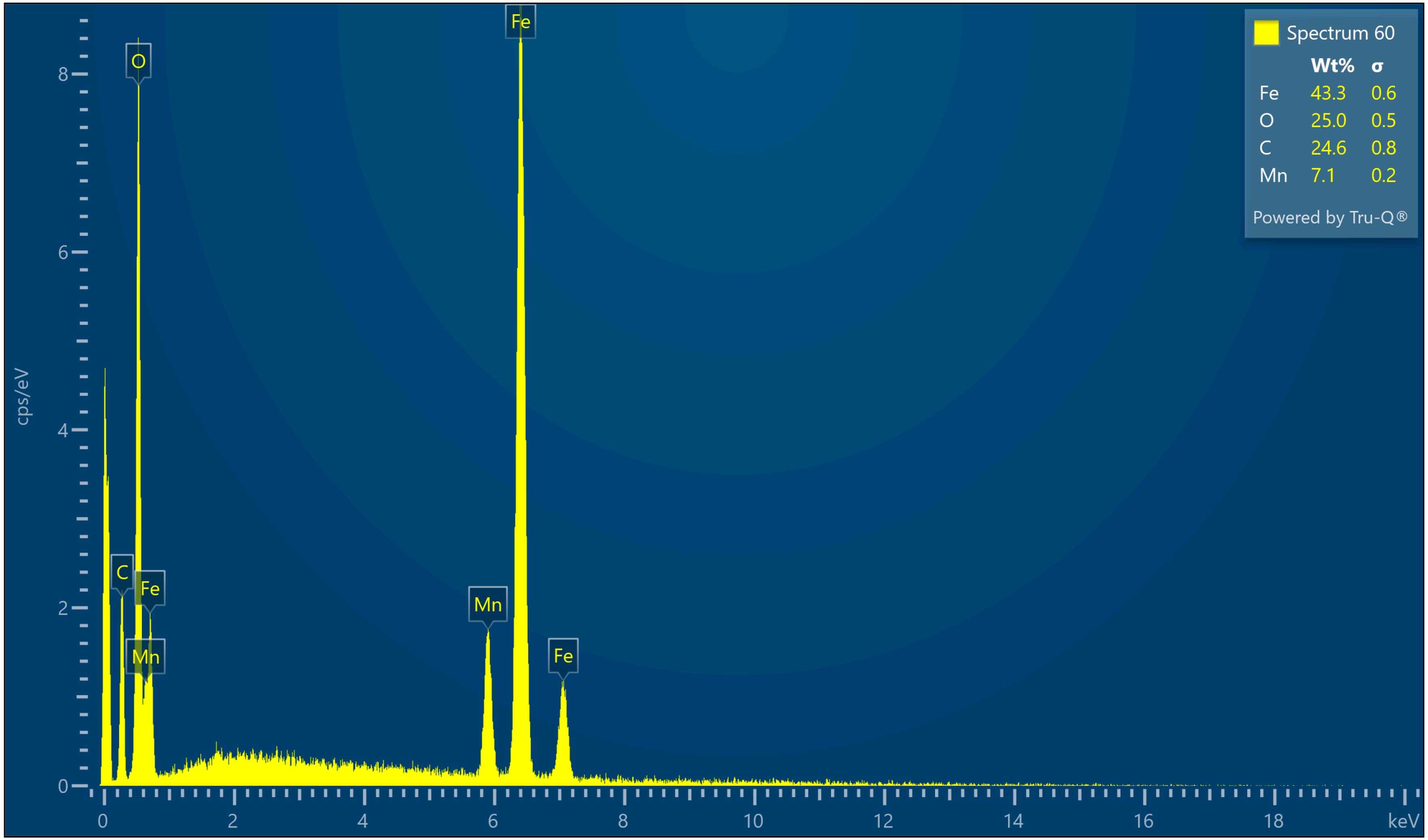

Figure S28

OXFORD INSTRUMENTS

Table S28. Spectrum 60									
Element	Signal Type	Line	Apparent Concentration	k Ratio	Wt%	Wt% Sigma	Standard Name	Factory Standard	Standardization Date
C	EDS	K series	9.05	0.09046	24.57	0.82	C Vit	Yes	
O	EDS	K series	33.95	0.11423	25.03	0.53	SiO2	Yes	
Mn	EDS	K series	8.21	0.08209	7.09	0.25	Mn	Yes	
Fe	EDS	K series	51.05	0.51054	43.31	0.62	Fe	Yes	
Total					100.00				

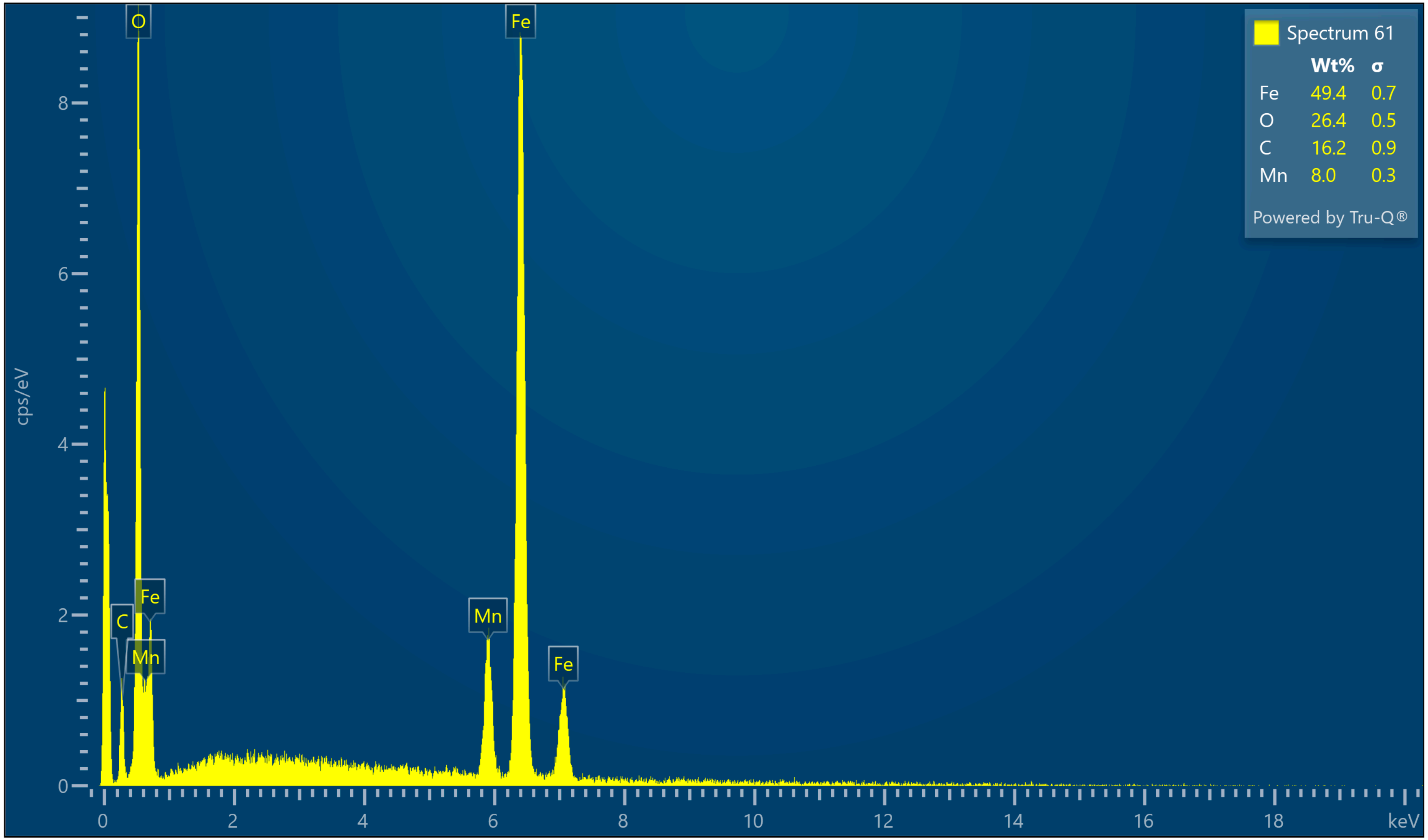

Figure S29

OXFORD INSTRUMENTS

Table S29. Spectrum 61

Element	Signal Type	Line	Apparent Concentration	k Ratio	Wt%	Wt% Sigma	Standard Name	Factory Standard	Standardization Date
C	EDS	K series	4.71	0.04714	16.19	0.86	C Vit	Yes	
O	EDS	K series	36.94	0.12431	26.43	0.54	SiO2	Yes	
Mn	EDS	K series	8.13	0.08133	8.02	0.28	Mn	Yes	
Fe	EDS	K series	51.00	0.50997	49.36	0.67	Fe	Yes	
Total					100.00				

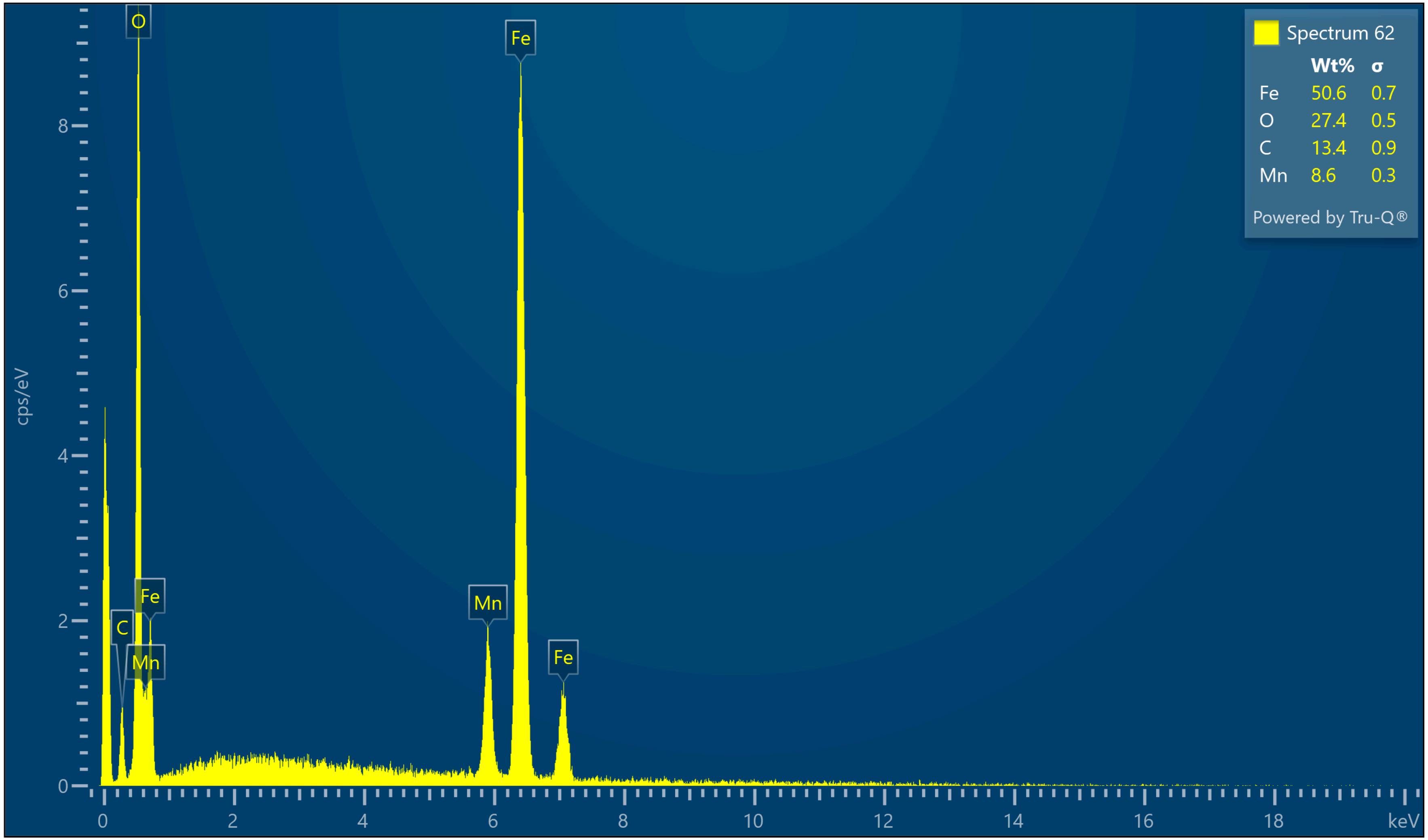

cps/eV
0
2
4
6
8
O
Fe
C
Fe
Mn
Mn
Fe
0
2
4
6
8
10
12
14
16
18
keV
Spectrum 62
Wt% σ
Fe 50.6 0.7
O 27.4 0.5
C 13.4 0.9
Mn 8.6 0.3
Powered by Tru-Q®

Figure S30

Table S30. Spectrum 62									
Element	Signal Type	Line	Apparent Concentration	k Ratio	Wt%	Wt% Sigma	Standard Name	Factory Standard	Standardization Date
C	EDS	K series	3.65	0.03653	13.36	0.88	C Vit	Yes	
O	EDS	K series	39.31	0.13227	27.43	0.54	SiO2	Yes	
Mn	EDS	K series	8.40	0.08397	8.59	0.30	Mn	Yes	
Fe	EDS	K series	50.45	0.50448	50.62	0.69	Fe	Yes	
Total					100.00				

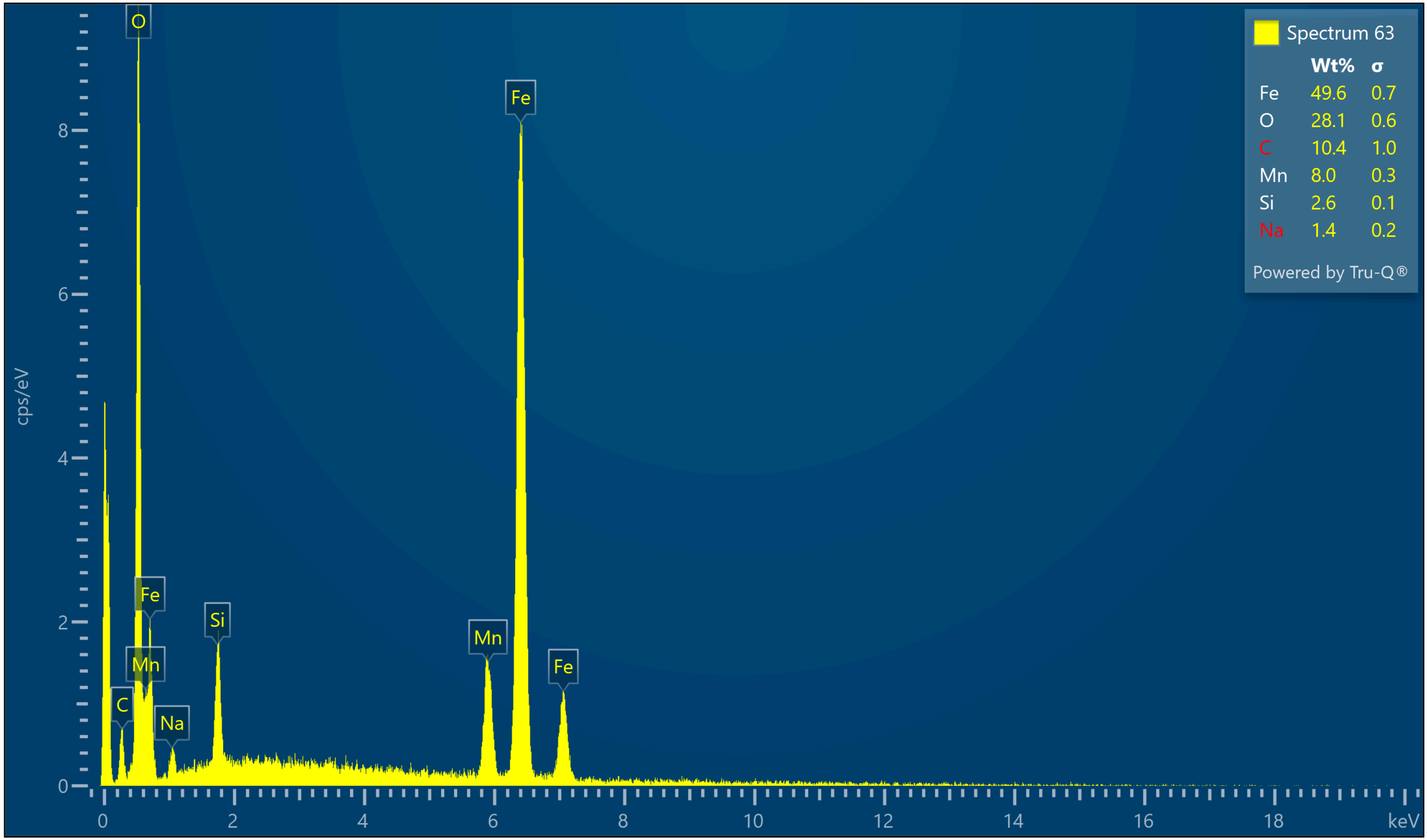
Spectrum 63
Wt% σ
Fe 49.6 0.7
O 28.1 0.6
C 10.4 1.0
Mn 8.0 0.3
Si 2.6 0.1
Na 1.4 0.2
Powered by Tru-Q®
cps/eV
keV
O
Fe
Fe
Mn
C
Na
Si
Mn
Fe
0
2
4
6
8
10
12
14
16
18

Figure S31

Table S31. Spectrum 63

Element	Signal Type	Line	Apparent Concentration	k Ratio	Wt%	Wt% Sigma	Standard Name	Factory Standard	Standardization Date
C	EDS	K series	2.46	0.02460	10.40	0.97	C Vit	Yes	
O	EDS	K series	39.73	0.13369	28.05	0.57	SiO2	Yes	
Na	EDS	K series	0.67	0.00284	1.36	0.21	Albite	Yes	
Si	EDS	K series	1.92	0.01520	2.55	0.14	SiO2	Yes	
Mn	EDS	K series	7.50	0.07500	8.01	0.30	Mn	Yes	
Fe	EDS	K series	47.35	0.47351	49.62	0.72	Fe	Yes	
Total					100.00				

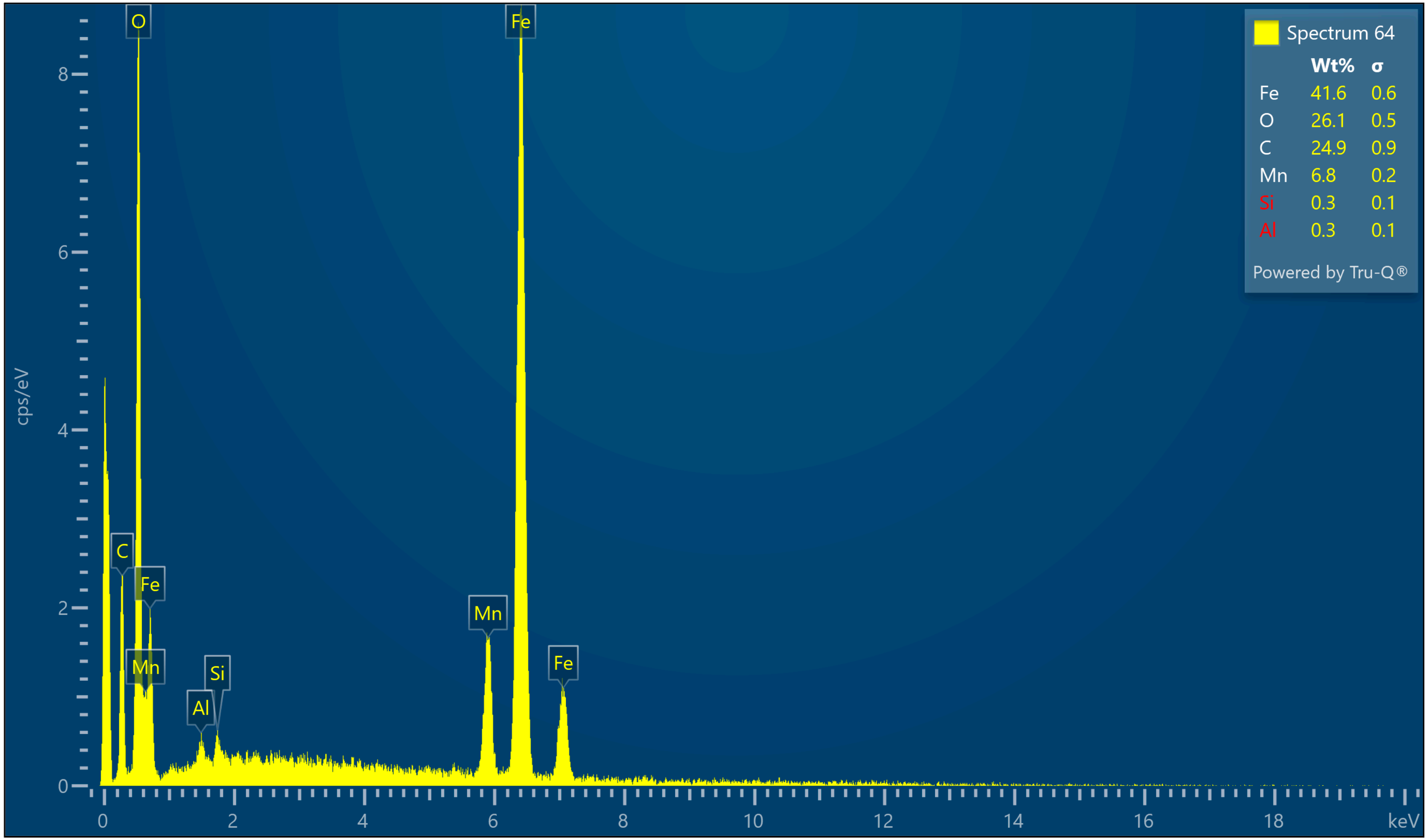

Figure S32

OXFORD INSTRUMENTS

Table S32. Spectrum 64

Element	Signal Type	Line	Apparent Concentration	k Ratio	Wt%	Wt% Sigma	Standard Name	Factory Standard	Standardization Date
C	EDS	K series	9.27	0.09271	24.86	0.87	C Vit	Yes	
O	EDS	K series	35.74	0.12027	26.11	0.54	SiO2	Yes	
Al	EDS	K series	0.27	0.00194	0.32	0.08	Al2O3	Yes	
Si	EDS	K series	0.35	0.00276	0.34	0.07	SiO2	Yes	
Mn	EDS	K series	8.01	0.08013	6.81	0.25	Mn	Yes	
Fe	EDS	K series	49.74	0.49735	41.56	0.62	Fe	Yes	
Total					100.00				

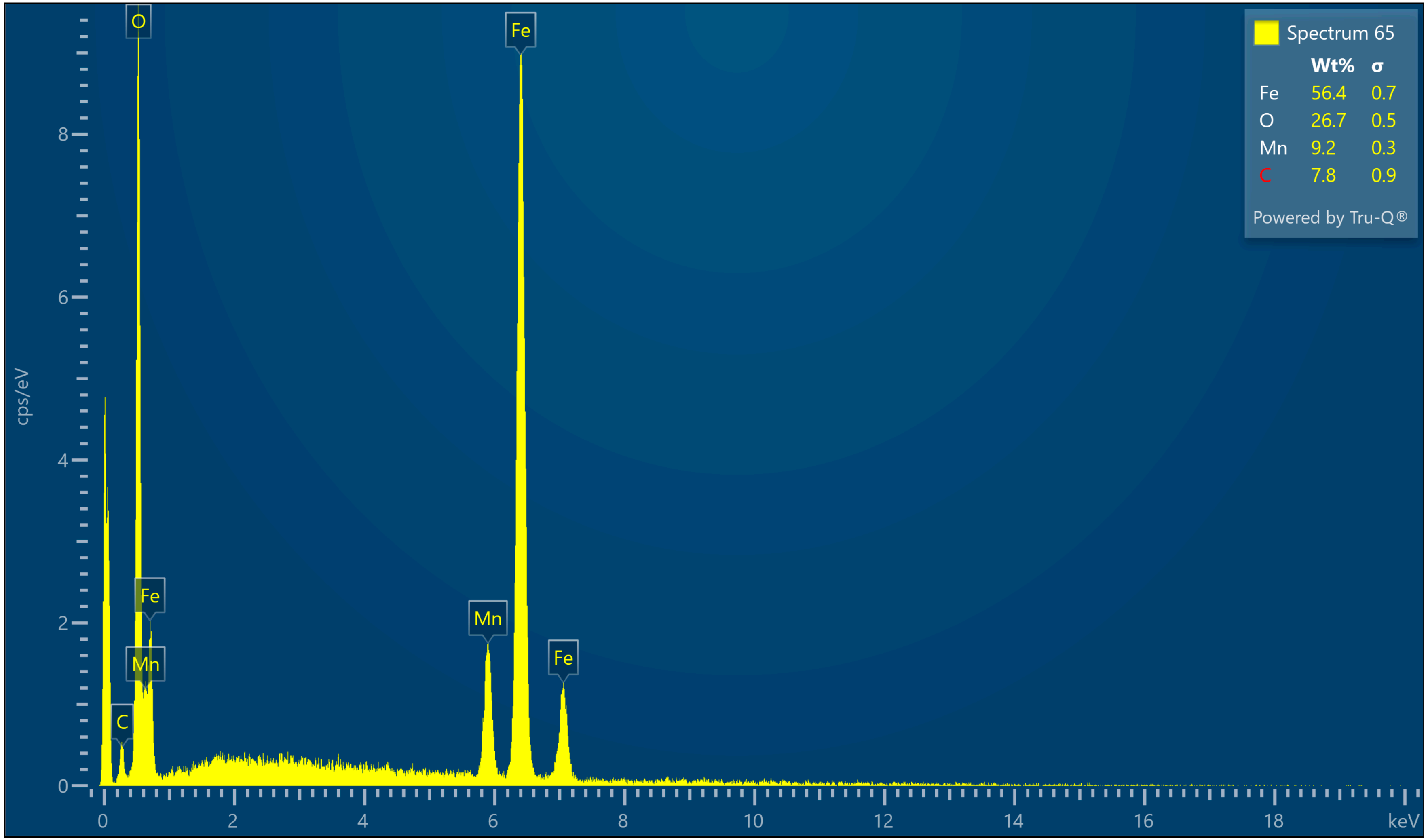
Spectrum 65
Wt% σ
Fe 56.4 0.7
O 26.7 0.5
Mn 9.2 0.3
C 7.8 0.9
Powered by Tru-Q®
cps/eV
keV
O
Fe
Fe
Mn
Mn
C
Fe
0
2
4
6
8
10
12
14
16
18

Figure S33

Table S33. Spectrum 65

Element	Signal Type	Line	Apparent Concentration	k Ratio	Wt%	Wt% Sigma	Standard Name	Factory Standard	Standardization Date
C	EDS	K series	1.81	0.01812	7.76	0.88	C Vit	Yes	
O	EDS	K series	39.21	0.13195	26.67	0.53	SiO2	Yes	
Mn	EDS	K series	8.28	0.08277	9.20	0.32	Mn	Yes	
Fe	EDS	K series	51.72	0.51721	56.37	0.72	Fe	Yes	
Total					100.00				

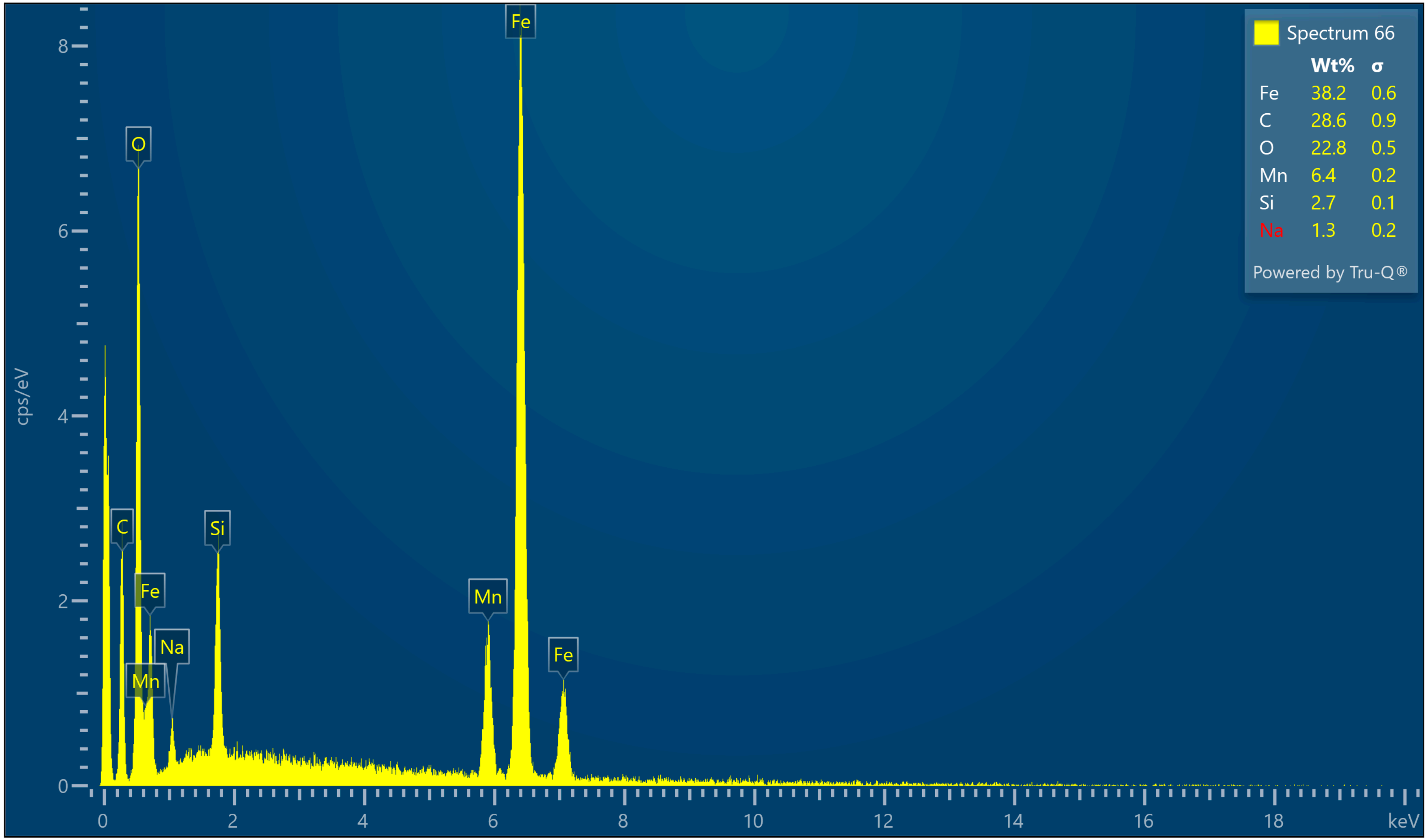

Figure S34

OXFORD INSTRUMENTS

Table S34. Spectrum 66

Element	Signal Type	Line	Apparent Concentration	k Ratio	Wt%	Wt% Sigma	Standard Name	Factory Standard	Standardization Date
C	EDS	K series	10.46	0.10464	28.63	0.86	C Vit	Yes	
O	EDS	K series	28.86	0.09711	22.80	0.53	SiO2	Yes	
Na	EDS	K series	0.98	0.00412	1.30	0.16	Albite	Yes	
Si	EDS	K series	2.87	0.02273	2.67	0.12	SiO2	Yes	
Mn	EDS	K series	7.73	0.07729	6.41	0.24	Mn	Yes	
Fe	EDS	K series	46.84	0.46842	38.19	0.60	Fe	Yes	
Total					100.00				

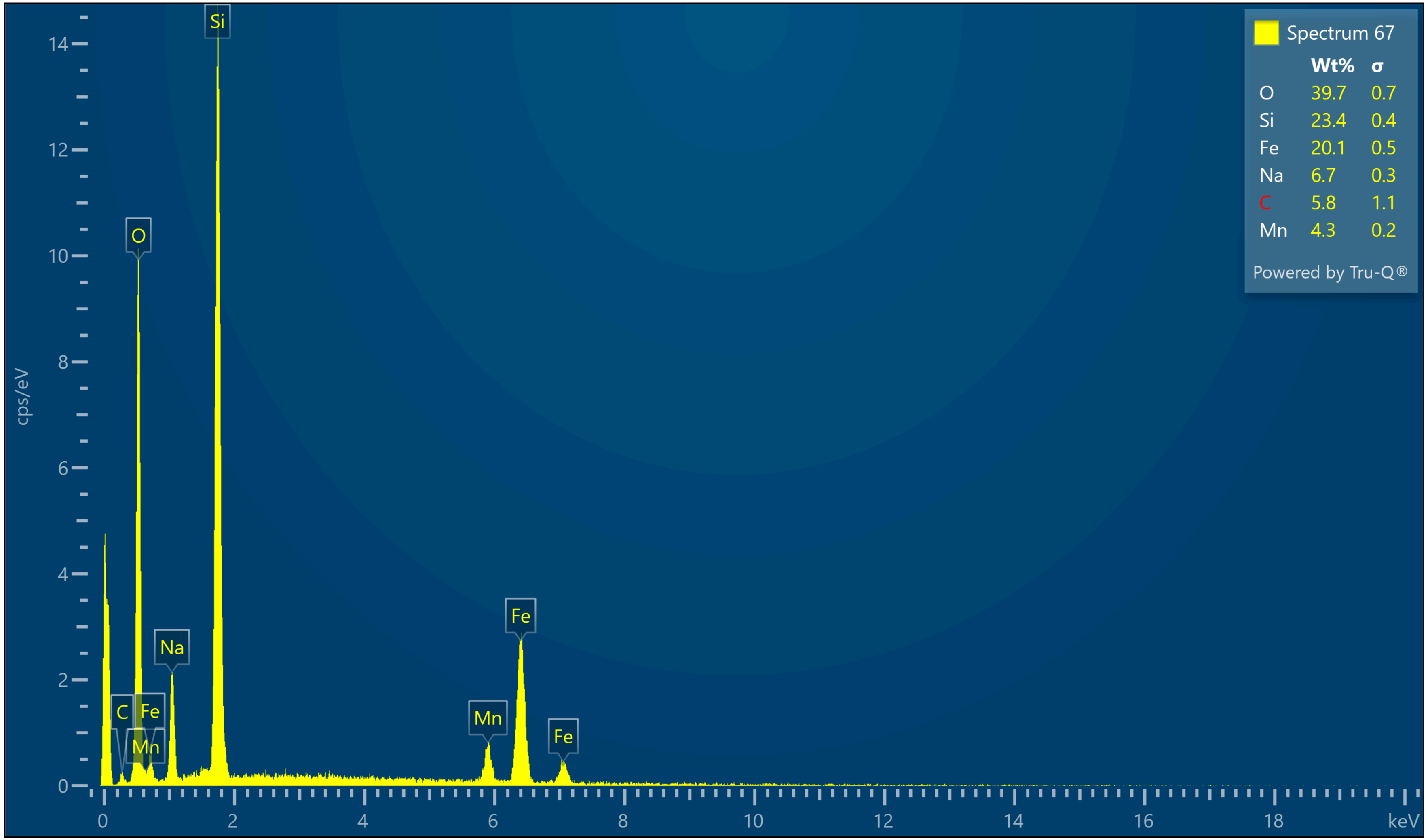
Spectrum 67
Wt% σ
O 39.7 0.7
Si 23.4 0.4
Fe 20.1 0.5
Na 6.7 0.3
C 5.8 1.1
Mn 4.3 0.2
Powered by Tru-Q®
cps/eV
keV
Si
O
Na
C
Fe
Mn
Mn
Fe
Fe

Figure S35

Table S35. Spectrum 67

Element	Signal Type	Line	Apparent Concentration	k Ratio	Wt%	Wt% Sigma	Standard Name	Factory Standard	Standardization Date
C	EDS	K series	0.80	0.00803	5.79	1.11	C Vit	Yes	
O	EDS	K series	46.13	0.15522	39.73	0.70	SiO2	Yes	
Na	EDS	K series	4.58	0.01935	6.70	0.27	Albite	Yes	
Si	EDS	K series	19.41	0.15378	23.44	0.43	SiO2	Yes	
Mn	EDS	K series	3.62	0.03625	4.29	0.24	Mn	Yes	
Fe	EDS	K series	17.29	0.17291	20.06	0.47	Fe	Yes	
Total					100.00				

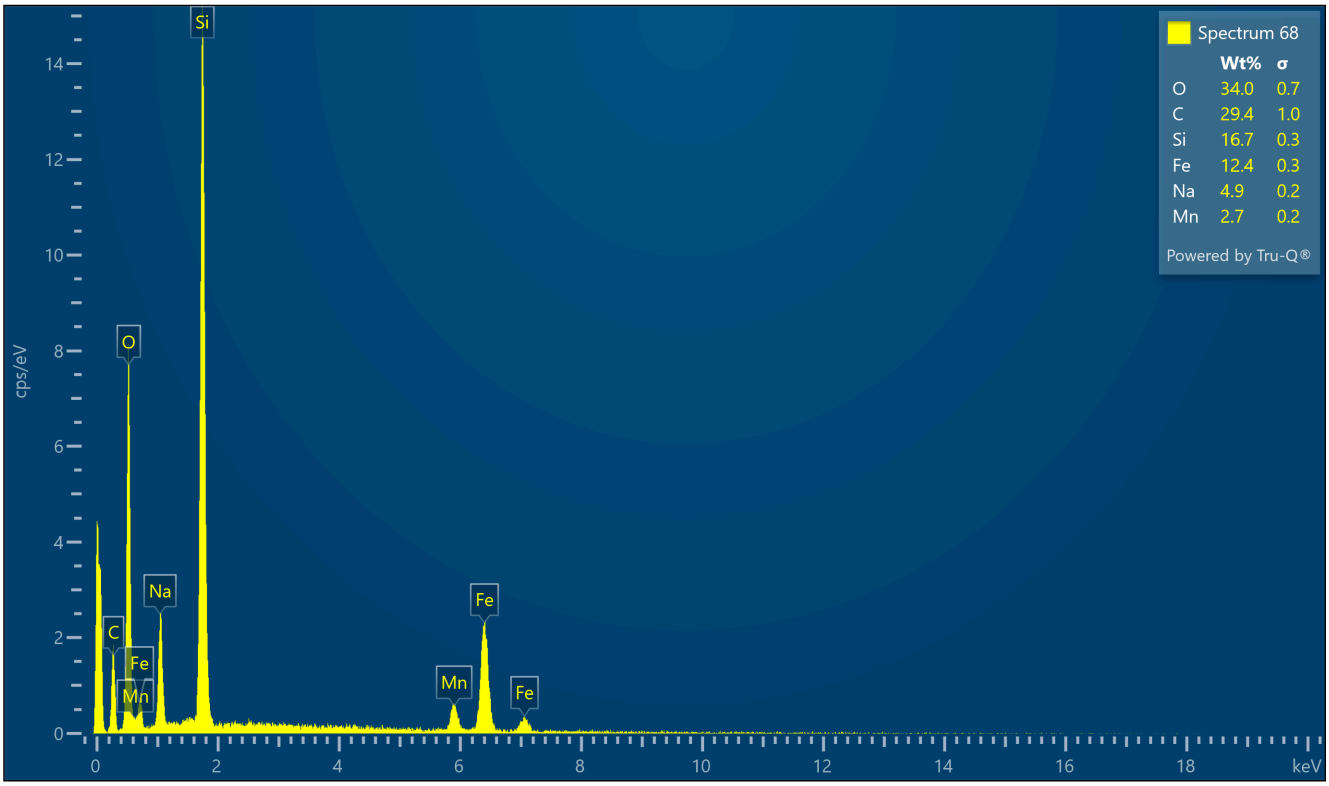

Figure S36

Table S36. Spectrum 68

Element	Signal Type	Line	Apparent Concentration	k Ratio	Wt%	Wt% Sigma	Standard Name	Factory Standard	Standardization Date
C	EDS	K series	7.65	0.07645	29.37	1.04	C Vit	Yes	
O	EDS	K series	36.61	0.12319	33.99	0.69	SiO2	Yes	
Na	EDS	K series	5.35	0.02259	4.91	0.20	Albite	Yes	
Si	EDS	K series	20.72	0.16415	16.72	0.33	SiO2	Yes	
Mn	EDS	K series	3.07	0.03074	2.66	0.17	Mn	Yes	
Fe	EDS	K series	14.53	0.14525	12.35	0.34	Fe	Yes	
Total					100.00				

OXFORD INSTRUMENTS

Sample 20FR

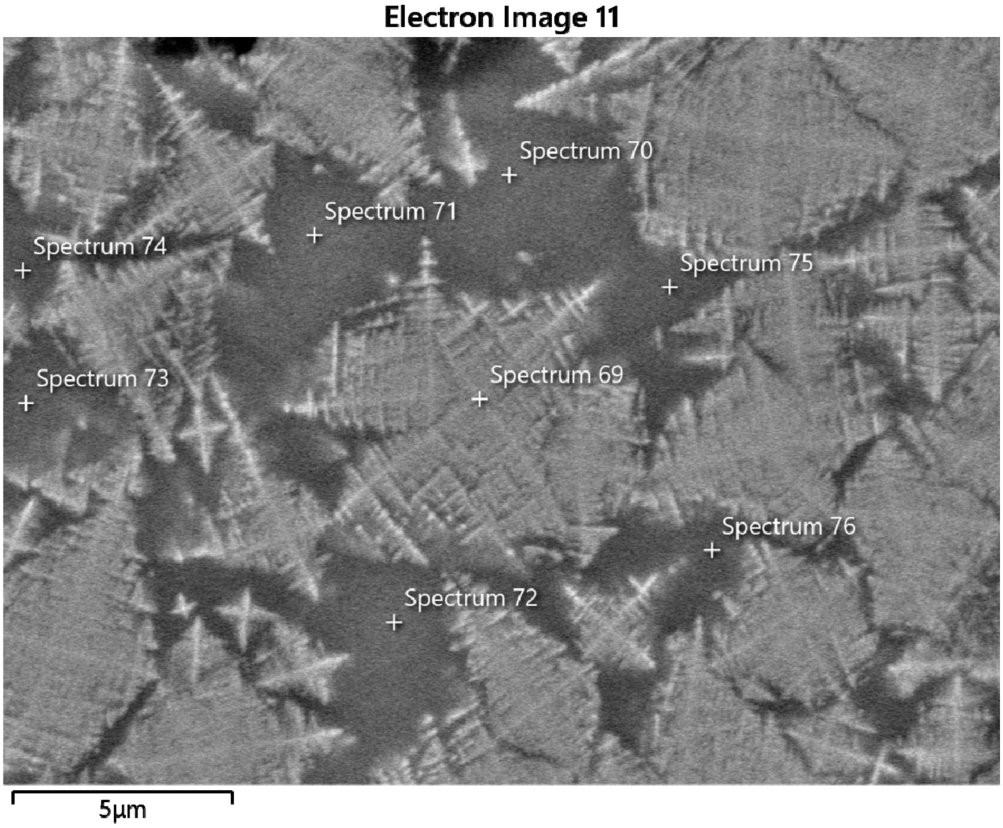

Figure S37

Table S37

Element	69	70	71	72	73	74	75	76
Mn	3,03	5,77	6,06	5,76	5,22	3,96	5,71	5,16
Fe	24,47	20,24	20,48	18,71	21,54	15,39	19,29	21,05
Ratio Fe/Mn	**8,1**	3,5	3,4	3,2	4,1	3,9	3,4	4,1
	**Dendrite**	Glass	Glass	Glass	Glass	Glass	Glass	Glass

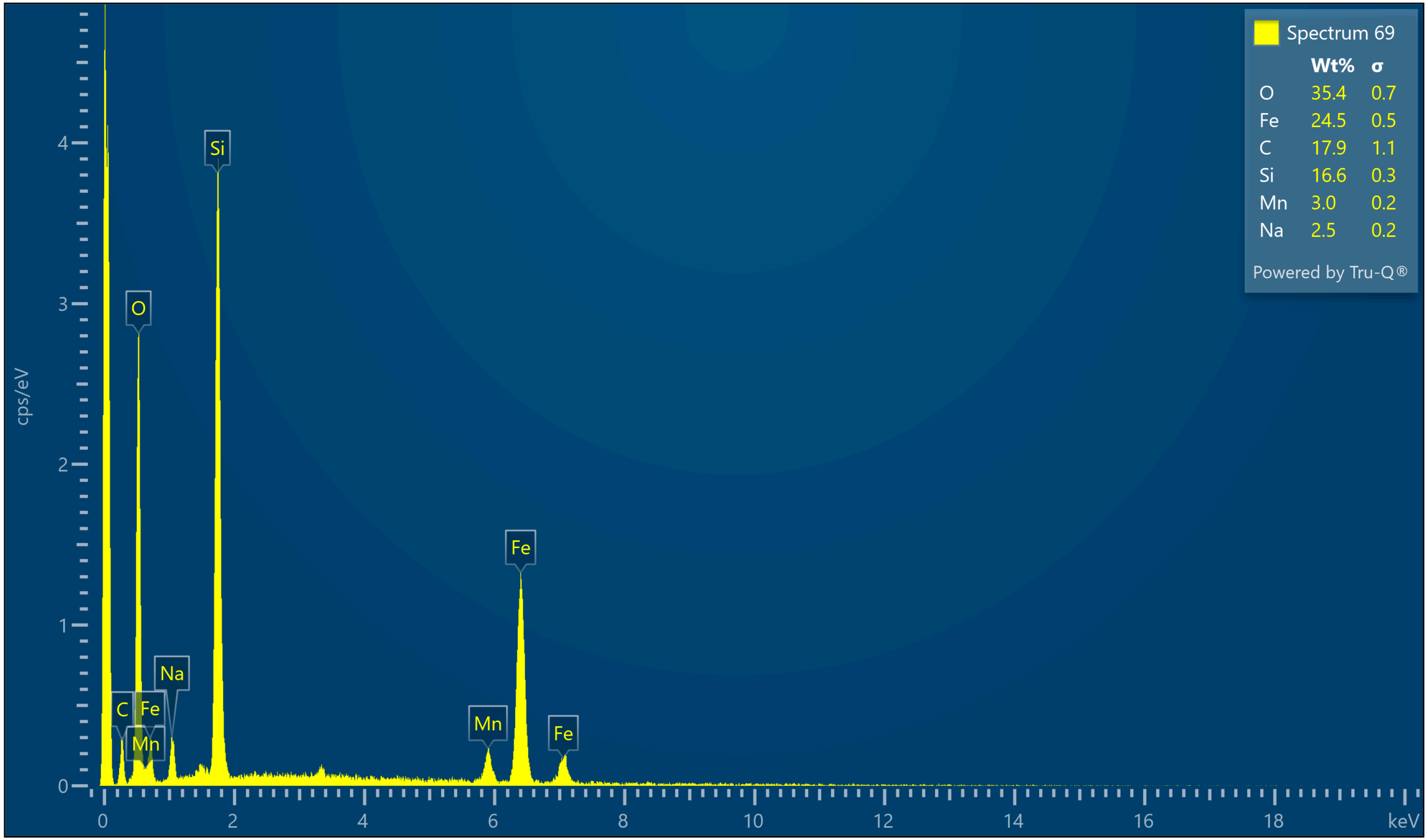

Figure S38

OXFORD INSTRUMENTS

Table S38. Spectrum 69									
Element	Signal Type	Line	Apparent Concentration	k Ratio	Wt%	Wt% Sigma	Standard Name	Factory Standard	Standardization Date
C	EDS	K series	3.04	0.03041	17.93	1.10	C Vit	Yes	
O	EDS	K series	33.64	0.11322	35.44	0.68	SiO2	Yes	
Na	EDS	K series	1.57	0.00663	2.50	0.17	Albite	Yes	
Si	EDS	K series	13.49	0.10687	16.62	0.33	SiO2	Yes	
Mn	EDS	K series	2.50	0.02497	3.03	0.20	Mn	Yes	
Fe	EDS	K series	20.51	0.20514	24.47	0.51	Fe	Yes	
Total					100.00				

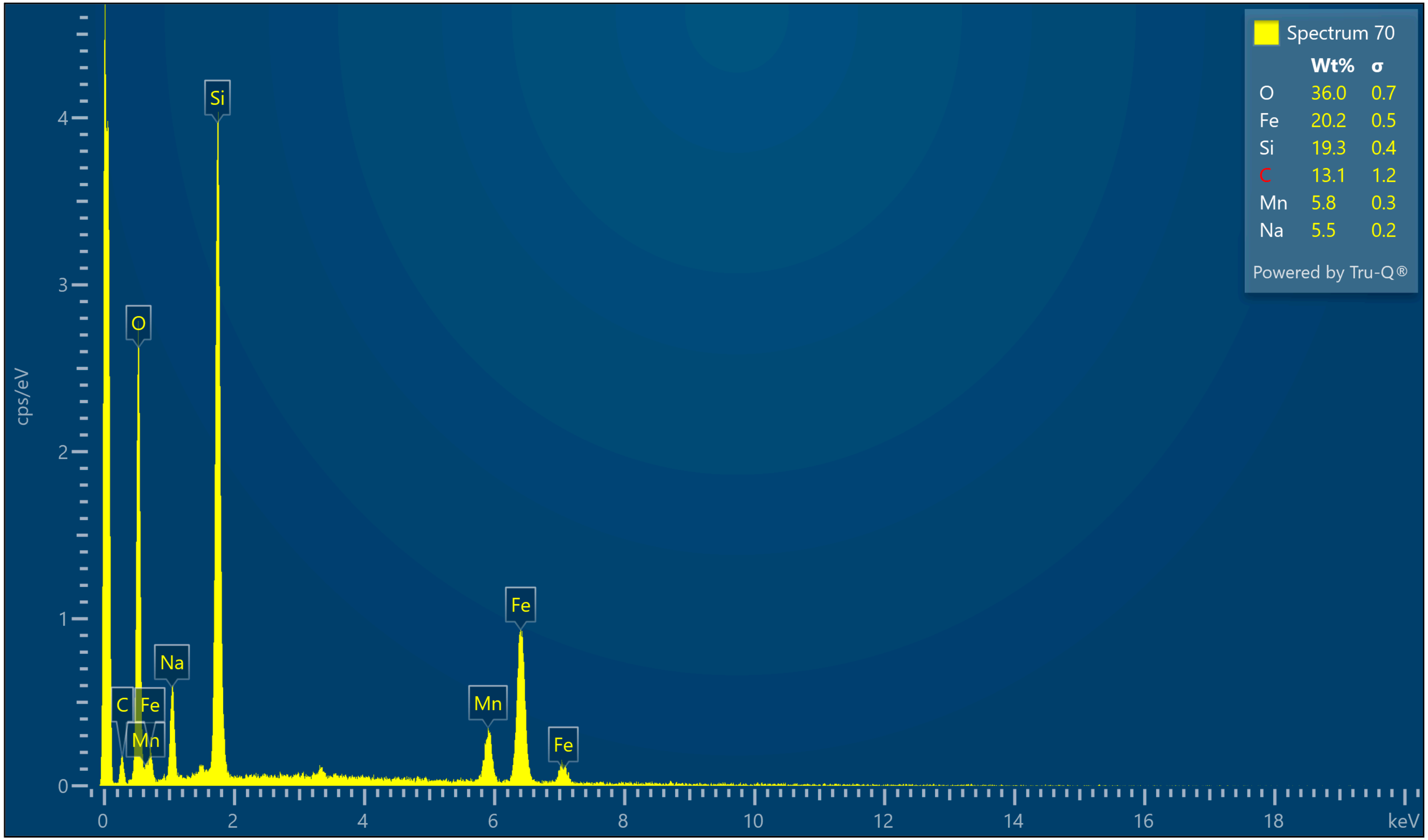
Spectrum 70
Wt% σ
O 36.0 0.7
Fe 20.2 0.5
Si 19.3 0.4
C 13.1 1.2
Mn 5.8 0.3
Na 5.5 0.2
Powered by Tru-Q®
cps/eV
keV
Si
O
Na
C
Fe
Mn
Fe
Mn
Fe

Figure S39

Table S39. Spectrum 70									
Element	Signal Type	Line	Apparent Concentration	k Ratio	Wt%	Wt% Sigma	Standard Name	Factory Standard	Standardization Date
C	EDS	K series	1.84	0.01836	13.15	1.18	C Vit	Yes	
O	EDS	K series	33.24	0.11185	36.03	0.70	SiO2	Yes	
Na	EDS	K series	3.31	0.01399	5.55	0.25	Albite	Yes	
Si	EDS	K series	14.19	0.11241	19.27	0.38	SiO2	Yes	
Mn	EDS	K series	4.33	0.04332	5.77	0.26	Mn	Yes	
Fe	EDS	K series	15.50	0.15496	20.24	0.48	Fe	Yes	
Total					100.00				

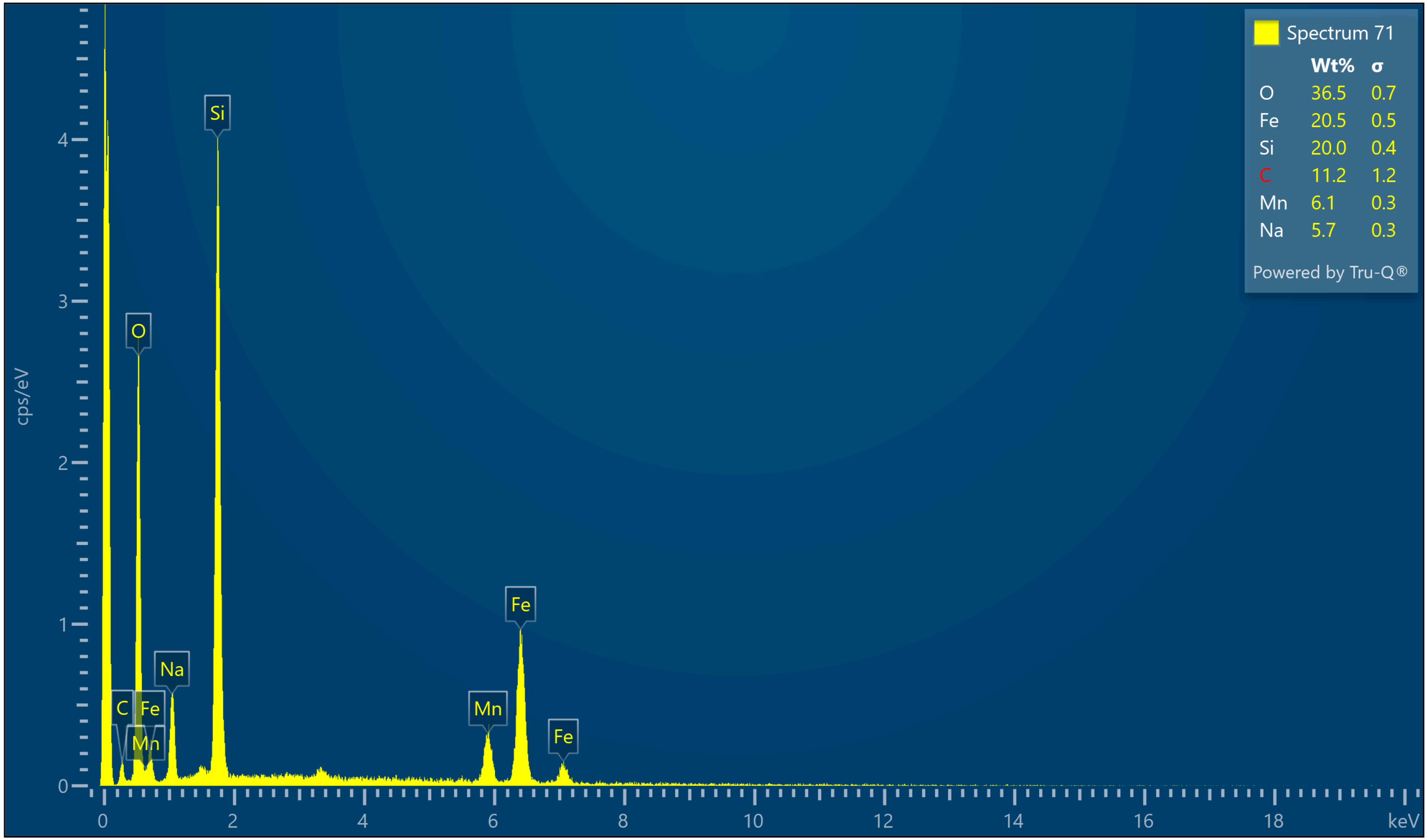
Spectrum 71
Wt% σ
O 36.5 0.7
Fe 20.5 0.5
Si 20.0 0.4
C 11.2 1.2
Mn 6.1 0.3
Na 5.7 0.3
Powered by Tru-Q®
cps/eV
keV
Si
O
Na
C
Fe
Mn
Fe
Mn
Fe

Figure S40

Table S40. Spectrum 71									
Element	Signal Type	Line	Apparent Concentration	k Ratio	Wt%	Wt% Sigma	Standard Name	Factory Standard	Standardization Date
C	EDS	K series	1.46	0.01464	11.24	1.23	C Vit	Yes	
O	EDS	K series	33.29	0.11201	36.46	0.72	SiO2	Yes	
Na	EDS	K series	3.26	0.01378	5.73	0.26	Albite	Yes	
Si	EDS	K series	14.09	0.11168	20.03	0.40	SiO2	Yes	
Mn	EDS	K series	4.37	0.04371	6.06	0.27	Mn	Yes	
Fe	EDS	K series	15.06	0.15061	20.48	0.49	Fe	Yes	
Total					100.00				

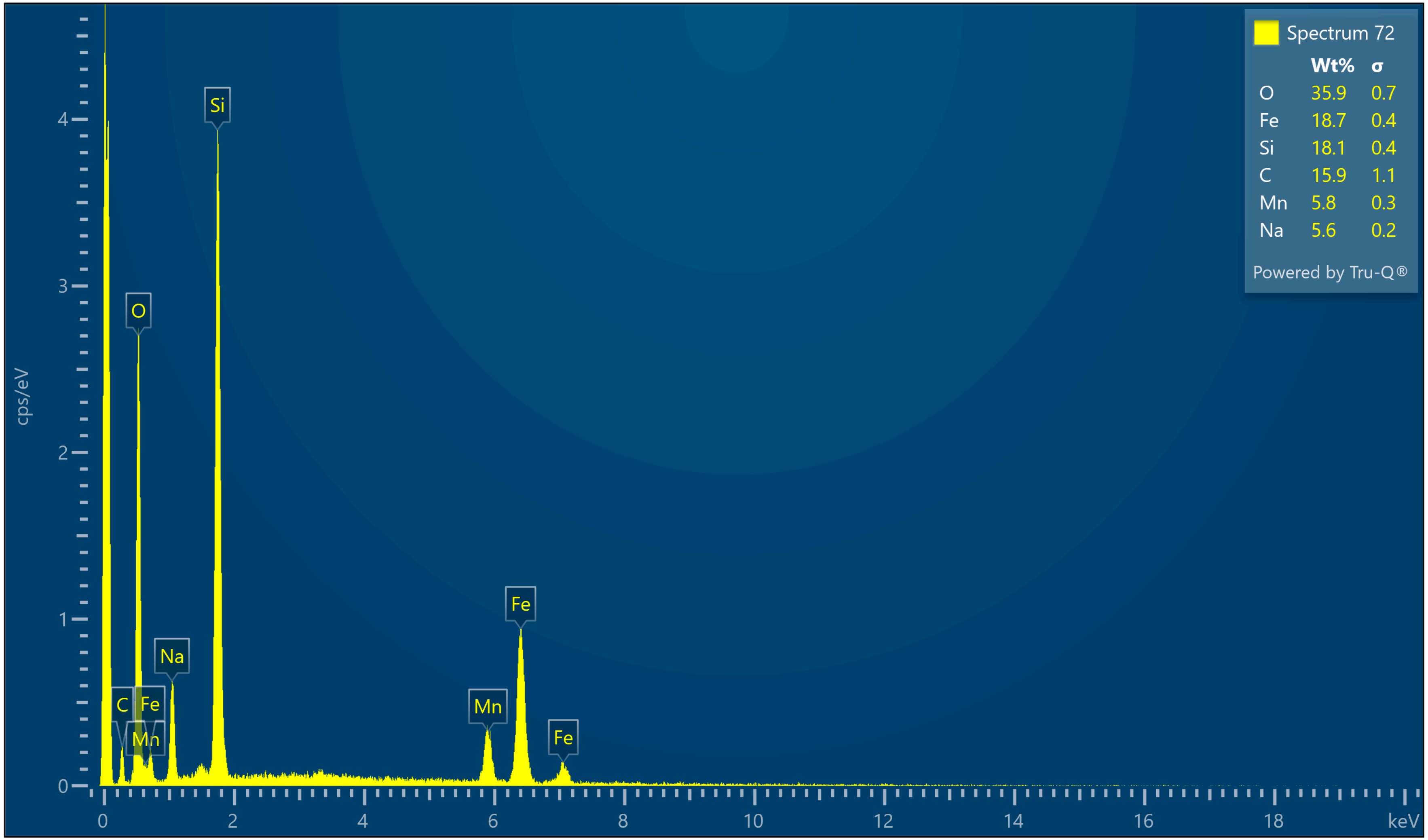

Spectrum 72
Wt% σ
O 35.9 0.7
Fe 18.7 0.4
Si 18.1 0.4
C 15.9 1.1
Mn 5.8 0.3
Na 5.6 0.2
Powered by Tru-Q®
cps/eV
keV
Si
O
Na
C
Fe
Mn
Fe
Mn
Fe

Figure S41

Table S41. Spectrum 72									
Element	Signal Type	Line	Apparent Concentration	k Ratio	Wt%	Wt% Sigma	Standard Name	Factory Standard	Standardization Date
C	EDS	K series	2.42	0.02424	15.86	1.11	C Vit	Yes	
O	EDS	K series	33.36	0.11227	35.93	0.69	SiO2	Yes	
Na	EDS	K series	3.60	0.01519	5.64	0.24	Albite	Yes	
Si	EDS	K series	14.07	0.11150	18.10	0.35	SiO2	Yes	
Mn	EDS	K series	4.53	0.04526	5.76	0.25	Mn	Yes	
Fe	EDS	K series	14.97	0.14967	18.71	0.45	Fe	Yes	
Total					100.00				

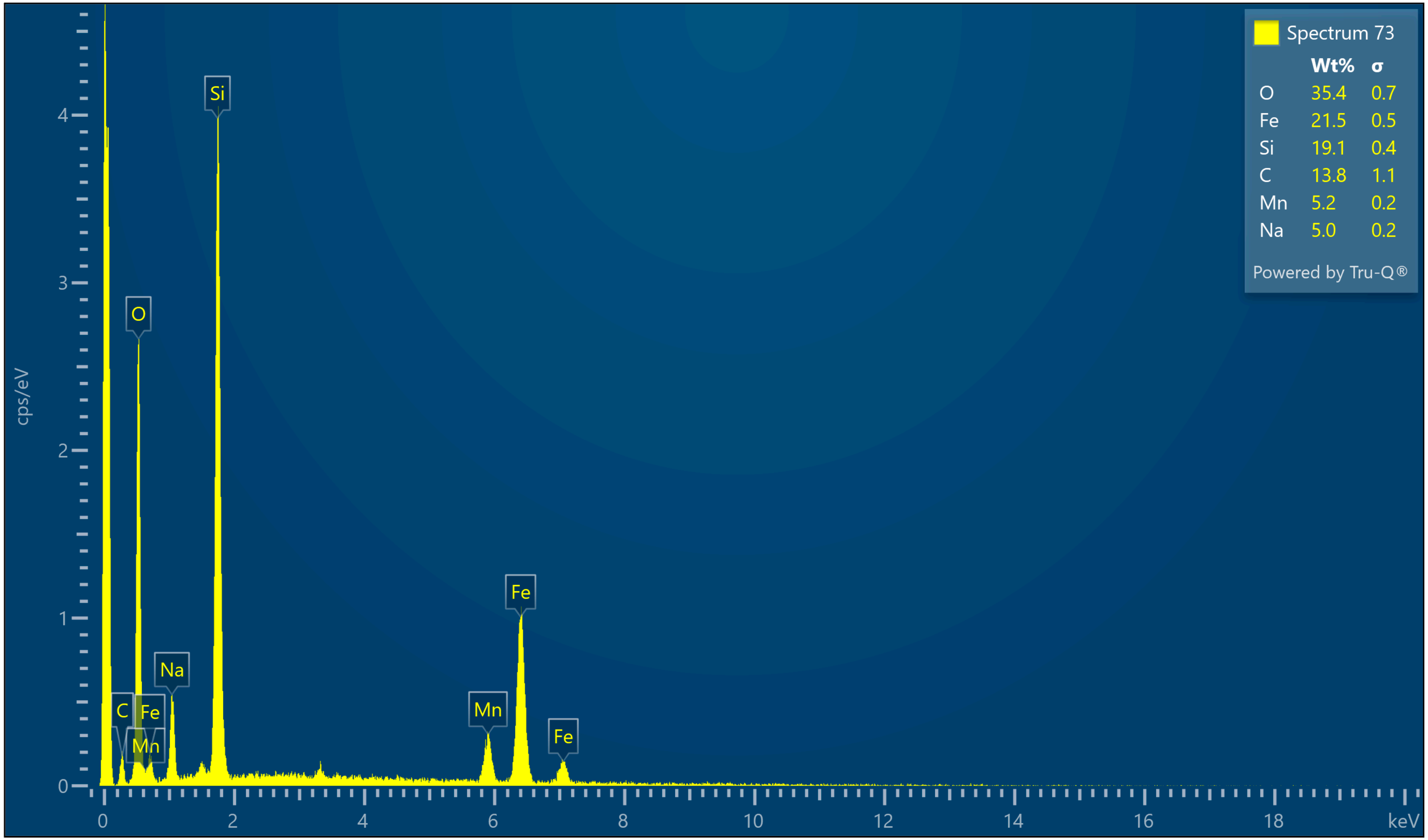
Spectrum 73
Wt% σ
O 35.4 0.7
Fe 21.5 0.5
Si 19.1 0.4
C 13.8 1.1
Mn 5.2 0.2
Na 5.0 0.2
Powered by Tru-Q®
cps/eV
keV
Si
O
Na
C
Fe
Mn
Mn
Fe
Fe

Figure S42

Table S42. Spectrum 73									
Element	Signal Type	Line	Apparent Concentration	k Ratio	Wt%	Wt% Sigma	Standard Name	Factory Standard	Standardization Date
C	EDS	K series	1.96	0.01964	13.78	1.15	C Vit	Yes	
O	EDS	K series	32.64	0.10984	35.38	0.69	SiO2	Yes	
Na	EDS	K series	2.98	0.01257	4.97	0.24	Albite	Yes	
Si	EDS	K series	14.24	0.11283	19.10	0.37	SiO2	Yes	
Mn	EDS	K series	3.98	0.03976	5.22	0.25	Mn	Yes	
Fe	EDS	K series	16.70	0.16702	21.54	0.49	Fe	Yes	
Total					100.00				

OXFORD INSTRUMENTS

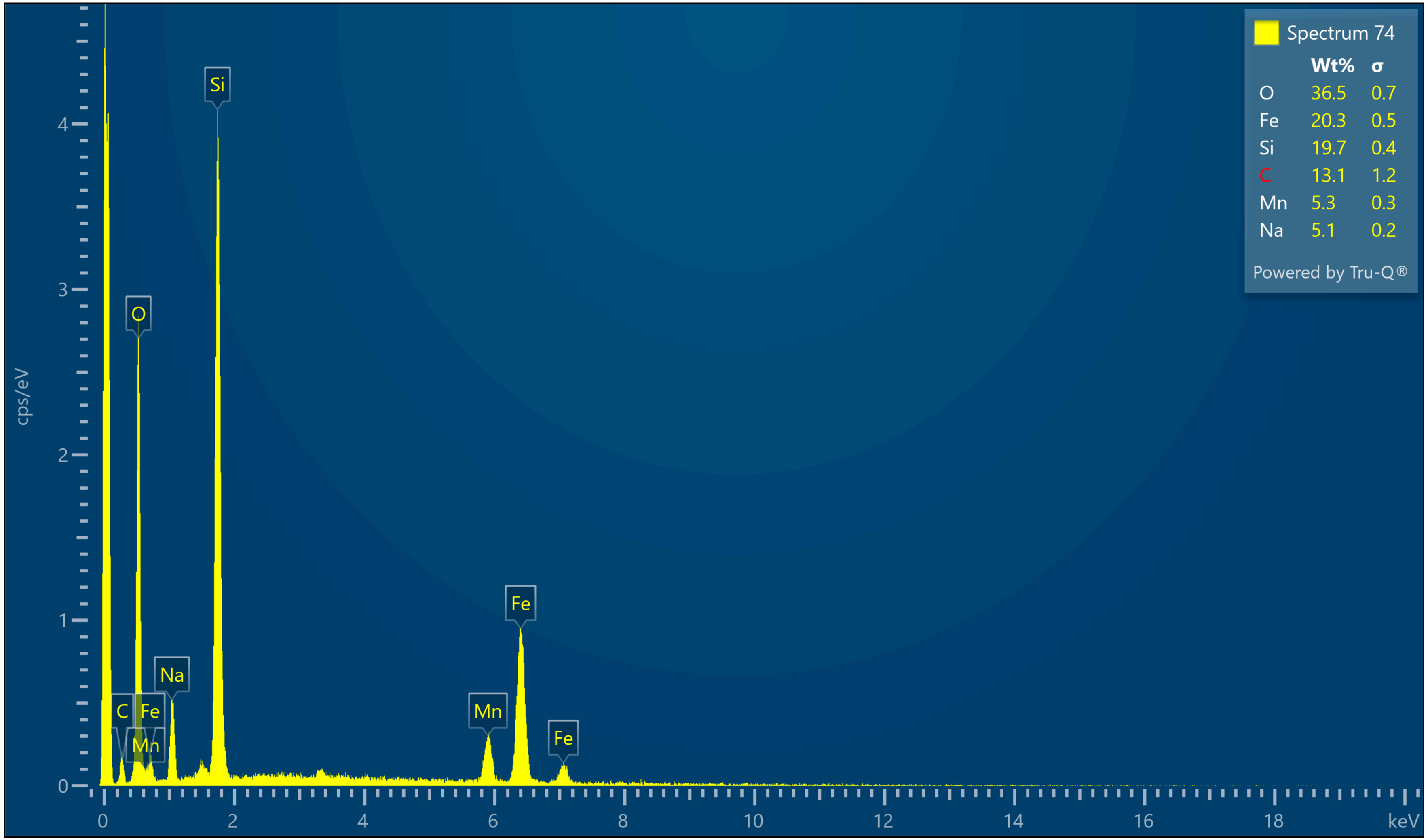

Figure S43

OXFORD INSTRUMENTS

Table 43. Spectrum 74									
Element	Signal Type	Line	Apparent Concentration	k Ratio	Wt%	Wt% Sigma	Standard Name	Factory Standard	Standardization Date
C	EDS	K series	1.80	0.01797	13.08	1.20	C Vit	Yes	
O	EDS	K series	33.28	0.11198	36.53	0.71	SiO2	Yes	
Na	EDS	K series	2.98	0.01260	5.05	0.23	Albite	Yes	
Si	EDS	K series	14.37	0.11389	19.66	0.39	SiO2	Yes	
Mn	EDS	K series	3.96	0.03956	5.33	0.26	Mn	Yes	
Fe	EDS	K series	15.39	0.15391	20.35	0.49	Fe	Yes	
Total					100.00				

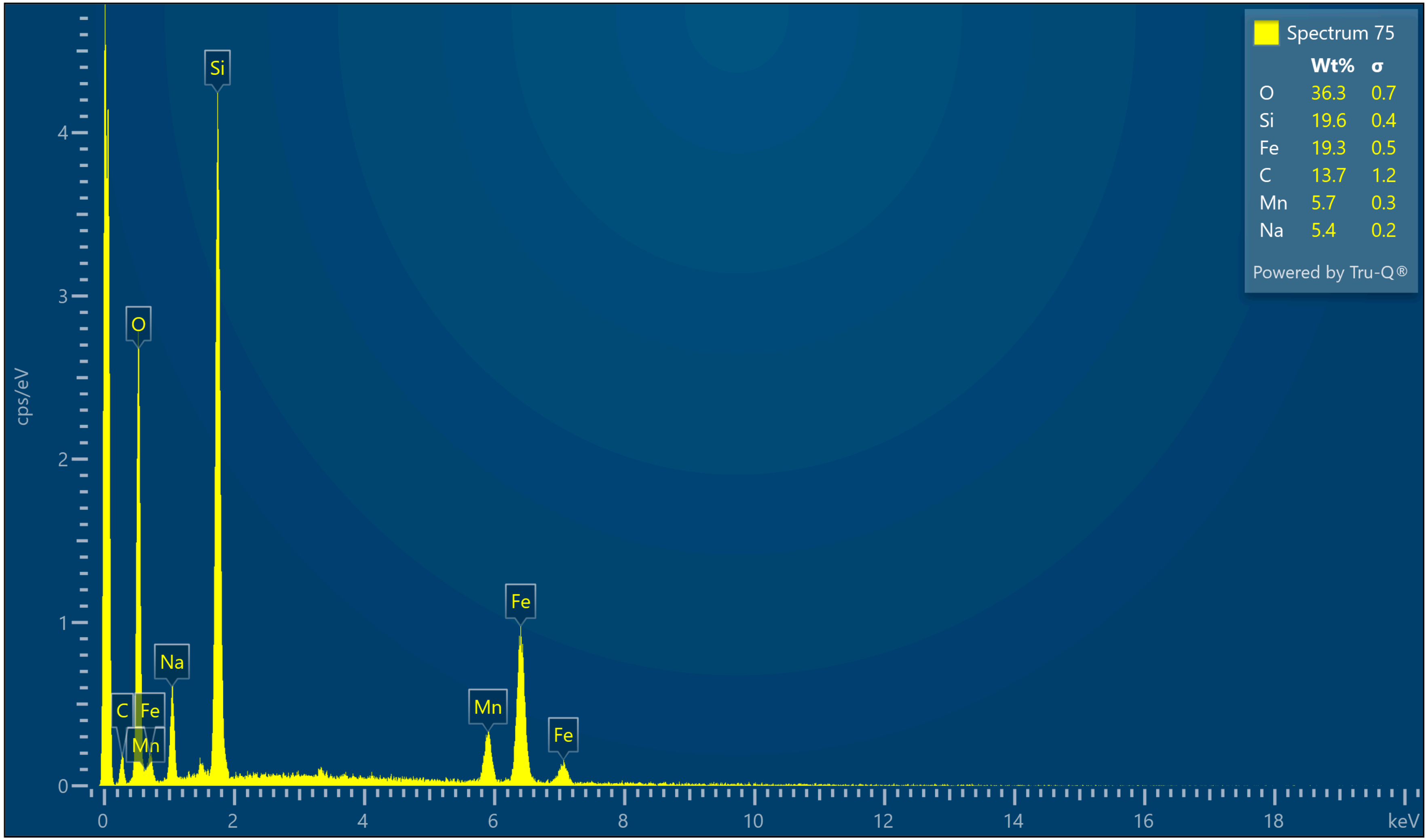
Spectrum 75
Wt% σ
O 36.3 0.7
Si 19.6 0.4
Fe 19.3 0.5
C 13.7 1.2
Mn 5.7 0.3
Na 5.4 0.2
Powered by Tru-Q®
cps/eV
keV
Si
O
Na
C
Fe
Mn
Fe
Mn
Fe

Figure S44

Table S44. Spectrum 75									
Element	Signal Type	Line	Apparent Concentration	k Ratio	Wt%	Wt% Sigma	Standard Name	Factory Standard	Standardization Date
C	EDS	K series	1.91	0.01911	13.73	1.18	C Vit	Yes	
O	EDS	K series	32.94	0.11084	36.31	0.71	SiO2	Yes	
Na	EDS	K series	3.25	0.01370	5.38	0.24	Albite	Yes	
Si	EDS	K series	14.49	0.11486	19.59	0.39	SiO2	Yes	
Mn	EDS	K series	4.27	0.04275	5.71	0.26	Mn	Yes	
Fe	EDS	K series	14.71	0.14711	19.29	0.47	Fe	Yes	
Total					100.00				

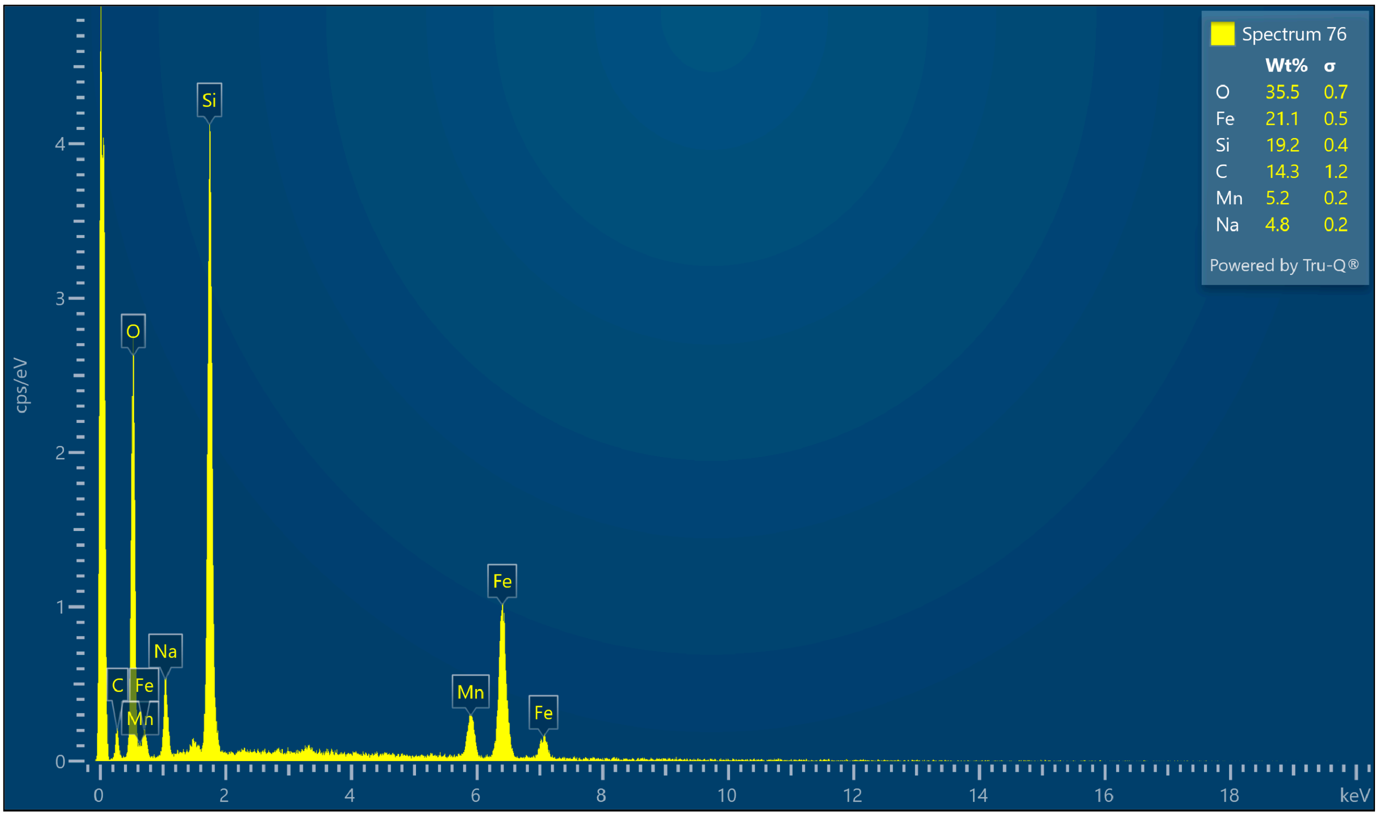

Figure S45

Table S45. Spectrum 76

Element	Signal Type	Line	Apparent Concentration	k Ratio	Wt%	Wt% Sigma	Standard Name	Factory Standard	Standardization Date
C	EDS	K series	2.05	0.02045	14.28	1.18	C Vit	Yes	
O	EDS	K series	32.49	0.10934	35.49	0.70	SiO2	Yes	
Na	EDS	K series	2.89	0.01221	4.79	0.23	Albite	Yes	
Si	EDS	K series	14.46	0.11460	19.24	0.38	SiO2	Yes	
Mn	EDS	K series	3.94	0.03938	5.16	0.25	Mn	Yes	
Fe	EDS	K series	16.37	0.16371	21.05	0.49	Fe	Yes	
Total					100.00				

Sample 25F

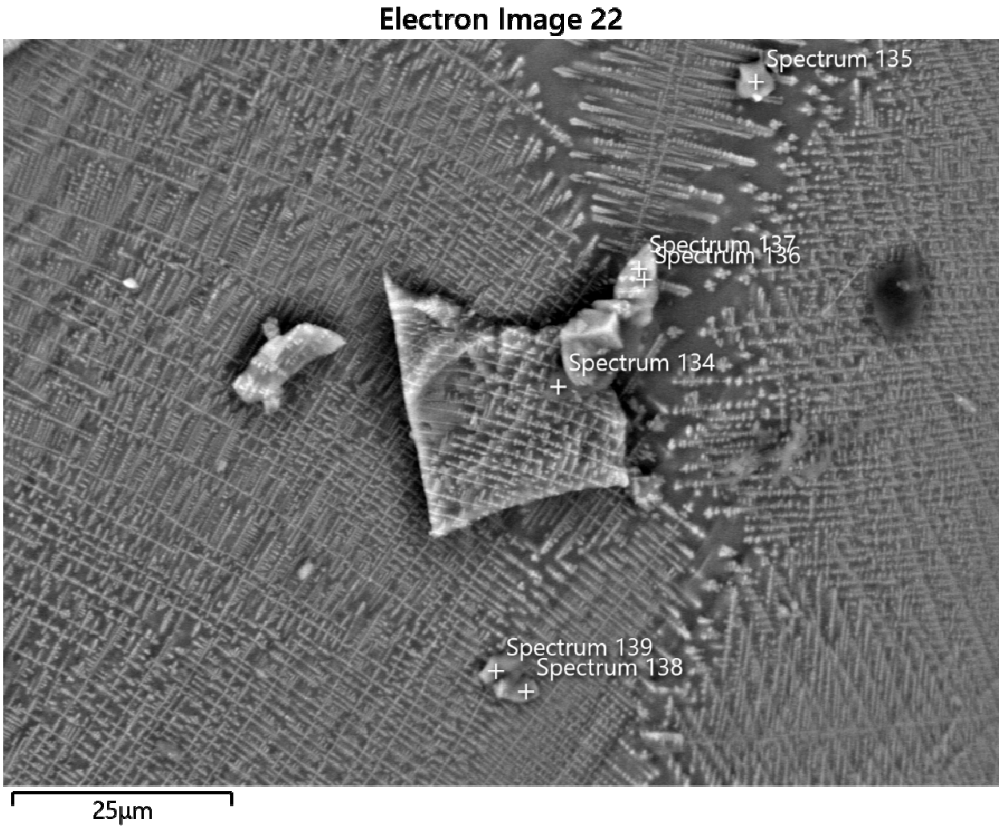

Figure S46

Table S46

	Fe	Mn	Ratio Fe/Mn		Ratio Fe/Mn end
134	13,18	2,34	5,632479	dendrites region	6
135	11,94	1,78	6,707865	dendrites region	7
136	14,65	2,16	6,782407	dendrites region	7
137	27,61	3,69	7,482385	dendrites region	7
138	28,42	4,39	6,473804	dendrites region	6
139	21,78	3,37	6,462908	dendrites region	6

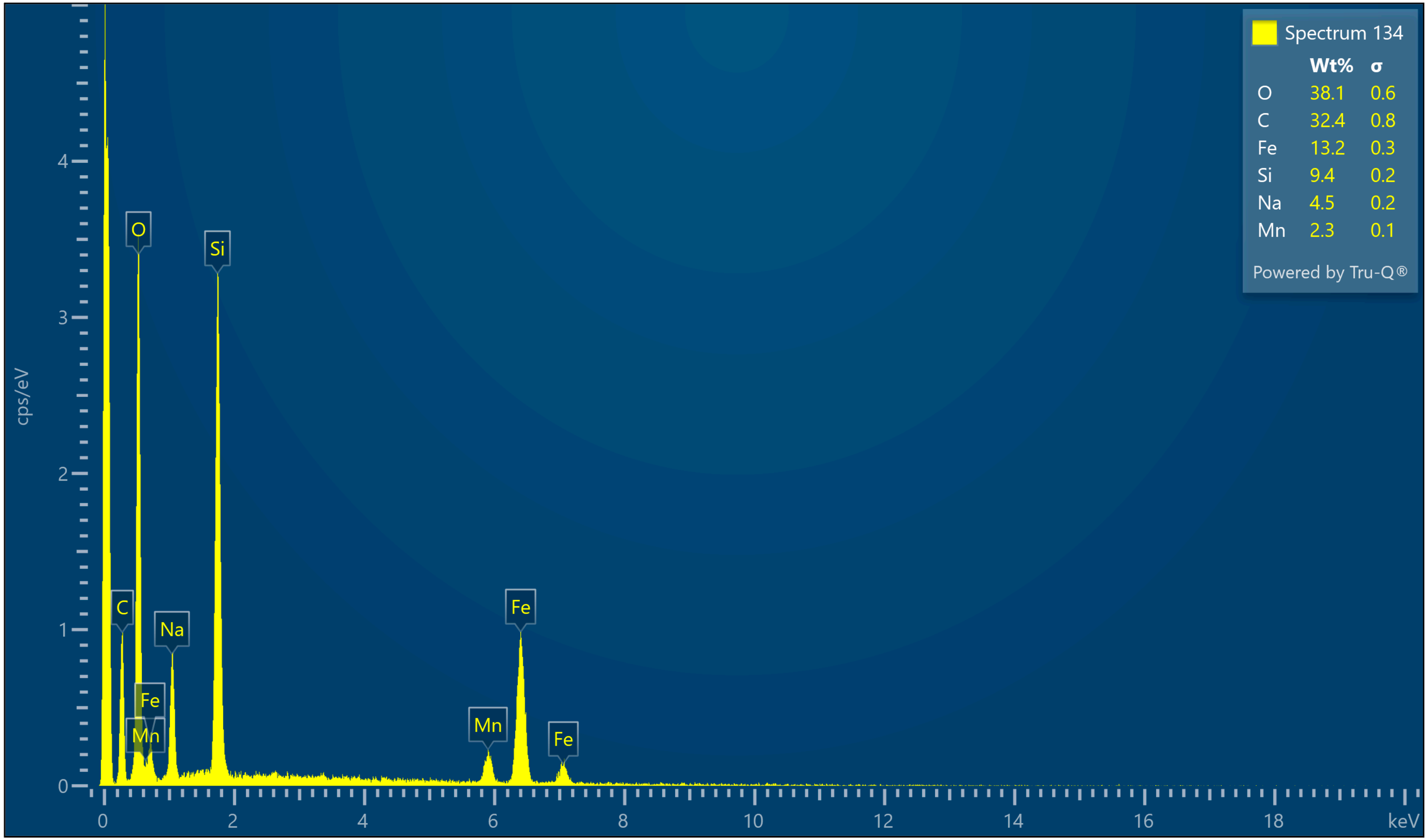
Spectrum 134
Wt% σ
O 38.1 0.6
C 32.4 0.8
Fe 13.2 0.3
Si 9.4 0.2
Na 4.5 0.2
Mn 2.3 0.1
Powered by Tru-Q®
cps/eV
0
1
2
3
4
O
Si
C
Na
Fe
Mn
Mn
Fe
Fe
0
2
4
6
8
10
12
14
16
18
keV

Figure S47

Table S47. Spectrum 134									
Element	Signal Type	Line	Apparent Concentration	k Ratio	Wt%	Wt% Sigma	Standard Name	Factory Standard	Standardization Date
C	EDS	K series	10.58	0.10577	32.43	0.85	C Vit	Yes	
O	EDS	K series	41.01	0.13801	38.12	0.64	SiO2	Yes	
Na	EDS	K series	4.51	0.01902	4.54	0.17	Albite	Yes	
Si	EDS	K series	10.98	0.08703	9.40	0.19	SiO2	Yes	
Mn	EDS	K series	2.60	0.02602	2.34	0.14	Mn	Yes	
Fe	EDS	K series	14.91	0.14911	13.18	0.31	Fe	Yes	
Total					100.00				

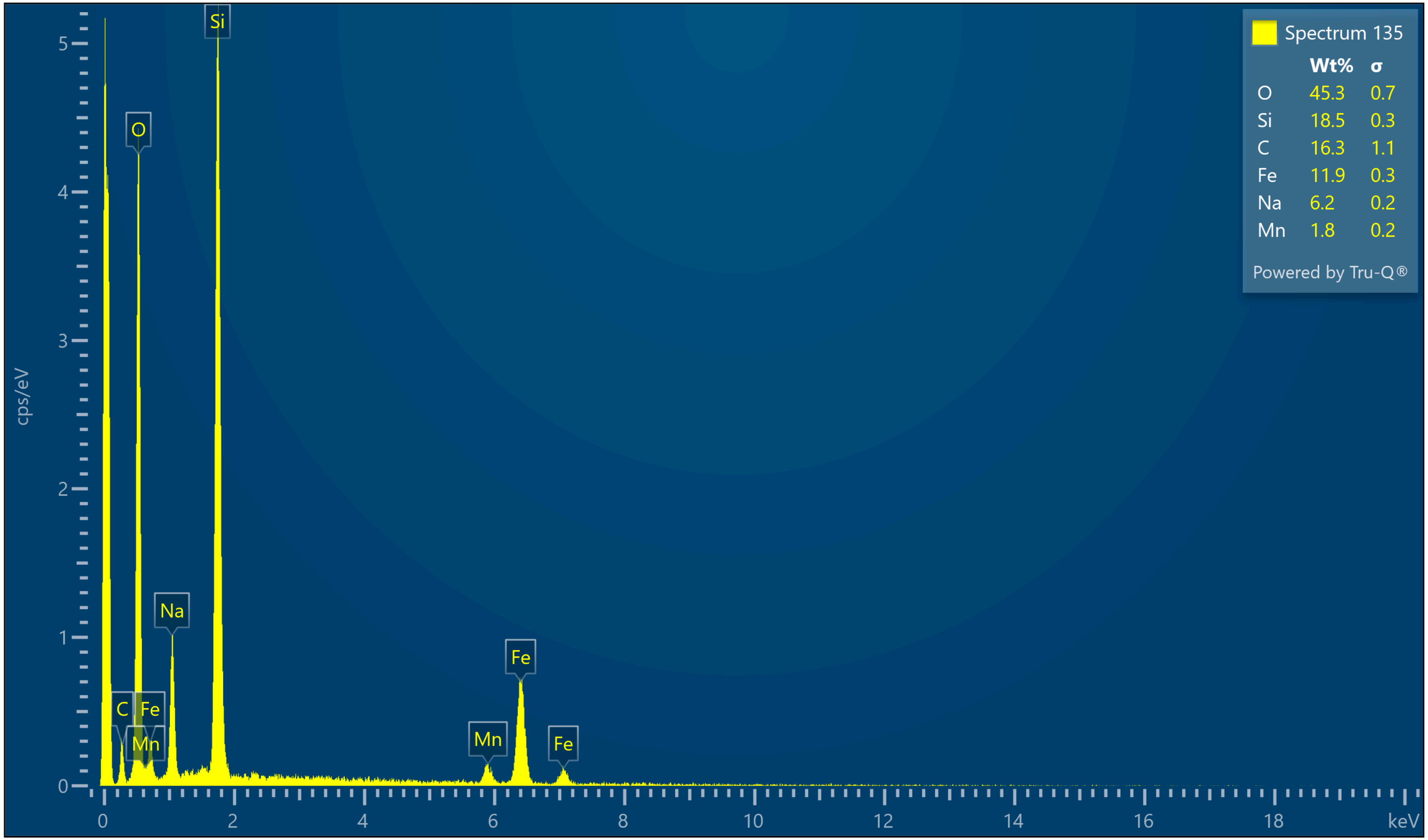

Figure S48

OXFORD INSTRUMENTS

Table S48. Spectrum 135

Element	Signal Type	Line	Apparent Concentration	k Ratio	Wt%	Wt% Sigma	Standard Name	Factory Standard	Standardization Date
C	EDS	K series	2.81	0.02806	16.26	1.09	C Vit	Yes	
O	EDS	K series	46.92	0.15788	45.28	0.75	SiO2	Yes	
Na	EDS	K series	4.84	0.02041	6.23	0.23	Albite	Yes	
Si	EDS	K series	16.51	0.13081	18.52	0.35	SiO2	Yes	
Mn	EDS	K series	1.52	0.01522	1.78	0.15	Mn	Yes	
Fe	EDS	K series	10.38	0.10380	11.94	0.34	Fe	Yes	
Total					100.00				

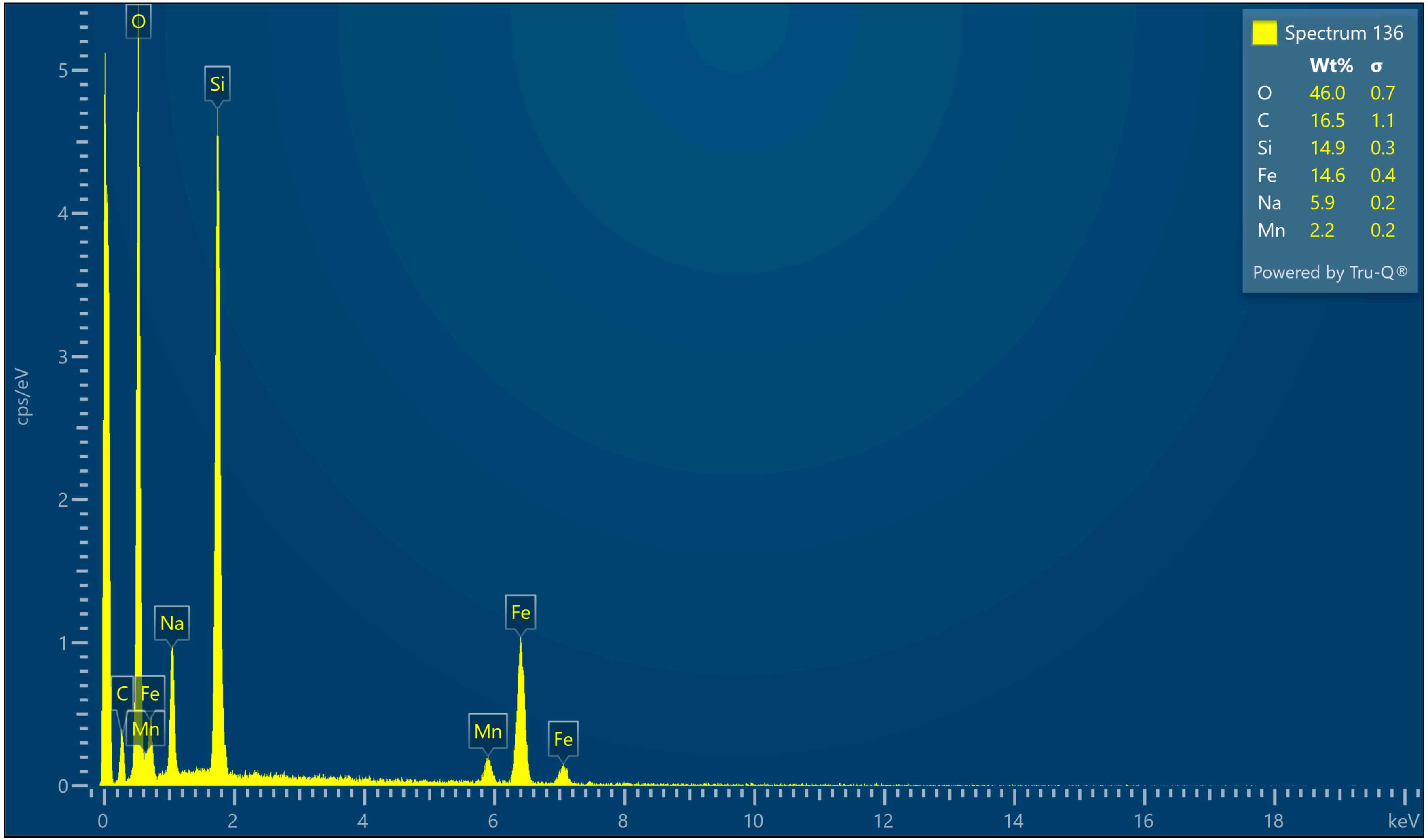
Spectrum 136
Wt% σ
O 46.0 0.7
C 16.5 1.1
Si 14.9 0.3
Fe 14.6 0.4
Na 5.9 0.2
Mn 2.2 0.2
Powered by Tru-Q®
cps/eV
keV
O
Si
Na
C
Fe
Mn
Fe
Mn
Fe

Figure S49

Table S49. Spectrum 136									
Element	Signal Type	Line	Apparent Concentration	k Ratio	Wt%	Wt% Sigma	Standard Name	Factory Standard	Standardization Date
C	EDS	K series	3.28	0.03278	16.45	1.07	C Vit	Yes	
O	EDS	K series	52.46	0.17652	45.97	0.74	SiO2	Yes	
Na	EDS	K series	4.50	0.01901	5.86	0.23	Albite	Yes	
Si	EDS	K series	13.64	0.10812	14.91	0.30	SiO2	Yes	
Mn	EDS	K series	1.94	0.01945	2.16	0.16	Mn	Yes	
Fe	EDS	K series	13.43	0.13428	14.65	0.37	Fe	Yes	
Total					100.00				

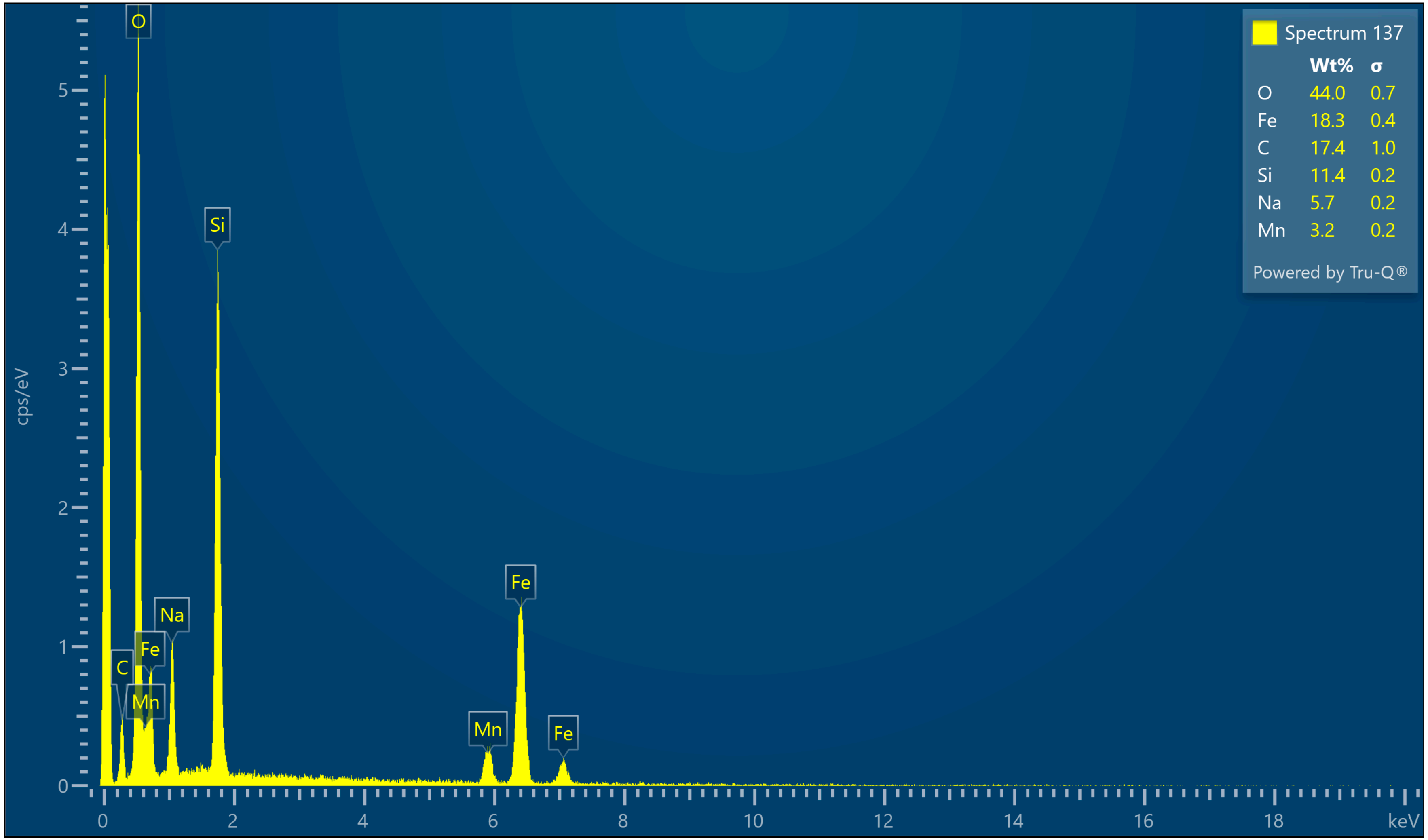
Spectrum 137
Wt% σ
O 44.0 0.7
Fe 18.3 0.4
C 17.4 1.0
Si 11.4 0.2
Na 5.7 0.2
Mn 3.2 0.2
Powered by Tru-Q®
cps/eV
keV
O
Si
Fe
Na
Fe
C
Mn
Mn
Fe
0
1
2
3
4
5
0
2
4
6
8
10
12
14
16
18

Figure S50

Table S50. Spectrum 137									
Element	Signal Type	Line	Apparent Concentration	k Ratio	Wt%	Wt% Sigma	Standard Name	Factory Standard	Standardization Date
C	EDS	K series	3.92	0.03916	17.40	1.03	C Vit	Yes	
O	EDS	K series	52.98	0.17829	44.02	0.72	SiO2	Yes	
Na	EDS	K series	4.28	0.01808	5.75	0.24	Albite	Yes	
Si	EDS	K series	10.45	0.08279	11.38	0.24	SiO2	Yes	
Mn	EDS	K series	2.98	0.02981	3.19	0.18	Mn	Yes	
Fe	EDS	K series	17.37	0.17371	18.26	0.41	Fe	Yes	
Total					100.00				

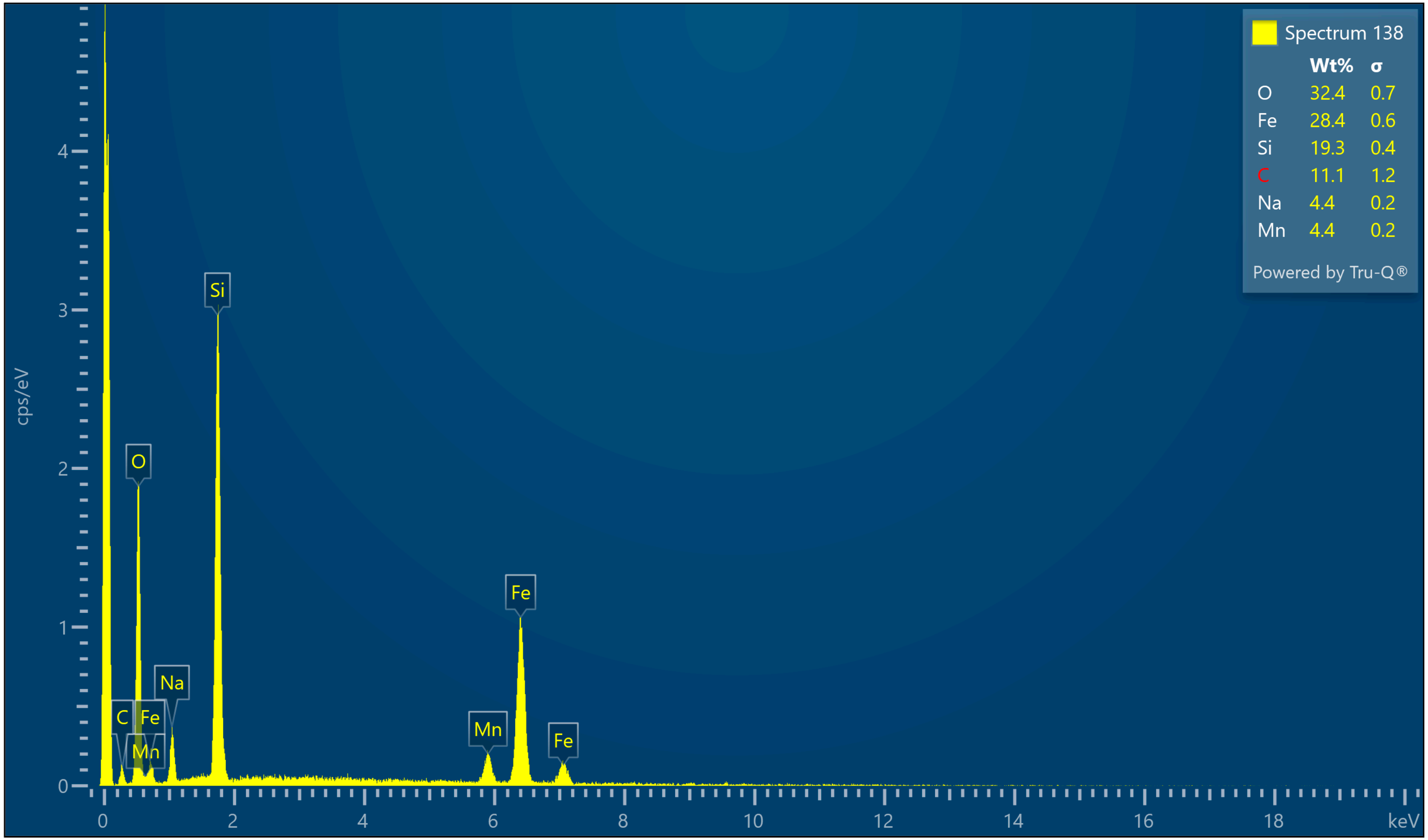
Spectrum 138
Wt% σ
O 32.4 0.7
Fe 28.4 0.6
Si 19.3 0.4
C 11.1 1.2
Na 4.4 0.2
Mn 4.4 0.2
Powered by Tru-Q®
cps/eV
0
1
2
3
4
Si
O
Na
C
Fe
Mn
Mn
Fe
Fe
0
2
4
6
8
10
12
14
16
18
keV

Figure S51

Table S51. Spectrum 138									
Element	Signal Type	Line	Apparent Concentration	k Ratio	Wt%	Wt% Sigma	Standard Name	Factory Standard	Standardization Date
C	EDS	K series	1.35	0.01354	11.08	1.20	C Vit	Yes	
O	EDS	K series	27.58	0.09282	32.42	0.67	SiO2	Yes	
Na	EDS	K series	2.19	0.00926	4.43	0.23	Albite	Yes	
Si	EDS	K series	12.35	0.09785	19.26	0.39	SiO2	Yes	
Mn	EDS	K series	2.98	0.02980	4.39	0.24	Mn	Yes	
Fe	EDS	K series	19.66	0.19662	28.42	0.58	Fe	Yes	
Total					100.00				

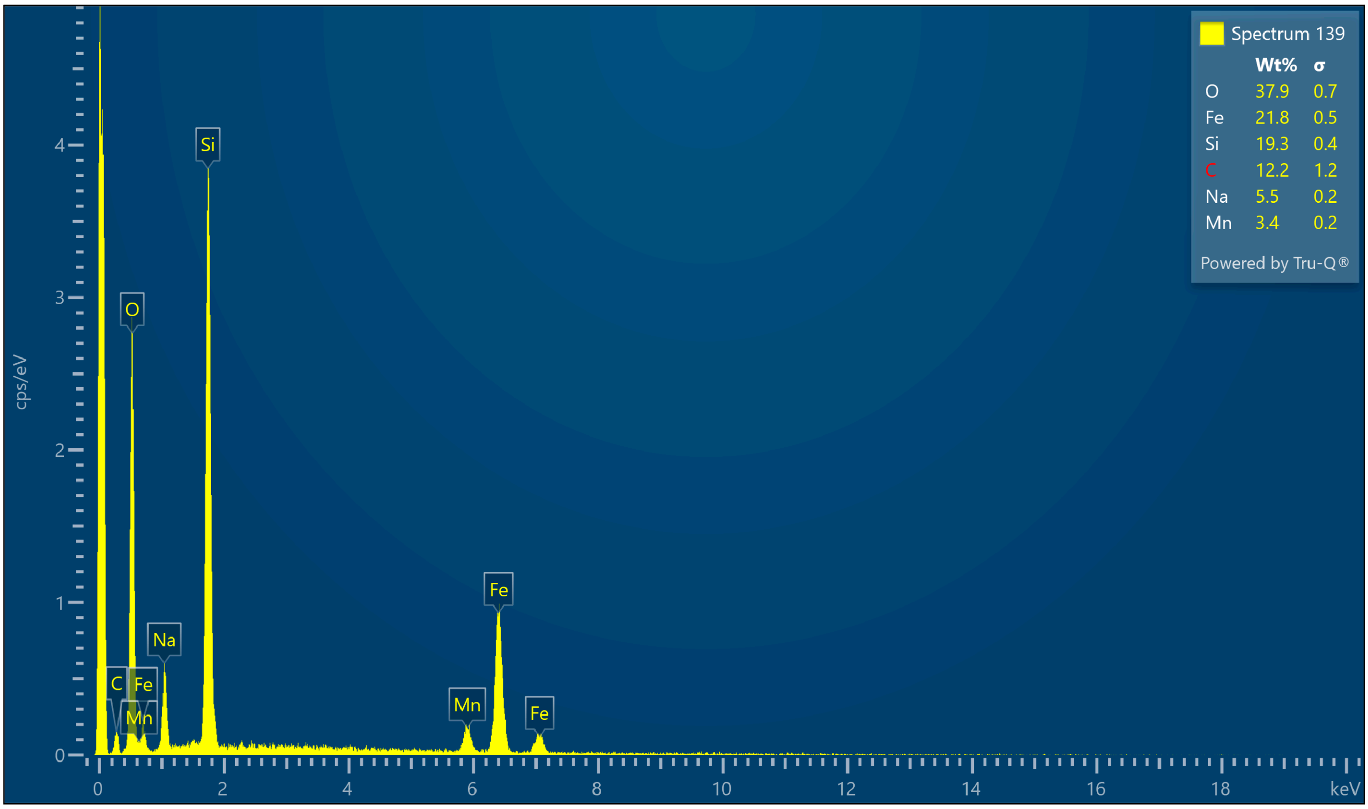

Figure S52

Table S52. Spectrum 139									
Element	Signal Type	Line	Apparent Concentration	k Ratio	Wt%	Wt% Sigma	Standard Name	Factory Standard	Standardization Date
C	EDS	K series	1.66	0.01656	12.18	1.16	C Vit	Yes	
O	EDS	K series	35.00	0.11776	37.89	0.71	SiO2	Yes	
Na	EDS	K series	3.18	0.01341	5.45	0.25	Albite	Yes	
Si	EDS	K series	13.90	0.11015	19.33	0.38	SiO2	Yes	
Mn	EDS	K series	2.47	0.02470	3.37	0.21	Mn	Yes	
Fe	EDS	K series	16.27	0.16272	21.78	0.49	Fe	Yes	
Total					100.00				

Electron Image 25

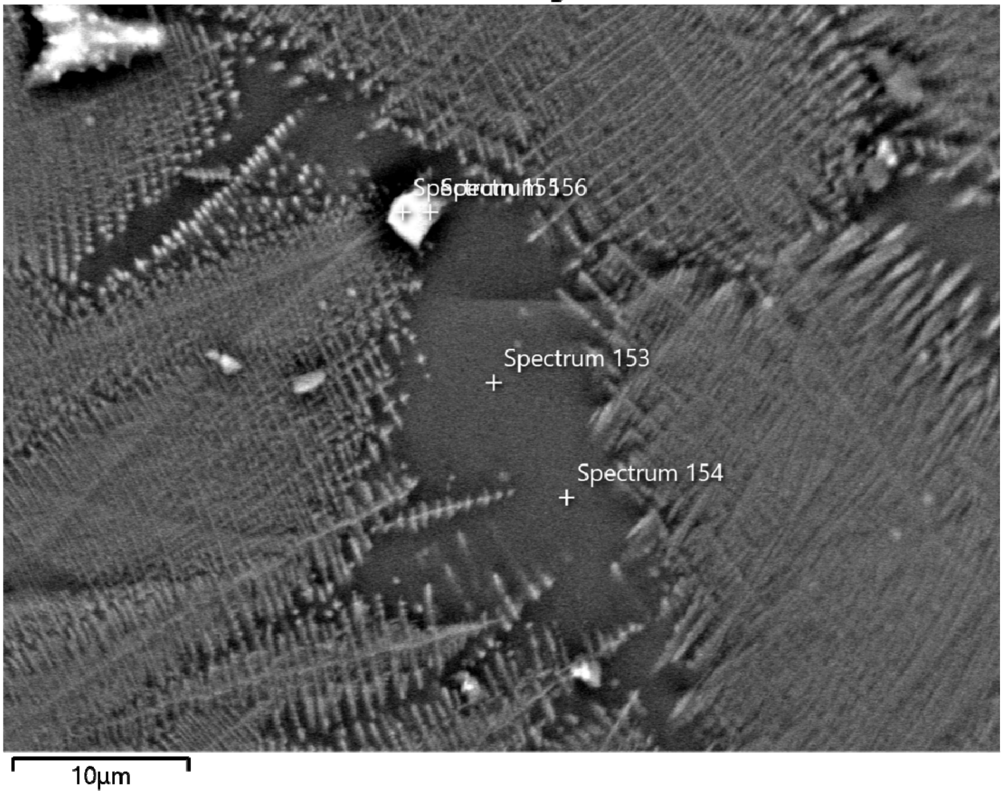

Figure S53

Table S53

	Fe	Mn	Ratio Fe/Mn		Ratio Fe/Mn end
153	40,44	5,11	7,913894	Glass	8
154	49,18	6,44	7,636646	Glass	8
155	27,61	3,69	7,482385	partile centre	8
156	22,94	3,5	6,554286	particle - periphery	7

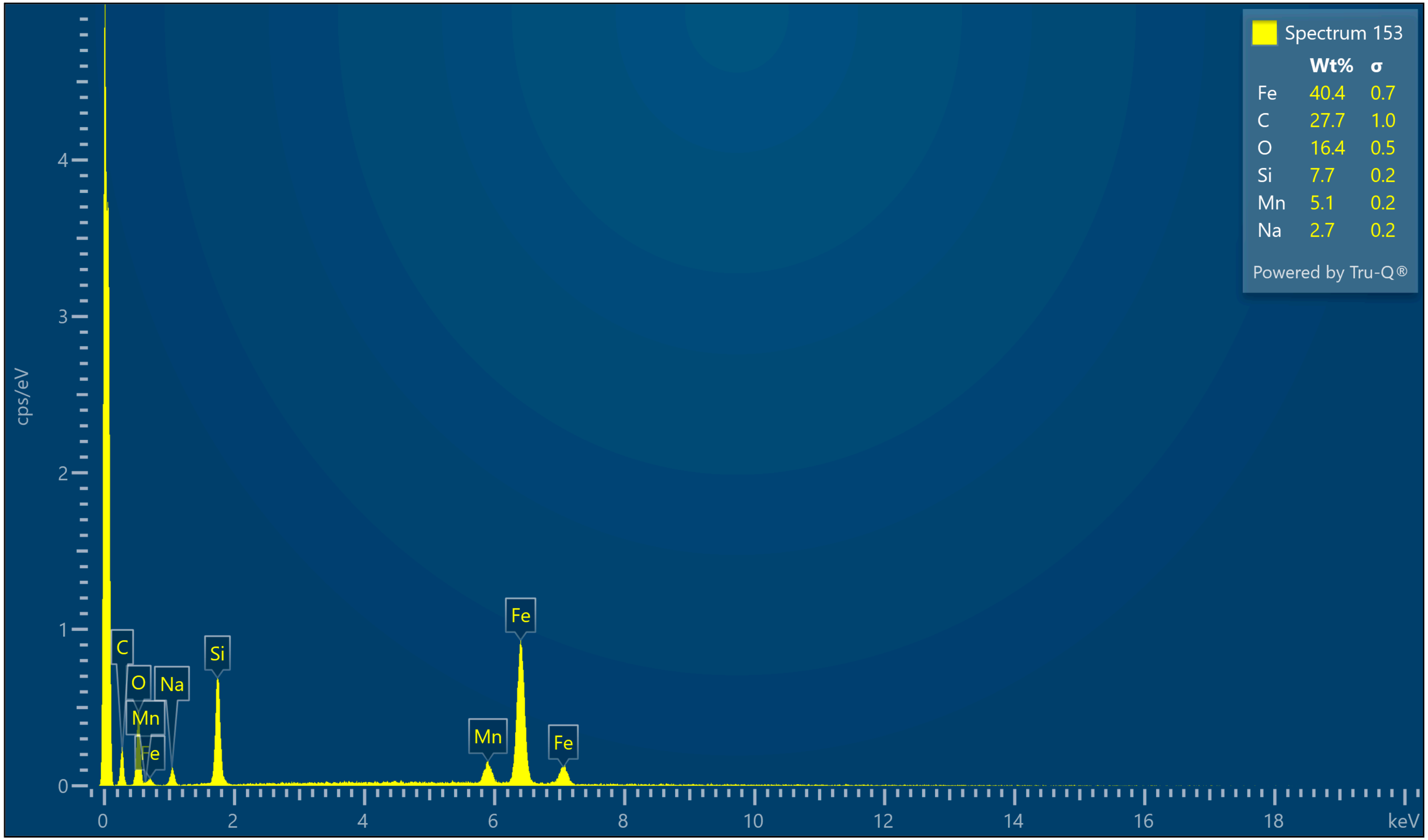
Spectrum 153
Wt% σ
Fe 40.4 0.7
C 27.7 1.0
O 16.4 0.5
Si 7.7 0.2
Mn 5.1 0.2
Na 2.7 0.2
Powered by Tru-Q®
cps/eV
keV
C
O
Na
Mn
Fe
Si
Mn
Fe
Fe

Figure S54

Table S54. Spectrum 153

Element	Signal Type	Line	Apparent Concentration	k Ratio	Wt%	Wt% Sigma	Standard Name	Factory Standard	Standardization Date
C	EDS	K series	4.48	0.04485	27.65	0.98	C Vit	Yes	
O	EDS	K series	10.62	0.03575	16.40	0.47	SiO2	Yes	
Na	EDS	K series	1.12	0.00473	2.66	0.17	Albite	Yes	
Si	EDS	K series	4.54	0.03595	7.75	0.19	SiO2	Yes	
Mn	EDS	K series	3.35	0.03353	5.11	0.24	Mn	Yes	
Fe	EDS	K series	27.03	0.27033	40.44	0.67	Fe	Yes	
Total					100.00				

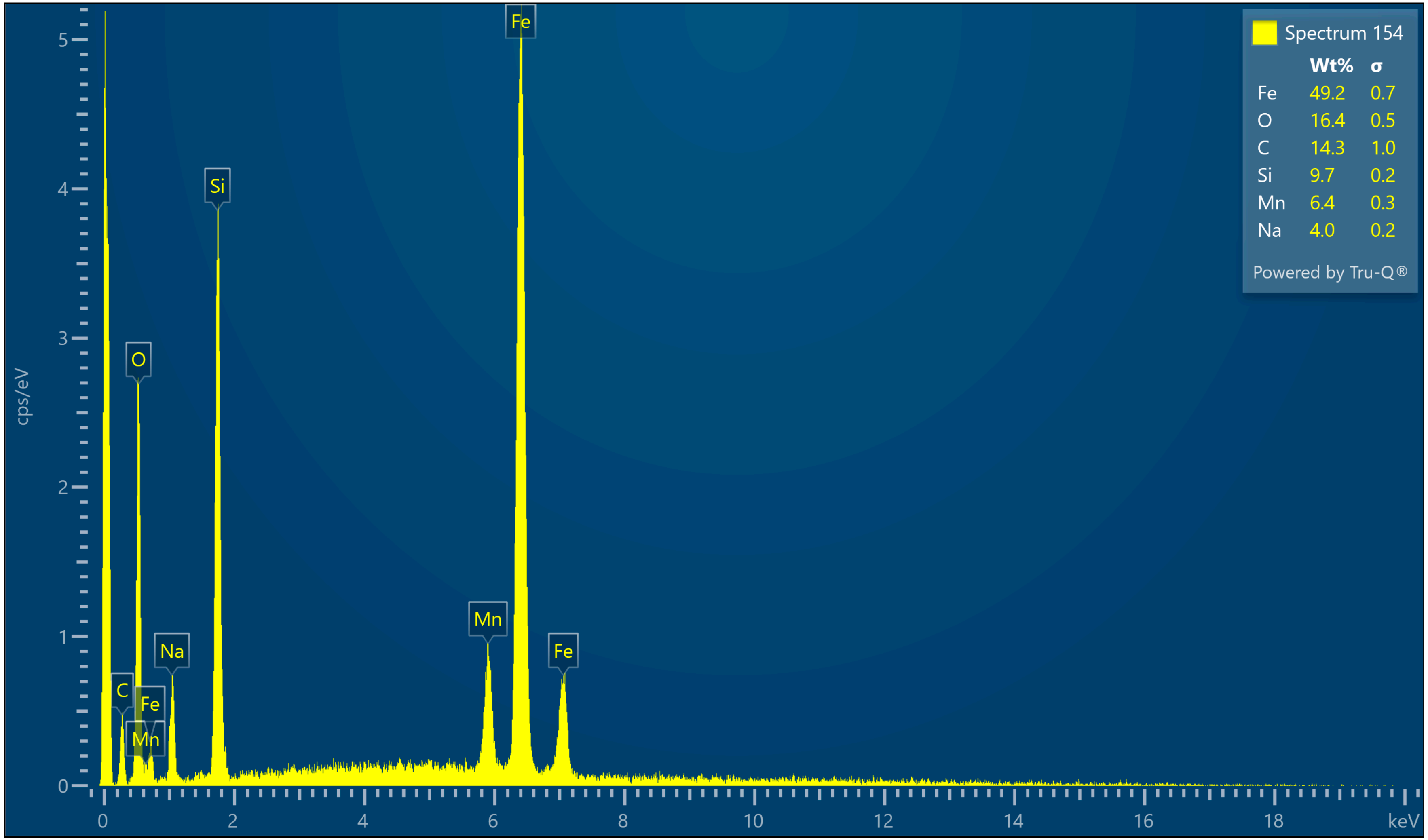

Spectrum 154
Wt% σ
Fe 49.2 0.7
O 16.4 0.5
C 14.3 1.0
Si 9.7 0.2
Mn 6.4 0.3
Na 4.0 0.2
Powered by Tru-Q®
cps/eV
0 1 2 3 4 5
0 2 4 6 8 10 12 14 16 18 keV
Fe
Si
O
Mn
Na
Fe
C
Fe
Mn

Figure S55

Table S55. Spectrum 154									
Element	Signal Type	Line	Apparent Concentration	k Ratio	Wt%	Wt% Sigma	Standard Name	Factory Standard	Standardization Date
C	EDS	K series	2.70	0.02703	14.26	1.03	C Vit	Yes	
O	EDS	K series	18.41	0.06194	16.44	0.45	SiO2	Yes	
Na	EDS	K series	2.14	0.00903	4.03	0.24	Albite	Yes	
Si	EDS	K series	7.36	0.05829	9.66	0.23	SiO2	Yes	
Mn	EDS	K series	5.94	0.05938	6.44	0.29	Mn	Yes	
Fe	EDS	K series	46.28	0.46283	49.18	0.74	Fe	Yes	
Total					100.00				

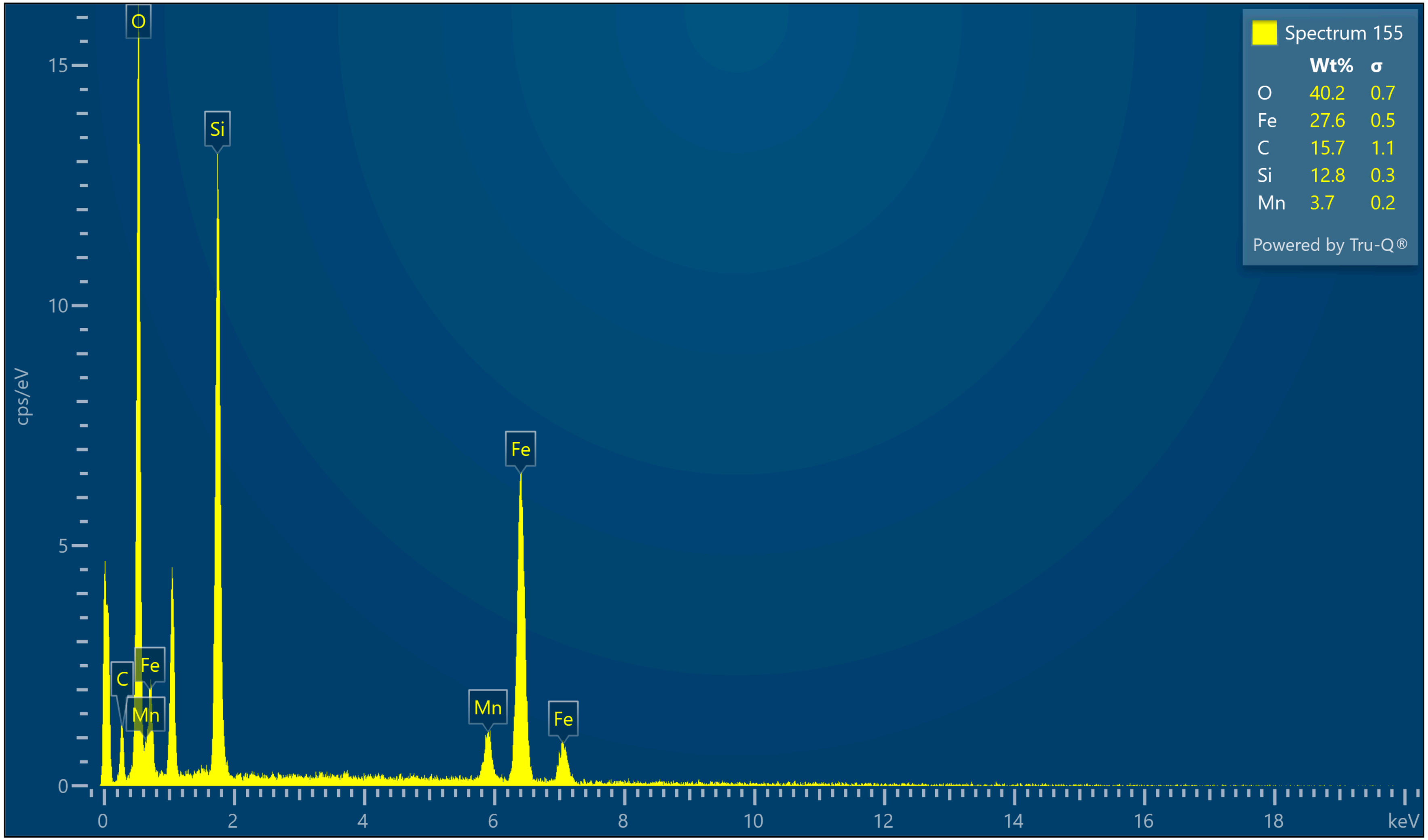

Figure S56

OXFORD INSTRUMENTS

Table S56. Spectrum 155

Element	Signal Type	Line	Apparent Concentration	k Ratio	Wt%	Wt% Sigma	Standard Name	Factory Standard	Standardization Date
C	EDS	K series	3.60	0.03597	15.68	1.06	C Vit	Yes	
O	EDS	K series	51.92	0.17471	40.24	0.70	SiO2	Yes	
Si	EDS	K series	12.29	0.09736	12.77	0.27	SiO2	Yes	
Mn	EDS	K series	3.70	0.03699	3.69	0.22	Mn	Yes	
Fe	EDS	K series	28.15	0.28146	27.61	0.54	Fe	Yes	
Total					100.00				

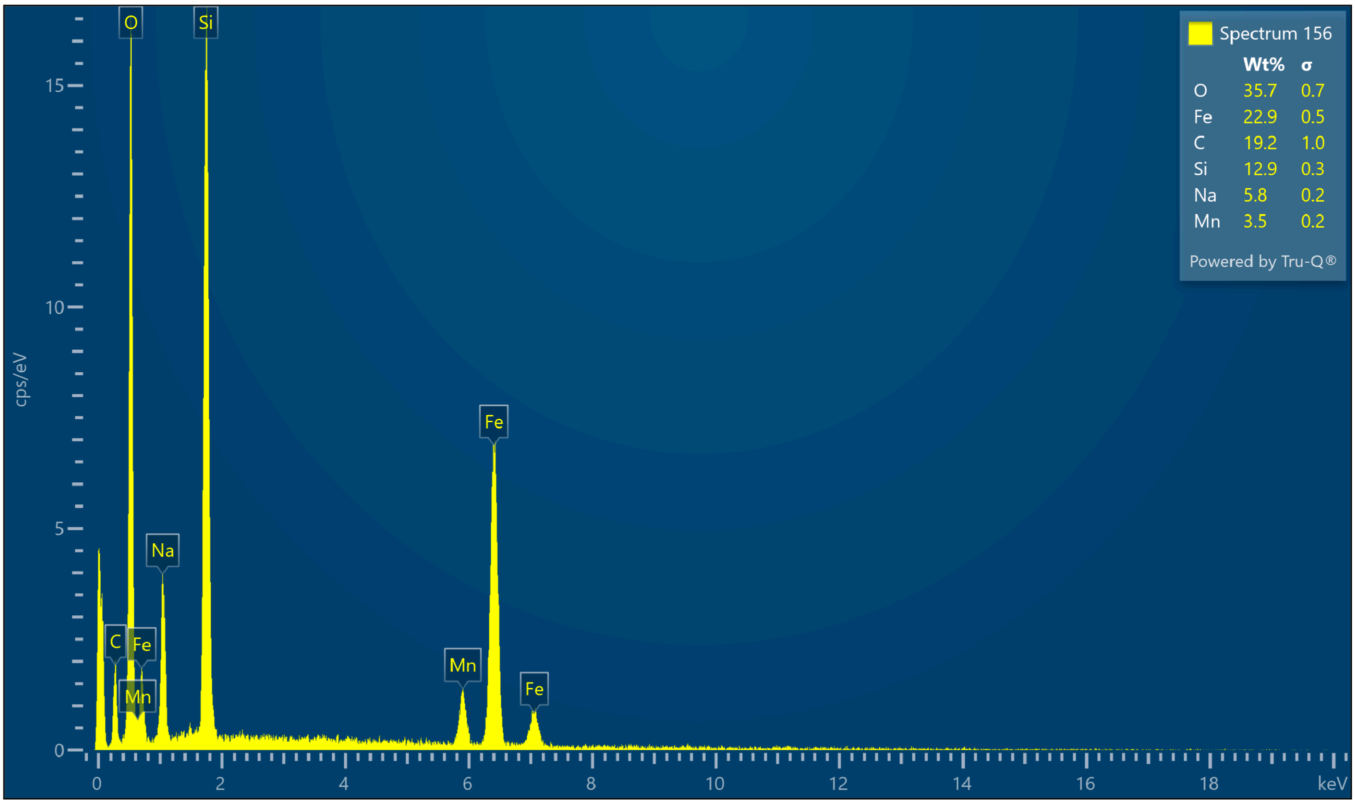

Figure S57

Table S57. Spectrum 156									
Element	Signal Type	Line	Apparent Concentration	k Ratio	Wt%	Wt% Sigma	Standard Name	Factory Standard	Standardization Date
C	EDS	K series	4.93	0.04930	19.18	1.04	C Vit	Yes	
O	EDS	K series	48.11	0.16188	35.73	0.65	SiO2	Yes	
Na	EDS	K series	5.15	0.02172	5.78	0.23	Albite	Yes	
Si	EDS	K series	14.24	0.11286	12.86	0.27	SiO2	Yes	
Mn	EDS	K series	4.02	0.04015	3.50	0.19	Mn	Yes	
Fe	EDS	K series	26.78	0.26780	22.94	0.47	Fe	Yes	
Total					100.00				

Sample 25FR

,

Electron Image 20

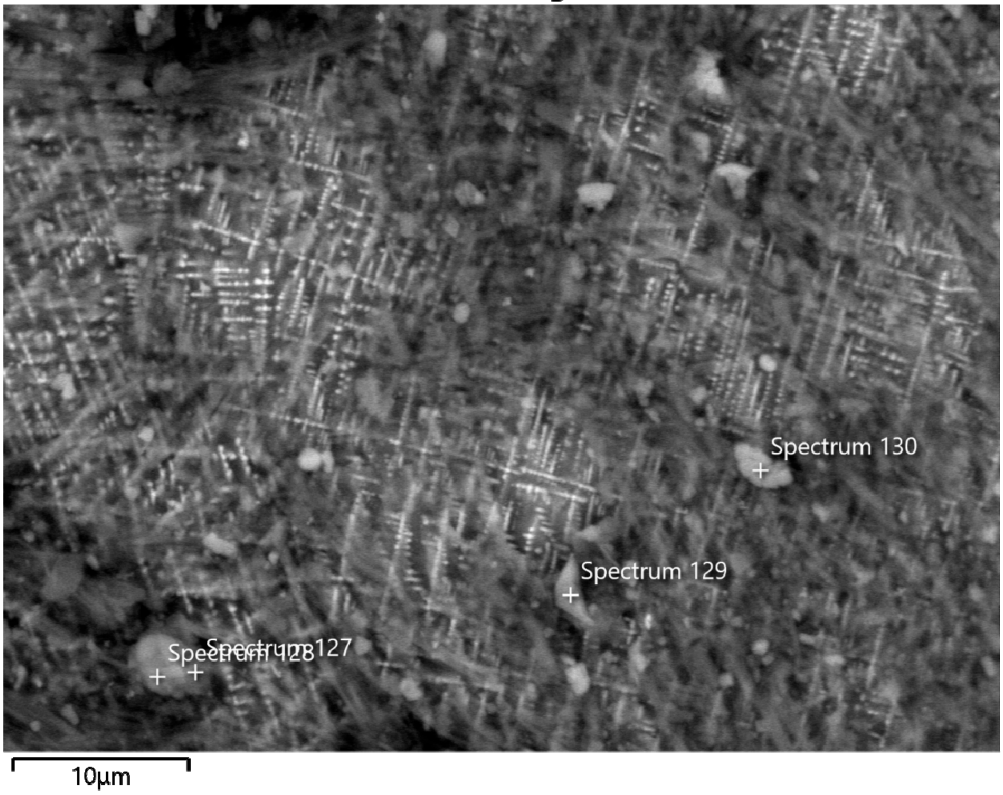

Figure S58

Table S58

	Fe	Mn	Ratio Fe/Mn		Ratio Fe/Mn end
127	16,66	2,5	6,664	partile	7
128	2,63	0,5	5,26	particle -	5
129	5,13	0,7	7,328571	particle	7
130	7,16	1,31	5,465649	particle	6
153	40,44	5,11	7,913894	Glass	8
154	49,18	6,44	7,636646	Glass	8

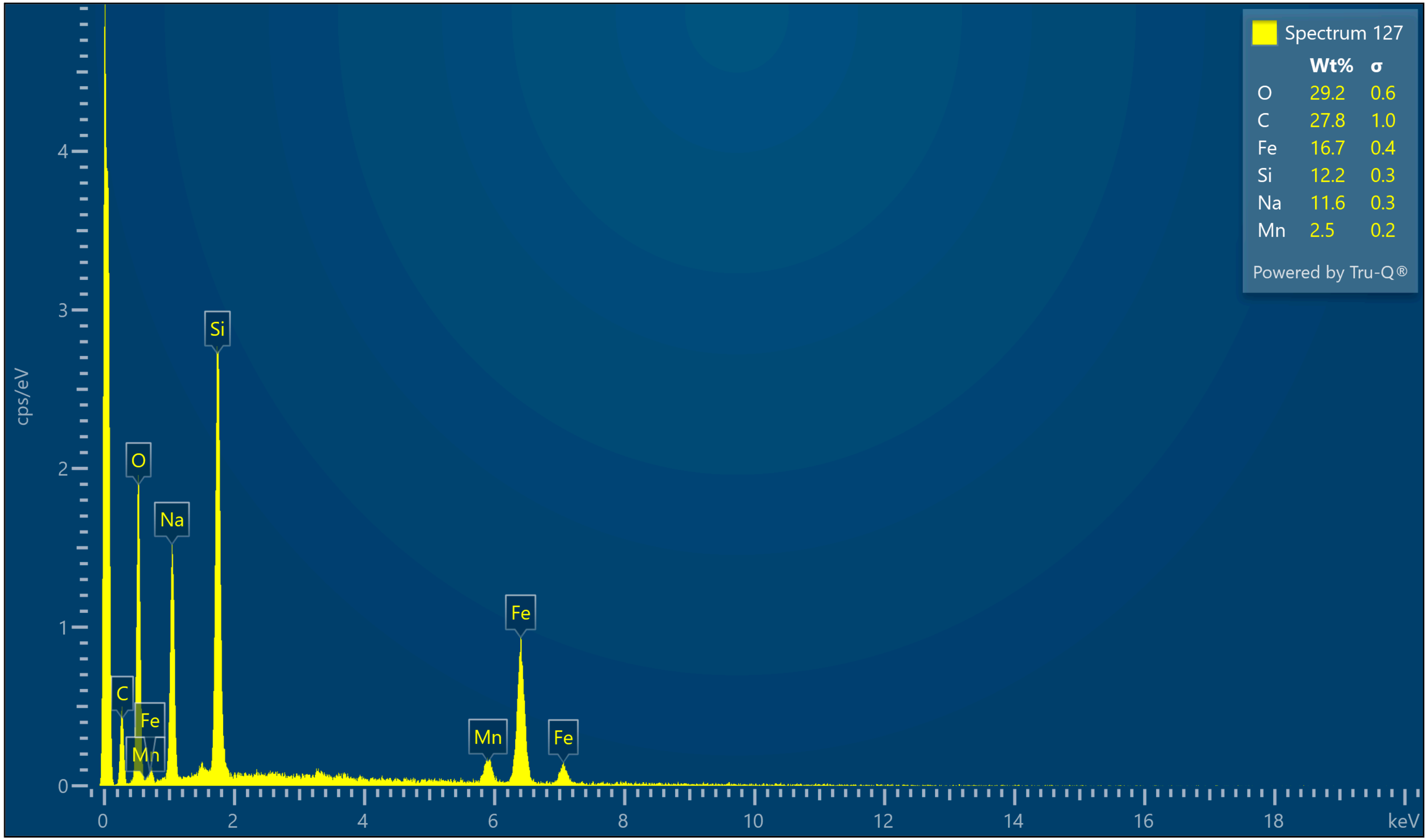

Figure S59

OXFORD INSTRUMENTS

Table S59. Spectrum 127

Element	Signal Type	Line	Apparent Concentration	k Ratio	Wt%	Wt% Sigma	Standard Name	Factory Standard	Standardization Date
C	EDS	K series	5.71	0.05713	27.84	1.02	C Vit	Yes	
O	EDS	K series	25.28	0.08506	29.16	0.62	SiO2	Yes	
Na	EDS	K series	9.41	0.03972	11.60	0.30	Albite	Yes	
Si	EDS	K series	10.75	0.08522	12.24	0.26	SiO2	Yes	
Mn	EDS	K series	2.22	0.02218	2.50	0.18	Mn	Yes	
Fe	EDS	K series	15.02	0.15020	16.66	0.40	Fe	Yes	
Total					100.00				

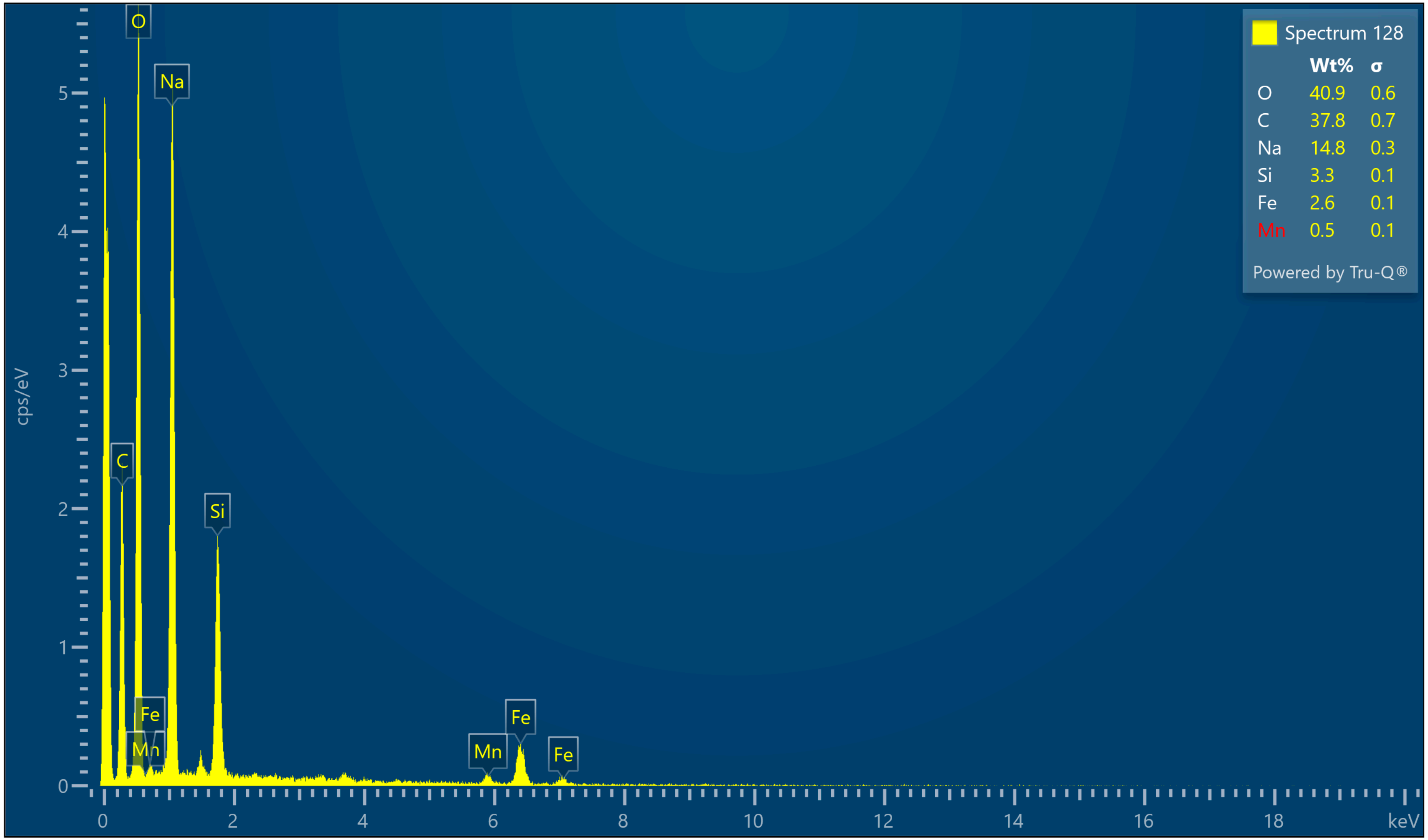
Spectrum 128
Wt% σ
O 40.9 0.6
C 37.8 0.7
Na 14.8 0.3
Si 3.3 0.1
Fe 2.6 0.1
Mn 0.5 0.1
Powered by Tru-Q®
cps/eV
keV
O
Na
C
Si
Fe
Mn
Fe
Mn
Fe

Figure S60

Table S60. pectrum 128

Element	Signal Type	Line	Apparent Concentration	k Ratio	Wt%	Wt% Sigma	Standard Name	Factory Standard	Standardization Date
C	EDS	K series	21.05	0.21050	37.84	0.66	C Vit	Yes	
O	EDS	K series	56.60	0.19046	40.90	0.56	SiO2	Yes	
Na	EDS	K series	24.55	0.10360	14.79	0.25	Albite	Yes	
Si	EDS	K series	5.23	0.04141	3.34	0.09	SiO2	Yes	
Mn	EDS	K series	0.75	0.00746	0.50	0.07	Mn	Yes	
Fe	EDS	K series	3.97	0.03972	2.63	0.13	Fe	Yes	
Total					100.00				

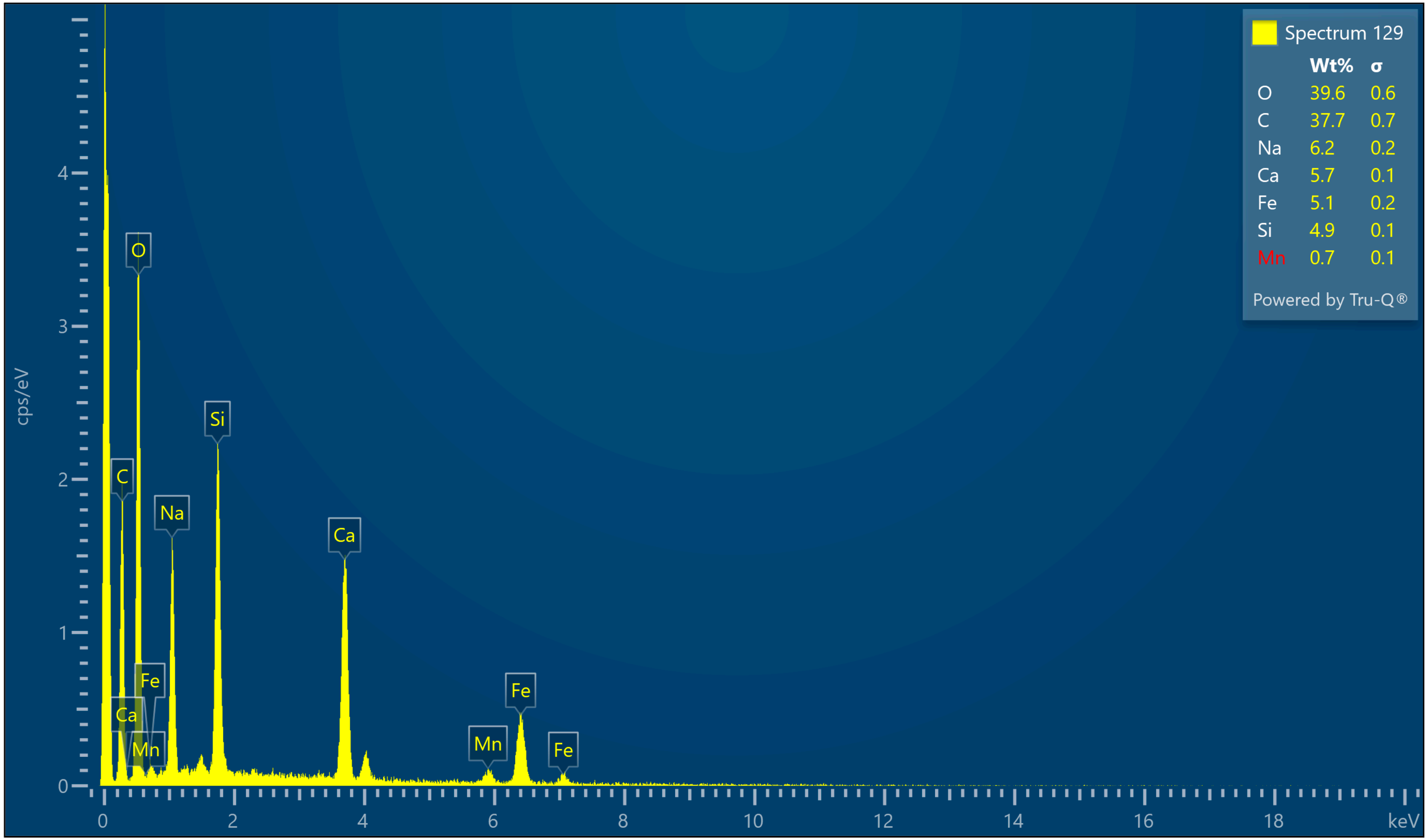
Spectrum 129
Wt% σ
O 39.6 0.6
C 37.7 0.7
Na 6.2 0.2
Ca 5.7 0.1
Fe 5.1 0.2
Si 4.9 0.1
Mn 0.7 0.1
Powered by Tru-Q®
cps/eV
keV
O
C
Na
Si
Ca
Fe
Mn

Figure S61

Table S61. Spectrum 129

Element	Signal Type	Line	Apparent Concentration	k Ratio	Wt%	Wt% Sigma	Standard Name	Factory Standard	Standardization Date
C	EDS	K series	19.00	0.18997	37.69	0.74	C Vit	Yes	
O	EDS	K series	38.39	0.12918	39.64	0.63	SiO2	Yes	
Na	EDS	K series	7.87	0.03320	6.16	0.18	Albite	Yes	
Si	EDS	K series	6.88	0.05453	4.92	0.12	SiO2	Yes	
Ca	EDS	K series	9.21	0.08233	5.75	0.14	Wollastonite	Yes	
Mn	EDS	K series	0.91	0.00907	0.71	0.09	Mn	Yes	
Fe	EDS	K series	6.63	0.06626	5.13	0.19	Fe	Yes	
Total					100.00				

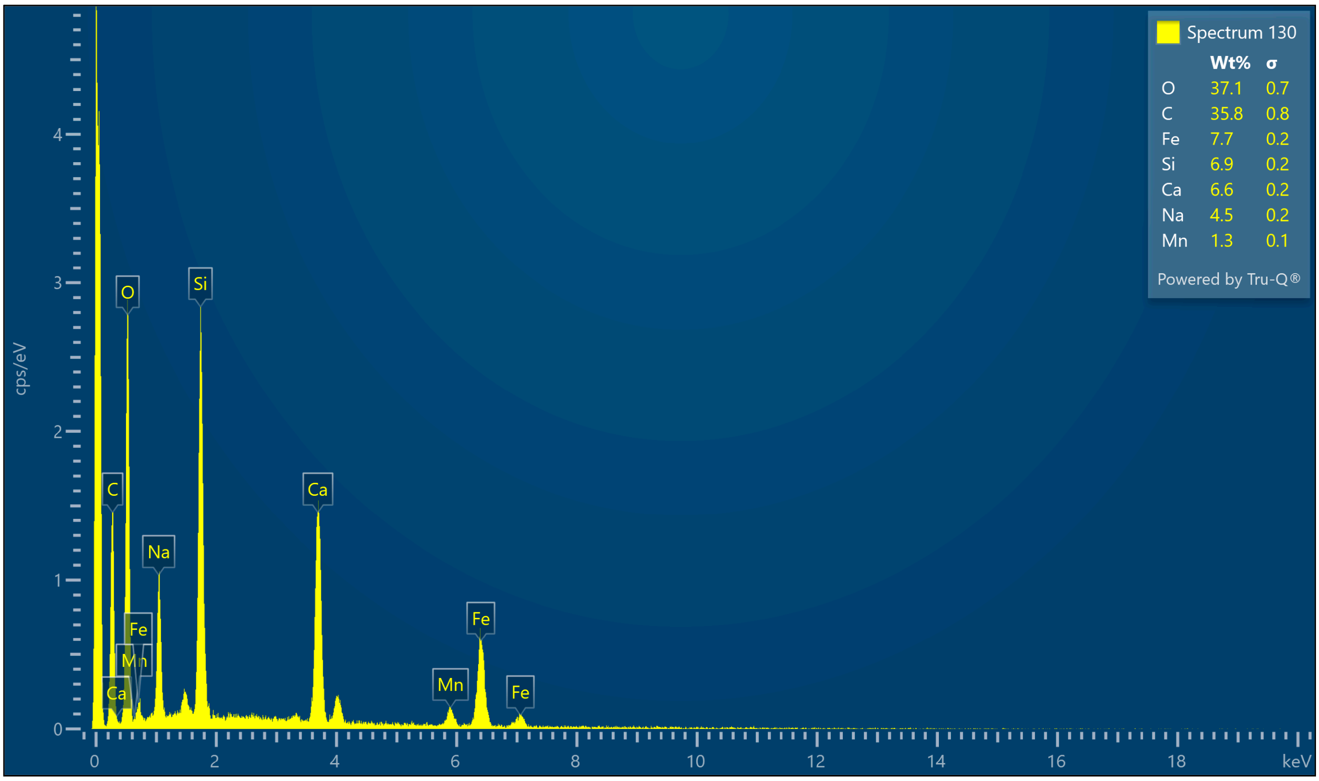

Figure S62

Table S62. Spectrum 130

Element	Signal Type	Line	Apparent Concentration	k Ratio	Wt%	Wt% Sigma	Standard Name	Factory Standard	Standardization Date
C	EDS	K series	14.48	0.14479	35.78	0.83	C Vit	Yes	
O	EDS	K series	31.59	0.10630	37.14	0.67	SiO2	Yes	
Na	EDS	K series	4.98	0.02101	4.55	0.17	Albite	Yes	
Si	EDS	K series	8.62	0.06833	6.93	0.16	SiO2	Yes	
Ca	EDS	K series	9.45	0.08441	6.61	0.16	Wollastonite	Yes	
Mn	EDS	K series	1.49	0.01488	1.31	0.12	Mn	Yes	
Fe	EDS	K series	8.86	0.08865	7.68	0.24	Fe	Yes	
Total					100.00				
